# Myeloid-derived growth factor alleviates non-alcoholic fatty liver disease alleviates in a manner involving IKKβ/NF-κB signaling

**DOI:** 10.1038/s41419-023-05904-y

**Published:** 2023-06-26

**Authors:** Yan Ding, Xiaoli Xu, Biying Meng, Li Wang, Biao Zhu, Bei Guo, Jiajia Zhang, Lin Xiang, Jing Dong, Min Liu, Guangda Xiang

**Affiliations:** 1 Department of Endocrinology, General Hospital of Central Theater Command, Wuluo Road 627, Wuhan, 430070 Hubei Province China; 2 Department of Diagnostics, School of Medicine, Hunan University of Medicine, Huaihua, 418000 Hunan Province China; 3 The First School of Clinical Medicine, Southern Medical University, NO.1023, South Shatai Road, Guangzhou, 510515 Guangdong Province China

**Keywords:** Non-alcoholic fatty liver disease, Mechanisms of disease, Obesity

## Abstract

Whether bone marrow modulates systemic metabolism remains unknown. Our recent study suggested that myeloid-derived growth factor (MYDGF) improves insulin resistance. Here, we found that myeloid cell-specific MYDGF deficiency aggravated hepatic inflammation, lipogenesis, and steatosis, and show that myeloid cell-derived MYDGF restoration alleviated hepatic inflammation, lipogenesis, and steatosis. Additionally, recombinant MYDGF attenuated inflammation, lipogenesis, and fat deposition in primary mouse hepatocytes (PMHs). Importantly, inhibitor kappa B kinase beta/nuclear factor-kappa B (IKKβ/NF-κB) signaling is involved in protection of MYDGF on non-alcoholic fatty liver disease (NAFLD). These data revealed that myeloid cell-derived MYDGF alleviates NAFLD and inflammation in a manner involving IKKβ/NF-κB signaling, and serves as a factor involved in the crosstalk between the liver and bone marrow that regulates liver fat metabolism. Bone marrow functions as an endocrine organ and serves as a potential therapeutic target for metabolic disorders.

## Introduction

Non-alcoholic fatty liver disease (NAFLD) represents a spectrum of diseases that includes simple steatosis, steatohepatitis and cirrhosis [1]. As NAFLD is associated with inflammation, insulin resistance (IR) and dyslipidemia, it leads to excessive mortality and health expenditures [2]. However, there are currently no effective treatments to alleviate NAFLD [3, 4]. Therefore, there is an urgent need to explore new effective treatments for NAFLD to prolong lifespan and reduce mortality.

Inflammation plays a key role in the development of NAFLD [5]. Inflammation can increase the amount and transcriptional activity of sterol regulatory element-binding protein 1c (SREBP1c), promote liver lipogenesis, and aggravate the development of NAFLD [6, 7]. Inhibition of inflammation alleviates the progression of NAFLD [8]. Therefore, anti-inflammatory approaches for the treatment of NAFLD have gained much attention in recent years [9].

Bone marrow serves as a hematopoietic organ and plays an important role in human health. However, little is known about its other physiologic functions, including its endocrine functions. Interestingly, our recent study demonstrated that myeloid-derived growth factor (MYDGF), a protein secreted from bone marrow-derived monocytes and macrophages [10], improved IR and glucose-lipid metabolic profiles in diabetic mice [11]. Metabolic disorders have also been shown to be closely associated with NAFLD [12, 13]. Therefore, in this study, we first aimed to ascertain whether MYDGF can modulate NAFLD and the possible underlying mechanisms, before exploring whether MYDGF serves as a factor involved in the crosstalk between bone marrow and liver that regulates liver fat metabolism.

## Results

### Decreased MYDGF levels and increased inflammation in NAFLD patients and mice

**Fig. 1:**
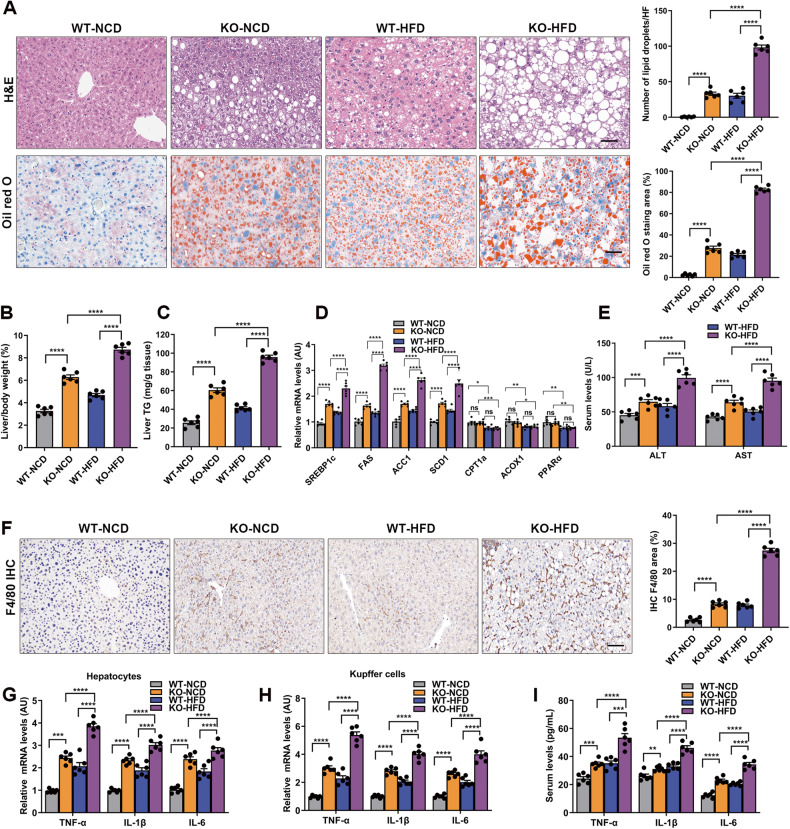
Myeloid cell-specific MYDGF deficiency exacerbated inflammation, lipogenesis, and hepatic steatosis in NAFLD mice. KO and WT mice aged 4–6 weeks were divided into two groups (WT and KO) and fed a HFD for 12 weeks (*n* = 6 mice per group). The control group indicates WT mice fed a NCD. **A** Representative images showing HE staining and oil red O staining of liver sections (*n* = 6). **B** Liver/body weight (*n* = 6). **C** Hepatic TG content (*n* = 6). **D** mRNA levels of genes related to fat metabolism in isolated hepatocytes (*n* = 6). **E** Serum ALT and AST levels (*n* = 6). **F** Representative images showing F4/80 staining of liver sections (*n* = 6). **G** Levels of inflammatory factors in isolated hepatocytes (*n* = 6). **H** Levels of inflammatory factors in KCs (*n* = 6). **I** Levels of inflammatory factors in serum (*n* = 6). Data are presented as the mean ± SEM. Black scale bar: 50 μm. ^*^P < 0.05, ^**^P < 0.01, ^***^P < 0.001, ^****^P < 0.0001. Significant differences were determined by Student’s *t*-test or one-way ANOVA followed by Tukey’s post-test.

Our previous study found that MYDGF expression declined in diabetic male mice [11]. In the current study, we demonstrated that patients with NAFLD had 25.0% lower serum MYDGF levels than healthy controls. Moreover, in patients with NAFLD, serum MYDGF levels were negatively correlated with the severity of NAFLD (Table S1, Fig. S1A, *P* < 0.001). Accordingly, serum MYDGF levels were 35.1% lower in NAFLD mice than in control mice, and the serum MYDGF level was also negatively correlated with the severity of NAFLD in mice with NAFLD (Fig. S1B and Table S2, *P* < 0.001). In addition, the results of western blot (WB), reverse transcription-polymerase chain (RT-PCR), and immunofluorescent (IF) experiments showed that the MYDGF protein and mRNA expression levels were lower in the marrow of NAFLD mice than in control mice (Fig. 1C–E, *P* < 0.001). These data indicate that MYDGF may be associated with NAFLD.

Obesity-related inflammation is a critical factor in the triggering or aggravation of NAFLD [14]. Likewise, the levels of circulating tumor necrosis factor-α (TNF-α), interleukin-1β (IL-1β), and IL-6 in both patients and mice with NAFLD were significantly higher than those in controls (Tables S1, S2, *P* < 0.001). Consistently, the mRNA levels of inflammatory factors in the liver were significantly increased in NAFLD mice compared to control mice (Fig. S1F, *P* < 0.0001). Interestingly, serum MYDGF levels were negatively correlated with several serum inflammatory factors in both patients and mice with NAFLD (Fig. S1G, H). These results indicate a correlation between MYDGF and inflammation in NAFLD.

In accordance with our previous studies [11], impaired metabolic disorders were found in patients and mice with NAFLD; these included higher body mass index (BMI), systolic blood pressure (SBP), homeostasis model assessment of insulin resistance (HOMA-IR), aspartate aminotransferase (AST), alanine aminotransferase (ALT), TNF-α, IL-1β, IL-6, triglyceride (TG), total cholesterol (TC), low-density lipoprotein cholesterol (LDL-C), serum free fatty acid (FFA), and fasting serum insulin levels (Tables S1, S2).

### Myeloid cell specific MYDGF deficiency exacerbated inflammation, lipogenesis and hepatic steatosis in NAFLD mice

First, we sought to explore the bone marrow integrity in the peripheral blood and bone marrow of myeloid cell-specific MYDGF knockout (KO) mice. The analysis of peripheral blood cells and distributions of nuclues in both bone marrow and cortical bone from toluidine blue-stained femur sections showed no significant differences between wild-type (WT) and KO mice (Table S3, Fig. S2B), indicating that the bone marrow integrity is maintained after myeloid cell-specific MYDGF knockout in mice. Second, MYDGF expression was mostly knocked out in whole bone marrow cells of KO mice, especially in macrophages and monocytes, but with no obvious differences in lymphocytes and bone marrow stromal cells (Fig. S2C, *P* < 0.0001). Third, no significant differences in MYDGF expression were found in the lung, heart, kidney, liver, or spleen between WT and KO mice (Fig. S2D, *P* > 0.05), confirming myeloid cell-specific MYDGF knockout.

Next, we sought to address the effects of myeloid cell-specific MYDGF deficiency on NAFLD in mice. The severity of high-fat diet (HFD)-induced hepatic steatosis was greater in KO mice than in WT mice, as evidenced by an increase in liver lipid droplets, liver lipid deposition area (Fig. 1A, *P* < 0.0001), liver index (Fig. 1B, *P* < 0.0001), and hepatic TG content (Fig. 1C, *P* < 0.0001) in KO mice. Importantly, the mRNA expression of the lipid synthesis genes SREBP1c, acetyl-CoA carboxylase 1 (ACC1), fatty acid synthase (FAS), and stearoyl-CoA desaturase 1 (SCD1) in isolated hepatocytes was significantly elevated in KO NAFLD mice compared to WT NAFLD mice; however, the mRNA expression of the lipid oxidation genes carnitine palmitoyltransferase 1a (CPT1a), acyl-coenzyme A oxidase 1 (ACOX1), and peroxisome proliferator-activated receptor alpha (PPARα) was not significantly different between KO NAFLD mice and WT NAFLD mice (Fig. 1D). In addition, obvious liver dysfunction, including higher ALT and AST levels, was observed in MYDGF-deficient NAFLD mice (Fig. 1E, *P* < 0.001). As shown by these data, MYDGF deficiency in myeloid cells exacerbated lipogenesis and steatosis in NAFLD mice.

We next explored the effects of MYDGF deficiency in myeloid cells on inflammation in mice. The results of immunohistochemical (IHC) analyses showed that the proportion of hepatic macrophages was significantly increased in HFD-fed MYDGF-deficient mice compared to WT mice (Fig. 1F, *P* < 0.0001). Moreover, the levels of inflammatory factors in isolated hepatocytes, Kupffer cells (KCs), and serum (Fig. 1G–I, *P* < 0.001) were significantly increased in HFD-fed KO mice compared to WT mice. Collectively, these data demonstrate that myeloid cell-specific MYDGF deficiency aggravated inflammation in NAFLD mice.

In accordance with our previous study [11], the data also indicated that, compared to WT mice, KO mice showed decreased glucose and insulin tolerance (Fig. S3A–D, Table S4, *P* < 0.001), developed more severe dyslipidemia (including higher TG, TC, and FFA levels) (Table S4, *P* < 0.01), and exhibited increased body weight gain (Fig. S3E, F, *P* < 0.001), but showed no significant differences in food intake, fecal output, fecal lipid content, or blood pressure (Table S4, *P* > 0.05). These data reveal that myeloid cell-specific MYDGF deficiency impaired lipid metabolism.

### Bone marrow transplantation alleviated inflammation, lipogenesis, and hepatic steatosis in NAFLD mice

**Fig. 2:**
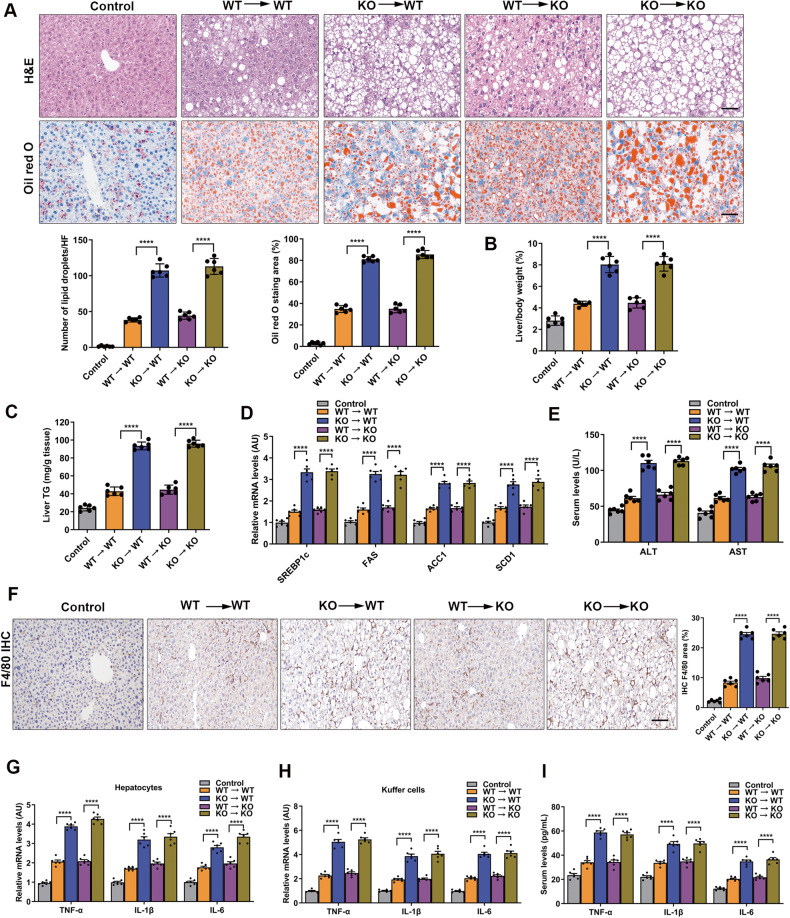
BMT alleviated inflammation, lipogenesis, and hepatic steatosis in NAFLD mice. BMT was performed in KO and WT mice aged 4–6 weeks, which were fed a HFD for 12 weeks as indicated in Fig. S4C. **A** Representative images showing HE staining and oil red O staining of liver sections (*n* = 6). **B** Liver/body weight (*n* = 6). **C** Hepatic TG content (*n* = 6). **D** mRNA levels of genes related to fat metabolism in isolated hepatocytes (*n* = 6). **E** Serum ALT and AST levels (*n* = 6). **F** Representative images showing F4/80 staining of liver sections (*n* = 6). **G** Levels of inflammatory factors in isolated hepatocytes (*n* = 6). **H** Levels of inflammatory factors in KCs (*n* = 6). **I** Levels of inflammatory factors in serum (*n* = 6). Data are presented as the mean ± SEM. Black scale bar: 50 μm. ^*^P < 0.05, ^**^P < 0.01, ^***^P < 0.001, ^****^P < 0.0001. Significant differences were determined by Student’s *t*-test or one-way ANOVA followed by Tukey’s post-test.

We were next interested in assessing the extent of hepatic inflammation, lipogenesis, and steatosis after myeloid cell MYDGF restoration in KO mice. Therefore, we generated bone marrow chimeric mice to specifically address the protection of MYDGF restoration on inflammation and NAFLD. First, we confirmed the efficiency of bone marrow transplantation (BMT). Four weeks after KO recipient mice were transplanted with bone marrow cells (BMCs) from a WT donor mouse, MYDGF began to be expressed in the circulation and was sustained at a high level (Fig. S4A, *P* < 0.001). Similarly, serum MYDGF levels were higher in WT recipient mice transplanted with WT BMCs than in WT recipient mice transplanted with KO BMCs (Fig. S4B, *P* < 0.001). Next, we detected the protective role of BMT in NAFLD mice using a series of transplantation experiments (Fig. S4C). Transplantation of WT BMCs into KO NAFLD recipients decreased the liver index, alleviated lipogenesis and hepatic steatosis, reduced macrophage infiltration, decreased the levels of inflammatory factors in isolated hepatocytes, KCs and serum (Fig. 2A–I, *P* < 0.001), and improved the metabolic profiles (Fig. S5, Table S5, *P* < 0.001). In contrast, transplantation of KO BMCs into WT recipients worsened NAFLD (Fig. 2A–I) and metabolic profiles (Fig. S5, Table S5). These results reveal that bone marrow cell-derived MYDGF was both sufficient and necessary to regulate hepatic inflammation, lipogenesis, hepatic steatosis, and metabolic disorders in NAFLD mice.

### Bone marrow-specific overexpression of MYDGF improved inflammation, lipogenesis, and hepatic steatosis in NAFLD mice

**Fig. 3:**
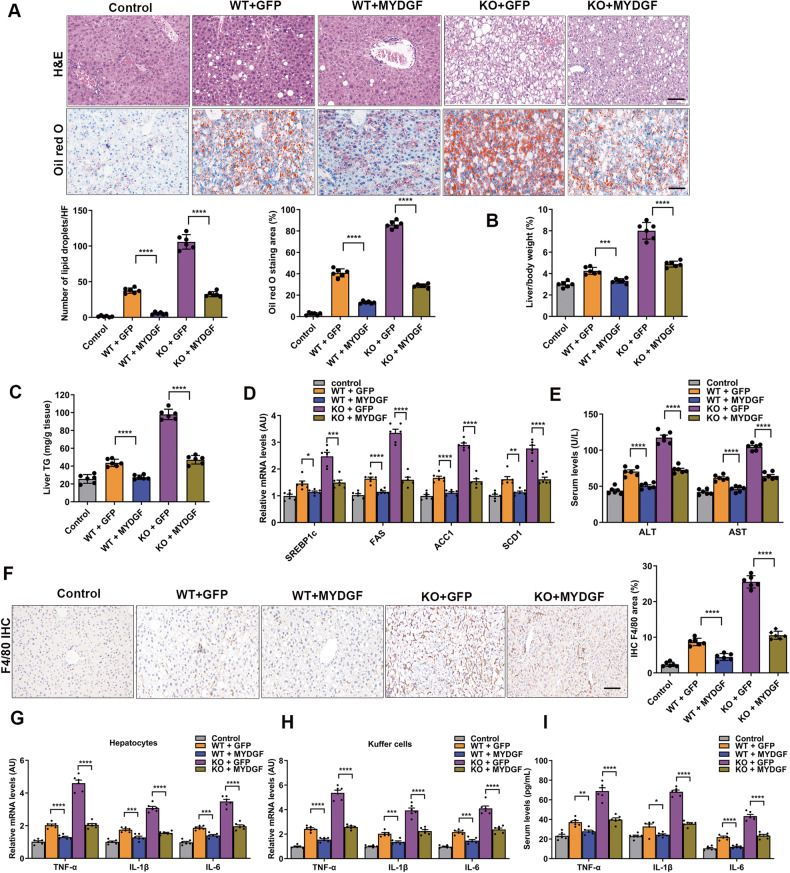
Bone marrow-specific overexpression of MYDGF alleviated inflammation, lipogenesis, and hepatic steatosis in NAFLD mice. Intramarrow AAV-MYDGF injection was performed in KO and WT mice aged 4–6 weeks, which were fed a HFD for 12 weeks as indicated in Fig. S6E. **A** Representative images showing HE and oil red O staining of liver sections (*n* = 6). **B** Liver/body weight (*n* = 6). **C** Hepatic TG content (*n* = 6). **D** mRNA levels of genes related to fat metabolism in isolated hepatocytes (*n* = 6). **E** Serum ALT and AST levels (*n* = 6). **F** Representative images showing F4/80 staining of liver sections (*n* = 6). **G** Levels of inflammatory factors in isolated hepatocytes (*n* = 6). **H** Levels of inflammatory factors in KCs (*n* = 6). **I** Levels of inflammatory factors in serum (*n* = 6). Data are presented as the mean ± SEM. Black scale bar: 50 μm. ^*^P < 0.05, ^**^P < 0.01, ^***^P < 0.001, ^****^P < 0.0001. Significant differences were determined by Student’s *t*-test or one-way ANOVA followed by Tukey’s post-test.

To further verify that bone marrow-derived MYDGF restoration improved hepatic inflammation, lipogenesis, and steatosis in KO mice, we injected adeno-associated viral (AAV)-MYDGF and AAV-GFP at a dose of 1 × 10^12^ VP into the marrow cavities of NAFLD mice, and then assessed inflammation, lipogenesis, hepatic steatosis, and metabolism. We first determined the efficiency of MYDGF expression following intramarrow injection. Interestingly, MYDGF expression could be detected in serum 7 days after AAV-MYDGF transfer and was detected for up to 3 weeks in mice following a single intramarrow injection (Fig. S6A, *P* < 0.001). To obtain the continuous expression of MYDGF in both the marrow cavity and serum, KO mice received intramarrow injection of AAV-MYDGF every 3 weeks for 12 weeks, and the results showed that serum MYDGF expression was maintained at high levels (Fig. S6B, *P* < 0.001). In parallel, the MYDGF protein levels and the fluorescence expression of MYDGF in the marrow of AAV-MYDGF-treated mice were significantly higher than those in AAV-GFP-treated mice at 3 weeks, while a low abundance of MYDGF expression was found in the liver, myocardium, lung, and kidney in KO mice (Fig. S6C, D, *P* < 0.001), indicating bone marrow-specific overexpression of MYDGF. The results of the experiments shown in Fig. S6E demonstrated that intramarrow injection of AAV-MYDGF decreased the liver index, alleviated hepatic steatosis, decreased liver TG levels, decreased lipogenesis, reduced inflammation (Fig. 3A–I), and improved metabolic profiles (Fig. S7, Table S6), especially in KO mice. Regarding inflammation, we found that the proportions of macrophages in both WT and KO NAFLD mice were significantly lower after MYDGF replenishment (Fig. 3F, *P* < 0.001). Further analysis of macrophage polarization demonstrated that MYDGF restoration decreased macrophage inflammatory activation, as evidenced by the decreased polarization of M1 macrophages and increased polarization of M2 macrophages (Fig. S8A, B, *P* < 0.001). These data illustrate that MYDGF restoration improved inflammation, lipogenesis, and hepatic steatosis in NAFLD mice.

### MYDGF improved inflammation, lipogenesis, and fat deposition in primary mouse hepatocytes

**Fig. 4:**
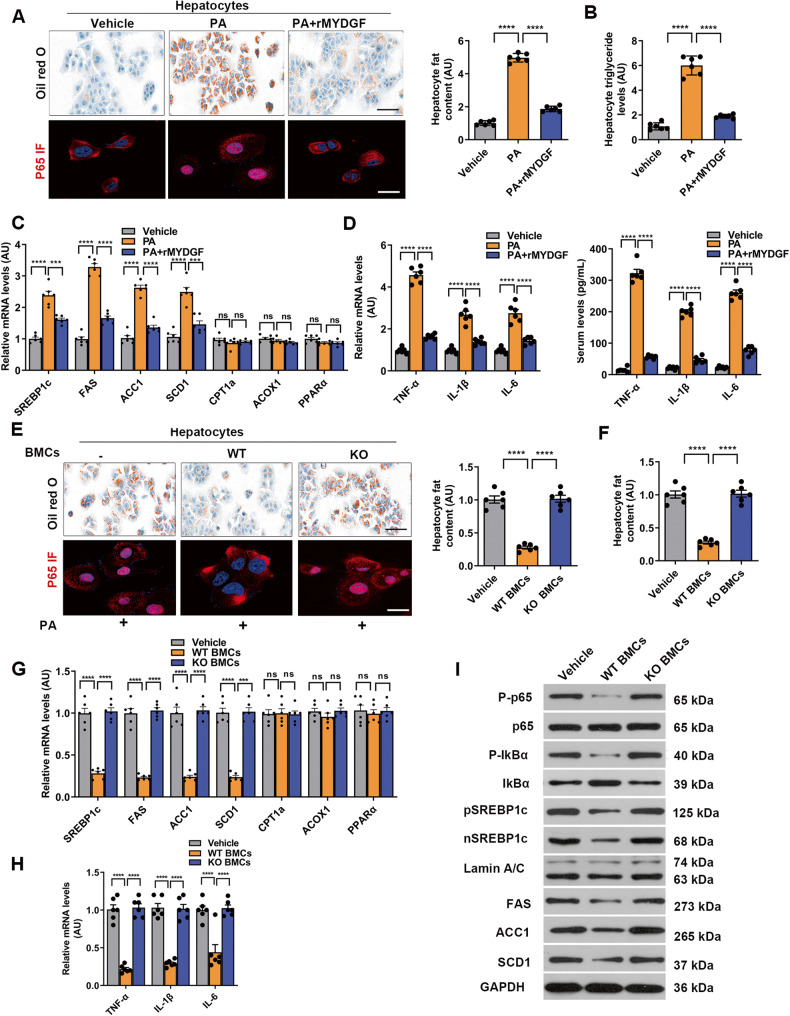
MYDGF improved hepatocyte inflammation, lipogenesis, and fat deposition in co-culture experiments. The experiments were performed as indicated in Fig. S9C, D. **A** Fat content (determined by oil red O staining) and p65 nuclear translocation in rMYDGF-treated PMHs. **B** Quantification of TG levels in rMYDGF-treated PMHs. **C** mRNA levels of genes related to fat metabolism in rMYDGF-treated PMHs. **D** Levels of inflammatory factors in hepatocytes and supernatants after rMYDGF treatment. **E** Fat content (determined by oil red O staining) and p65 nuclear translocation in PMHs co-cultured with BMCs. **F** Quantification of TG levels in PMHs co-cultured with BMCs. **G** mRNA levels of genes related to fat metabolism in PMHs co-cultured with BMCs. **H** mRNA levels of TNFα, IL-1β, and IL-6 in PMHs co-cultured with BMCs. **I** Levels of P-p65, p65, P-IκBα, IκBα, pSREBP1c, nSREBP1c, FAS, ACC1, and SCD1 proteins in PMHs co-cultured with BMCs. Black scale bar: 20 μm, white scale bar: 10 μm. Each experiment was repeated five times. Data are presented as the mean ± SEM. ^*^P < 0.05, ^**^P < 0.01, ^***^P < 0.001, ^****^P < 0.0001. Significant differences were determined by Student’s *t*-test or one-way ANOVA followed by Tukey’s post-test.

We next questioned whether MYDGF has a direct effect on primary mouse hepatocytes (PMHs). We initially explored time- and dose-dependent palmitate (PA)-induced changes in fat deposition. We selected treatment with 0.4 mmol/L PA for 24 h and 50 ng/mL recombinant MYDGF (rMYDGF) for 24 h as the optimum conditions (Fig. S9A, B, *P* < 0.001). We next conducted a formal study, the plan of which is shown in Fig. 9SC. Compared to the control group, the fat content was significantly increased under PA-treated conditions, as detected by oil red O staining, and this effect was partially blocked by treatment with rMYDGF (Fig. 4A, *P* < 0.001). Consistent with these findings, rMYDGF significantly decreased TG levels (Fig. 4B, P < 0.001) and reduced the levels of lipid synthesis genes in PA-induced PMHs (Fig. 4C, *P* < 0.001). Notably, after 24-h PA stimulation, rMYDGF significantly attenuated PA-induced p65 nuclear translocation (Fig. 4A, *P* < 0.001) and inflammation (Fig. 4D, P < 0.001). Therefore, these results indicate that rMYDGF directly protects PMHs from PA-induced changes in inflammation, lipogenesis, and fat deposition.

### MYDGF attenuated PA-induced hepatocyte inflammation, lipogenesis, and fat deposition in co-culture experiments

To further confirm that myeloid cell-derived MYDGF is a factor involved in the crosstalk between bone marrow and liver in vitro, we performed the following co-culture experiments with BMCs and PMHs treated with PA (0.4 mmol/L) (Fig. S9D). Initially, we co-cultured PMHs with WT or KO BMCs for 24 h. Co-culture with WT BMCs decreased the accumulation of fat and TG levels in PMHs, but co-culture with KO BMCs did not (Fig. 4E, F, *P* < 0.0001). Importantly, co-culture with WT BMCs reduced the mRNA expression of lipid synthesis genes in PA-induced hepatocytes (Fig. 4G, *P* < 0.0001), but not in PMHs co-cultured with KO BMCs (Fig. 4G, *P* > 0.05).

Analysis of inflammatory parameters showed that co-culture with WT BMCs obviously decreased mRNA levels of inflammatory factors in hepatocytes (Fig. 4H, *P* < 0.0001) as well as p65 nuclear translocation (Fig. 4E), while co-culture with KO BMCs did not. Accordingly, co-culture with WT BMCs attenuated expression of phosphorylated I-kappa-B-alpha (P-IkBα), nuclear P-p65, FAS, ACC1, SCD1, precursor SREBP1c (pSREBP1c), and nuclear SREBP1c (nSREBP1c) proteins induced by PA in hepatocytes but not in hepatocytes co-cultured with KO BMCs (Fig. 4I). Taken together, these results further support our hypothesis that MYDGF serves as a factor involved in the crosstalk between the bone marrow and liver and that MYDGF derived from BMCs protects against NAFLD by decreasing hepatocyte inflammation and lipogenesis.

### MYDGF improved inflammation and promoted the polarization of macrophages toward M2 in KCs

We next questioned whether MYDGF has a direct effect on KCs (Fig. S10A). Notably, rMYDGF significantly attenuated PA-induced inflammation (Fig. S10B, C, *P* < 0.0001) and promoted the polarization of macrophages toward M2 (Fig. S10D, *P* < 0.0001**)** in KCs. In addition, we performed the following co-culture experiments with BMCs and KCs (Fig. S10E). Co-culture with WT BMCs obviously decreased the mRNA levels of inflammatory factors in KCs (Fig. S10F, *P* < 0.0001) and promoted the polarization of macrophages toward M2 (Fig. S10G, *P* < 0.001), while co-culture with KO BMCs did not. Taken together, these results support that MYDGF derived from myeloid cells protects against NAFLD by decreasing inflammation levels in KCs.

### IKKβ/NF-κB signaling pathway is involved in the beneficial effects of MYDGF in NAFLD in vivo and in vitro

**Fig. 5:**
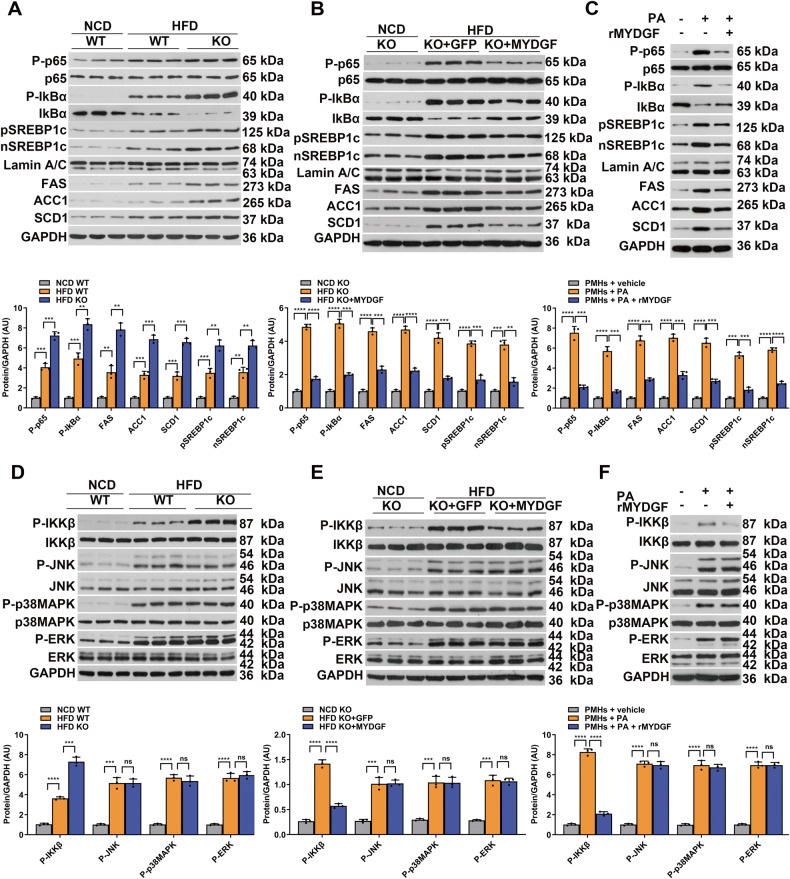
MYDGF inhibited NF-κB signaling and lipogenesis in vivo and in vitro. **A** Levels of NF-κB signaling and lipogenesis proteins in the livers of KO mice (*n* = 3). **B** Levels of NF-κB signaling and lipogenesis proteins in AAV-MYDGF-treated livers of KO mice (*n* = 3). **C** Levels of NF-κB signaling and lipogenesis proteins in rMYDGF-treated PMHs. **D** Levels of P-IKKβ, P-JNK, P-p38MAPK, and P-ERK proteins in the livers of KO mice (*n* = 3). **E** Levels of P-IKKβ, P-JNK, P-p38MAPK, and P-ERK proteins in AAV-MYDGF-treated livers of KO mice (*n* = 3). **F** Levels of P-IKKβ, P-JNK, P-p38MAPK, and P-ERK proteins in rMYDGF-treated PMHs. Each in vitro experiment was repeated five times. Data are presented as the mean ± SEM. ^*^P < 0.05, ^**^P < 0.01, ^***^P < 0.001, ^****^P < 0.0001. Significant differences were determined by Student’s *t*-test or one-way ANOVA followed by Tukey’s post-test.

Our in vivo and in vitro data suggest that MYDGF downregulates hepatocyte inflammation and lipogenesis. It has been established that inflammatory signaling is strongly linked with SREBP1c [15]. Moreover, nuclear factor-kappa B (NF-κB) signaling is known to play an important role in inflammation [16]. Thus, NF-κB signaling was tested in vivo and in vitro. The results showed that the levels of P-IkBα, nuclear P-p65, pSREBP1c, nSREBP1c, FAS, ACC1, and SCD1 in the liver were increased in myeloid cell-specific KO NAFLD mice compared to WT NAFLD mice (Fig. 5A, *P* < 0.01), while the expression of these proteins in the liver was significantly reduced in MYDGF-replenished mice compared to AAV-GFP mice (Fig. 5B, *P* < 0.01). In parallel to the in vivo experiment, treatment with rMYDGF significantly inhibited PA-induced changes in NF-κB signaling and lipogenesis protein expression in PMHs (Fig. 5C, *P* < 0.001). Furthermore, rMYDGF significantly blunted the expression of another proinflammatory cytokine, TNF-α (treated overnight with 10 ng/mL), and induced changes in NF-κB signaling and lipogenesis in PMHs (data not shown). These results show that NF-κB signaling is essential for the beneficial effects of MYDGF on liver lipogenesis.

NF-κB is regulated by c-jun N-terminal kinase (JNK), p38-mitogen-activated protein kinases (p38MAPK), extracellular signal-regulated kinase (ERK), and inhibitor kappa B kinase beta (IKK) [17, 18]. Our results suggested that P-IKKβ expression in the liver was increased in KO NAFLD mice compared to WT NAFLD mice (Fig. 5D, *P* < 0.001), but was significantly decreased in MYDGF-replenished mice compared to AAV-GFP mice (Fig. 5E, *P* < 0.001). However, the expression of the other proteins was unaffected by MYDGF. Consistently, treatment with rMYDGF significantly inhibited PA-induced changes in P-IKKβ expression in PMHs but did not significantly alter the expression of P-JNK, P-p38MAPK, or P-ERK (Fig. 5F, *P* < 0.001). These data suggest that IKKβ signaling is involved in the beneficial effects of MYDGF observed in NAFLD mice.

**Fig. 6:**
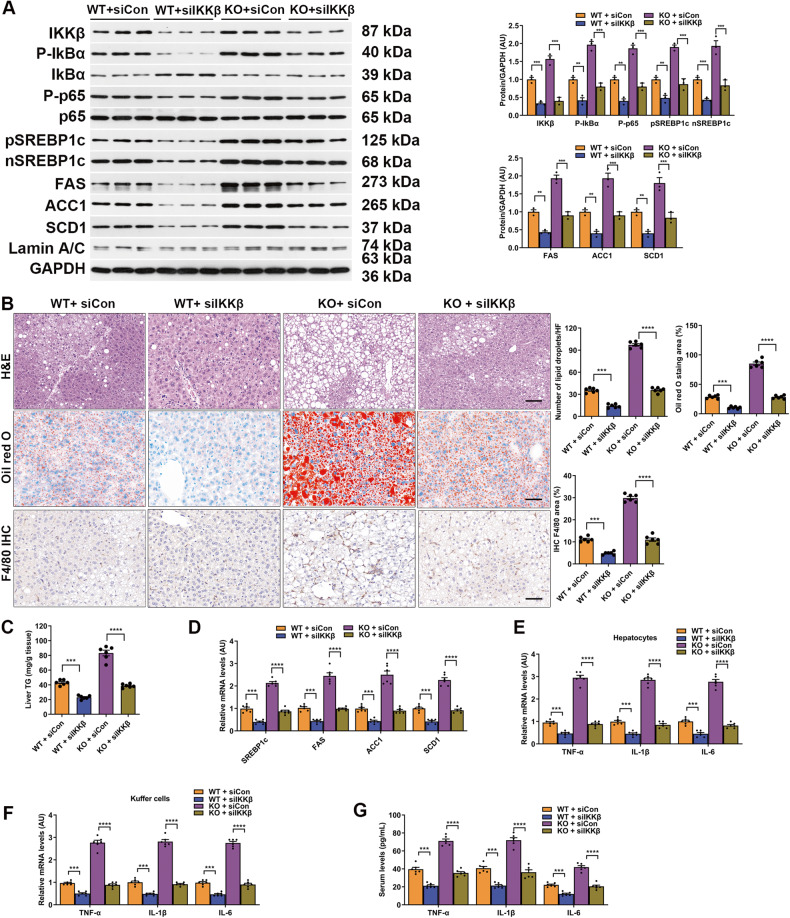
IKKβ silencing inhibited NF-κB signaling and lipogenesis and alleviated inflammation and fat deposition in vivo. **A**–**G** WT and KO mice aged 4–6 weeks were treated with siIKKβ and siCon and fed a HFD for 12 weeks. **A** Levels of NF-κB signaling and lipogenesis proteins in the livers of siIKKβ-transfected WT and KO mice (*n* = 3). **B** Representative images showing HE staining, oil red O staining, and F4/80 staining of liver sections (*n* = 6). Black scale bar: 50 μm. **C** Hepatic TG content (*n* = 6). **D** mRNA levels of genes related to fat metabolism in isolated hepatocytes (*n* = 6). **E** Levels of inflammatory factors in isolated hepatocyte (*n* = 6). **F** Levels of inflammatory factors in KCs (*n* = 6). **G** Levels of inflammatory factors in serum (*n* = 6). Data are presented as the mean ± SEM. ^*^P < 0.05, ^**^P < 0.01, ^***^P < 0.001, ^****^P < 0.0001. Significant differences were determined by Student’s *t*-test or one-way ANOVA followed by Tukey’s post-test.

Next, we sought to further confirm the involvement of the IKKβ pathway in the beneficial effects of MYDGF using RNA interfering mediated gene silencing in vivo and in vitro. For small interfering RNA (siRNA) treatment, myeloid cell-specific KO NAFLD mice received a single injection of liver-specific AAV-siRNA/IKKβ (siIKKβ) or AAV-siRNA/control (siCon) through the tail vein. As expected, after 7 days of intervention, the protein and mRNA expression of IKKβ in the liver was reduced by 76% and 79%, respectively, compared to that in the siCon-transfected group (Fig. S11A, B, *P* < 0.001). The formal experimental results showed that IKKβ silencing decreased the expression of NF-κB signaling proteins in the liver compared to those in both the siCon-transfected WT and KO mice, but to a lesser extent in the KO mice (Fig. 6A, *P* < 0.05). Consequently, inflammation, lipogenesis, and hepatic steatosis were similarly attenuated by siIKKβ intervention, although to a lesser extent in the KO mice (Fig. 6B–G, *P* < 0.01). To further support these findings, we treated KO mice with siIKKβ and AAV-MYDGF and fed them a HFD for 12 weeks. As expected, IKKβ silencing in KO mice decreased the expression of NF-κB signaling and lipogenesis proteins, as well as inflammation, lipogenesis, and fat deposition (Fig. S12A–G). Importantly, the effects of AAV-MYDGF treatment closely mimicked those of siIKKβ interventions (Fig. S12A–G). Furthermore, the beneficial effects of MYDGF treatment and IKKβ knockdown were not additive (Fig. 12SA–G). These data confirm that IKKβ is, at least partially, essential for the beneficial effects of MYDGF on NAFLD mice.

**Fig. 7:**
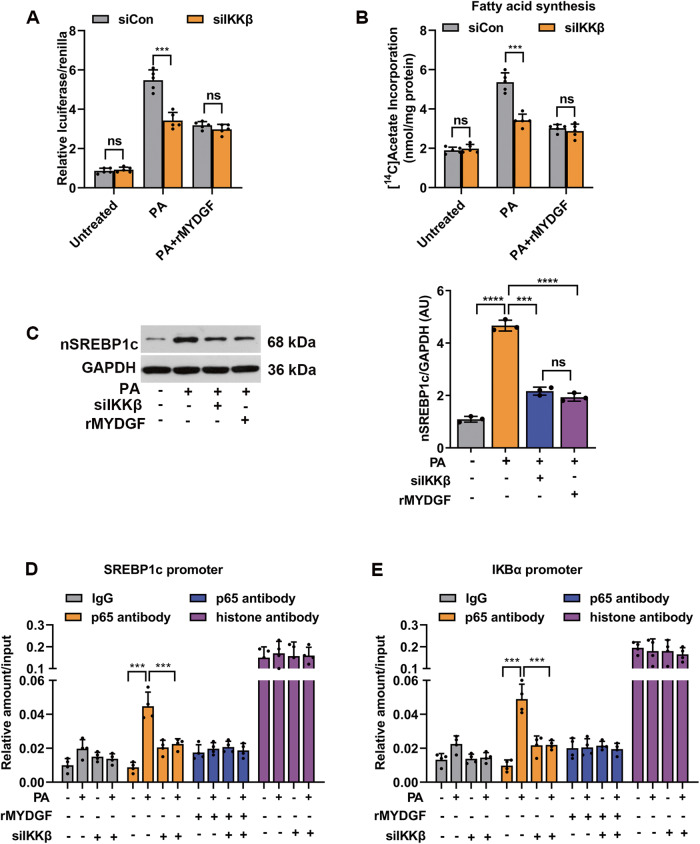
Luciferase and ChIP under IKKβ silencing conditions in PMHs. PMHs were transfected with siIKKβ or siCon for 12 h, before using for luciferase and ChIP experiments. **A** PMHs were transfected with NF-κB-luciferase or SV40–Renilla after treatment with siCon or siIKKβ. Cells were left untreated or treated with rMYDGF for 24 h, and/or PA for 24 h before luciferase and Renilla assessment. **B** PMHs were transfected with siCon or siIKKβ, and then treated with or without rMYDGF for 24 h, and/or PA for 24 h. De novo fatty acid biosynthesis was measured before or after PA stimulation. **C** Nuclear protein was analyzed for SREBP1c by immunoblotting, and GAPDH was used as the normalization control; nSREBP1c denotes nuclear SREBP1c. **D**, **E** PMHs were transfected with siCon and siIKKβ, and then treated with or without rMYDGF for 24 h, and/or PA for 24 h. IgG, p65, and histone antibodies were used as a reference substrate. ChIP and RT-PCR was performed to determine SREBP1c (**D**) and IkBα (**E**) promoters. Data are presented as the mean ± SEM. Each experiment was repeated five times. ^*^P < 0.05, ^**^P < 0.01, ^***^P < 0.001, ^****^P < 0.0001. Significant differences were determined by Student’s *t*-test or one-way ANOVA followed by Tukey’s post-test.

In parallel to the in vivo experimental results, after 48 h of intervention, siIKKβ effectively silenced IKKβ gene expression in PMHs (Fig. S11C, D, *P* < 0.001). IKKβ silencing in hepatocytes decreased the expression of NF-κB signaling and lipogenesis proteins induced by PA compared to siCon-transfected cells (Fig. S13A, *P* < 0.001). Consistently, treatment with siIKKβ significantly inhibited PA-induced p65 nuclear translocation in PMHs (Fig. S13B, *P* < 0.001). Subsequently, inflammation, lipogenesis, and fat deposition were attenuated by siIKKβ intervention in hepatocytes under PA-stimulated conditions (Fig. S13B–E). We next aimed to establish the role of rMYDGF in protecting against fat deposition in IKKβ-silenced hepatocytes. The results showed that the effects of rMYDGF treatment closely mimicked those of siIKKβ interventions, as evidenced by the significantly decreased expression of NF-κB signaling and lipogenesis proteins, and decreased PA-induced p65 nuclear translocation, fat content, TG levels, lipogenesis, and mRNA levels of inflammatory markers (Fig. S13A–E) in PA-stimulated hepatocytes. Furthermore, co-treatment with rMYDGF and siIKKβ produced similar effects (Fig. S13A–E). As it is well known that the IKKβ pro-inflammatory pathway disrupts insulin signaling [19], we next investigated the role of rMYDGF in PA-induced attenuation of insulin signaling. As expected, insulin-stimulated insulin receptor phosphorylation and Akt phosphorylation (S473) were significantly increased both in rMYDGF treated and IKKβ-silenced PA-stimulated PMHs (Fig. S14A, B). Taken together, these findings illustrate that IKKβ/NF-κB signaling is essential for MYDGF-mediated protection against NAFLD. In addition, we sought to address how NF-κB modulates MYDGF-mediated SREBP1c transcriptional activity, initially, by using a luciferase reporter containing NF-κB-binding elements in siIKKβ-silenced PMHs. The results showed that PA-induced NF-κB transcriptional activity was reduced when IKKβ was silenced, and rMYDGF mimicked the effects of siIKKβ on NF-κB transcriptional activity (Fig. 7A, *P* < 0.001). We also showed that de novo lipogenesis in PMHs was increased by PA, but reversed by siIKKβ or rMYDGF (Fig. 7B, *P* < 0.001). PA also stimulated the accumulation of SREBP1c nuclear protein, which was reversed by siIKKβ or rMYDGF (Fig. 7C, *P* < 0.001). Previous studies have reported that NF-κB can regulate SREBP1c via binding sites on the promoter of SREBP1c [20–22]. Using p65 chromatin immunoprecipitation (ChIP), the increased p65 binding to the SREBP1c and IkBα promoters induced by PA was decreased when IKKβ was silenced in PMHs, while rMYDGF mimicked the roles of siIKKβ on p65 binding (Fig. 7D, E), indicating that MYDGF inhibits NF-κB transcriptional and binding activity to SREBP1c through IKKβ.

**Fig. 8:**
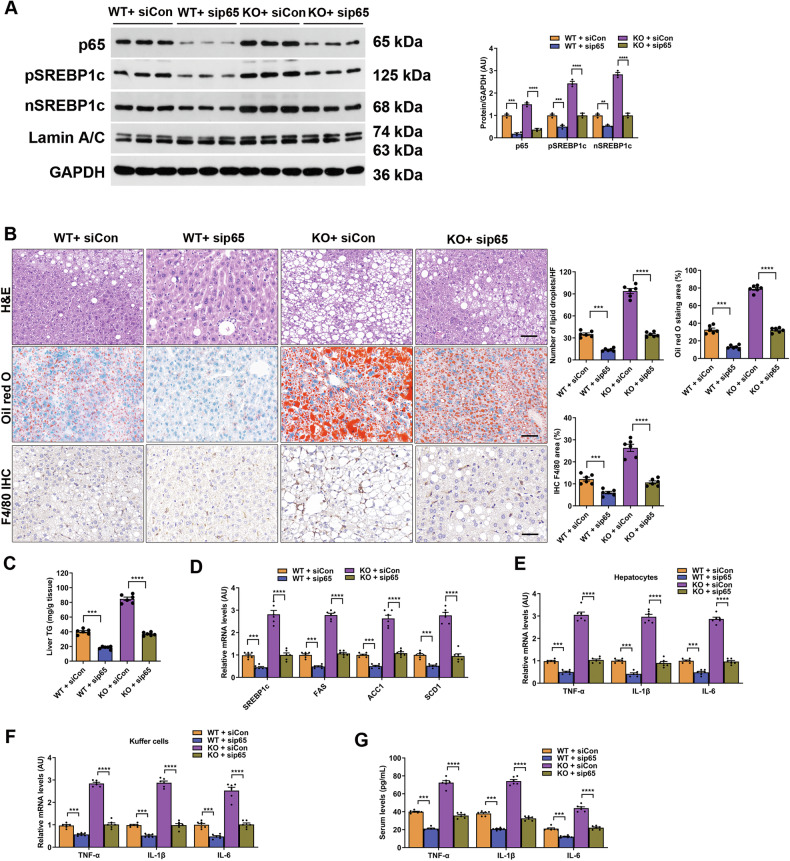
p65 silencing inhibited the expression of SREBP1c and alleviated inflammation and fat deposition in vivo. **A**–**G** WT and KO mice aged 4–6 weeks were treated with sip65 or siCon and fed a HFD for 12 weeks. **A** Expression of SREBP1c proteins in the livers of sip65-transfected WT and KO mice (*n* = 3). **B** Representative images showing HE staining, oil red O staining, and F4/80 staining of liver sections (*n* = 6). Black scale bar: 50 μm. **C** Hepatic TG content (*n* = 6). **D** mRNA levels of genes related to fat metabolism in isolated hepatocytes (*n* = 6). **E** Levels of inflammatory factors in isolated hepatocytes (*n* = 6). **F** Levels of inflammatory factors in KCs (*n* = 6). **G** Levels of inflammatory factors in serum (*n* = 6). Data are presented as the mean ± SEM. ^*^P < 0.05, ^**^P < 0.01, ^***^P < 0.001, ^****^P < 0.0001. Significant differences were determined by Student’s *t*-test or one-way ANOVA followed by Tukey’s post-test.

We next employed RNAi-mediated gene silencing in vivo to confirm the involvement of the NF-κB/SREBP1c pathway in the beneficial effects of MYDGF. The results showed that p65 silencing decreased the expression of SREBP1c proteins in the liver compared to those in siCon-transfected WT and KO mice, but to a lesser extent in KO mice (Fig. 8A, *P* < 0.01). Consequently, inflammation, lipogenesis, and hepatic steatosis were attenuated by p65 silencing, especially in WT mice (Fig. 8B–G, *P* < 0.01). These data confirm that p65 is essential for the beneficial effects of MYDGF on NAFLD mice.

**Fig. 9:**
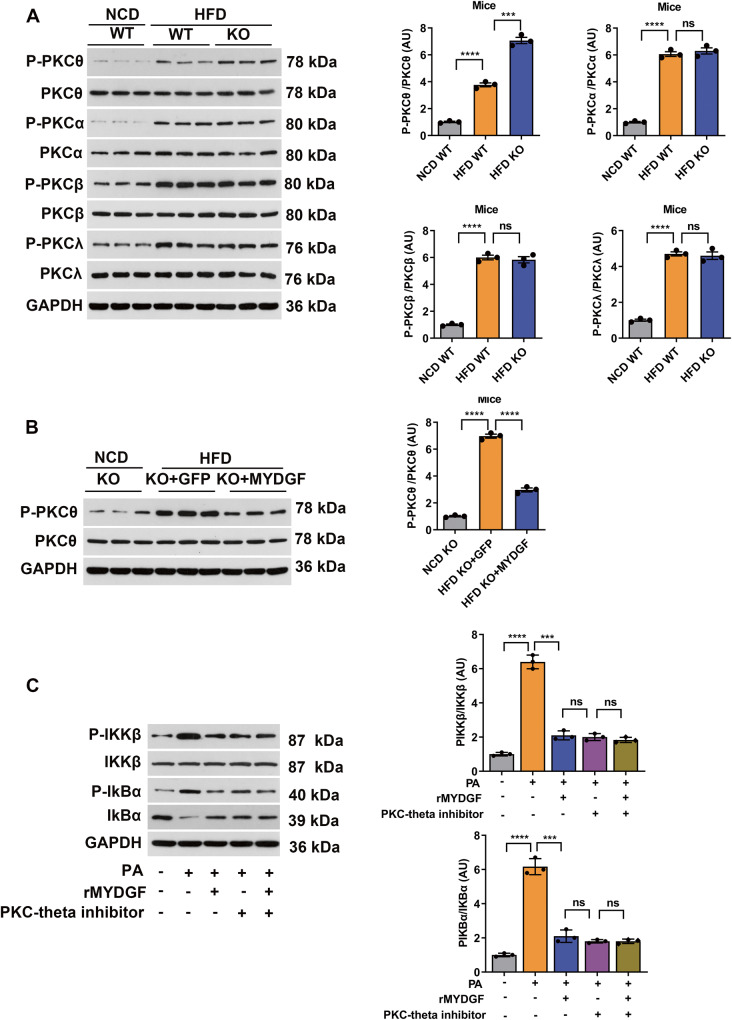
PKCθ is involved in the MYDGF-induced regulation of IKKβ phosphorylation in hepatocytes. **A** Levels of PKC signaling proteins in the livers of KO mice (*n* = 3). **B** Levels of PKC signaling proteins in the livers of AAV-MYDGF-treated KO mice (*n* = 3). **C** Levels of PKC signaling proteins in rMYDGF-treated or PKCθ inhibitor-treated PMHs. ^*^P < 0.05, ^**^P < 0.01, ^***^P < 0.001, ^****^P < 0.0001. Significant differences were determined by Student’s *t*-test or one-way ANOVA followed by Tukey’s post-test.

**Fig. 10:**
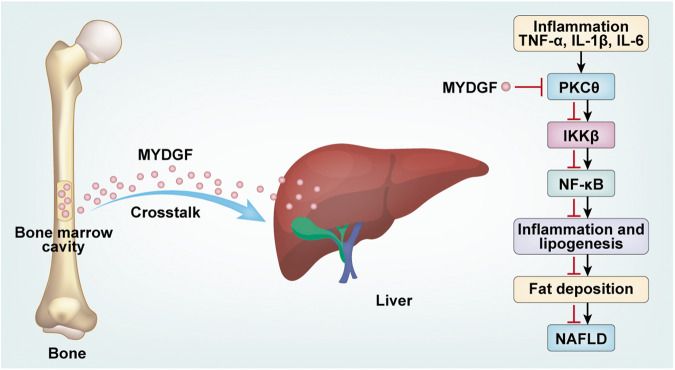
Schematic showing that MYDGF plays a protective role in NAFLD via the IKKβ/NF-κB signaling pathway.

In the final experiments, we sought to explore how MYDGF regulates the phosphorylation of IKKβ in hepatocytes. As IKKβ is usually regulated by protein kinase C (PKC) [23, 24], we determined the expression of the PKC isoforms α, β, θ, and λ. The results showed that the levels of P-PKCθ in the liver were increased in KO NAFLD mice compared to those in WT NAFLD mice (Fig. 9A, B, *P* < 0.001), while the expression of P-PKCθ in the liver was significantly decreased in MYDGF-replenished mice compared to AAV-GFP mice (Fig. 9A, B, *P* < 0.001). However, P-PKCα, P-PKCβ, and P-PKCλ expression was unaffected by MYDGF (Fig. 9A, B, P > 0.05). Besides, rMYDGF treatment in hepatocytes decreased the PA-induced expression of P-IKKβ and P-IKBα (Fig. 9C, *P* < 0.001). In addition, to further verify that PKCθ is involved in the upstream events of IKKβ signaling, we treated PMHs with a PKCθ inhibitor. The results showed that the effects of treatment with 2 μmol/L PKCθ inhibitor for 24 h strongly mimicked those of rMYDGF intervention, as evidenced by the significantly decreased expression of P-IKKβ and P-IKBα (Fig. 9C, *P* < 0.001). These data suggest that PKCθ is involved in the MYDGF-mediated regulation of IKKβ phosphorylation in hepatocytes (Fig. 10).

## Discussion

The main findings of the present study are as follows: (1) Myeloid cell-derived MYDGF inhibited inflammation and attenuated hepatic de novo lipid synthesis to protect against NAFLD; (2) myeloid cell-derived MYDGF serves as a molecular messenger that mediates crosstalk between the bone marrow and liver to regulate liver fat metabolism; (3) MYDGF inhibits the inflammation of KCs; and (4) the IKKβ/NF-κB signaling pathway is involved in the beneficial effects of MYDGF on NAFLD. To the best of our knowledge, this is the first time that myeloid cell-derived MYDGF has been found to protect against NAFLD, and that the bone marrow, which functions as an endocrine organ, has been shown to regulate liver fat metabolism via MYDGF.

NAFLD is characterized by disordered lipid metabolism with complicated manifestations, including inflammation, lipogenesis, hepatic steatosis, and fibrosis [25, 26]. Here, patients and mice with NAFLD exhibited decreased circulating MYDGF levels and increased inflammation, metabolic disorders, and liver dysfunction. Importantly, myeloid cell-derived MYDGF deficiency aggravated the liver index, lipogenesis, liver dysfunction, and lipid deposition, while restoration of myeloid cell-derived MYDGF reversed these changes. Furthermore, treatment of PMHs with rMYDGF decreased the fat content and TG levels. These results reveal the protective role of myeloid cell-derived MYDGF in NAFLD by inhibiting lipogenesis.

Many studies have revealed that inflammation promotes liver lipogenesis and triggers the development and progression of NAFLD [27, 28]. Here, increased inflammation was found in both patients and mice with NAFLD. The results of our animal experiments showed that MYDGF alleviated inflammation in NAFLD. In parallel, treatment of PMHs with rMYDGF significantly attenuated inflammation and decreased p65 nuclear translocation. Inflammation can directly affect hepatocytes to increase lipogenic events and trigger and exacerbate steatosis [29, 30]. SREBP1c is a nuclear transcription factor responsible for the upregulation of hepatic lipogenic genes [31]. Indeed, nSREBP1c expression was found to be increased in MYDGF-deficient NAFLD mice, and lipogenic enzyme expression was significantly increased, both of which were reversed by MYDGF restoration. In addition, MYDGF treatment reduced the expression of nSREBP1c and lipogenic enzymes. These data show that MYDGF inhibited inflammation and de novo lipid synthesis, and alleviated NAFLD. In contrast, the expression of lipid oxidation genes was unaltered by MYDGF intervention, indicating that lipid lipolysis is not involved in the beneficial effects of MYDGF observed in NAFLD.

Considering that macrophages have been implicated to critically determine the development and progression of obesity-associated NAFLD, it is necessary to address how MYDGF in macrophages alters certain aspects of NAFLD. In our study, we confirmed that myeloid cell-derived MYDGF deficiency exacerbates liver macrophage proinflammatory activation. Further, we found that MYDGF has a direct effect on KCs, as evidenced by the attenuation of PA-induced inflammation and promotion of macrophage M2 polarization, demonstrating that MYDGF derived from myeloid cells protects against NAFLD partly by decreasing inflammation levels in KCs.

In accordance with the results of our previous study [11], we also found that MYDGF can improve glucose-lipid metabolism and IR in NAFLD, and that these positive effects can contribute to the protective effects of MYDGF in the context of NAFLD.

We are also interested in the MYDGF signaling mechanisms responsible for its protective effects against NAFLD. NF-κB plays a critical role in the regulation of inflammation, and the NF-κB protein is normally anchored in the cytoplasm by the inhibitory protein IκBα [32, 33]. Here, we demonstrated that inflammation activated the NF-κB signaling pathway and that MYDGF attenuated this activation, as evidenced by the decreased expression of P-IκBα and nuclear P-p65, as well as the nuclear translocation of p65. Furthermore, IKKβ, p38MAPK, ERK, and JNK have been reported as upstream molecules of the NF-κB signaling pathway [34, 35]. Our animal experiments showed that IKKβ is involved in the effect of MYDGF on the NF-κB signaling pathway, but that p38MAPK, ERK, and JNK are not involved, all of which were confirmed by the results of our in vitro experiments. Importantly, when IKKβ in the livers of NAFLD mice was silenced by siRNA, NF-κB signaling was not activated, and inflammation and liver fat metabolism were subsequently improved, verifying that NF-κB signaling is a downstream signal of IKKβ. Besides, p65 silencing in the liver decreased the expression of SREBP1c protein and attenuated NAFLD. Moreover, we found that PKCθ is involved in the MYDGF-mediated phosphorylation of IKKβ in hepatocytes; however, the underlying mechanisms by which MYDGF modulates PKCθ are unclear and require further study. Lastly, NF-κB transcriptional activity and p65 binding to the SREBP1c promoter induced by PA were blunted by IKKβ silencing or rMYDGF treatment in PMHs. Taken together, these data suggest that the IKKβ/NF-κB signaling is essential for the inhibition of MYDGF in hepatic de novo lipid synthesis of NAFLD.

In conclusion, we have successfully demonstrated that MYDGF alleviates inflammation, lipogenesis, and hepatic steatosis in a manner involving IKKβ/NF-κB signaling. Consequently, we suggest that bone marrow, an endocrine organ, can regulate liver fat metabolism through MYDGF. Thus, MYDGF may be a therapeutic drug for NAFLD, and bone marrow may serve as a therapeutic target for metabolic disorders such as NAFLD, obesity, and diabetes.

## Materials and methods

### Human study

#### Clinical samples

As it has been established that estrogens affect liver metabolism [36], only male mice and Chinese male NAFLD subjects were selected to exclude the effect of estrogen on lipid metabolism,. From July 2018 to May 2020, a total of 60 newly diagnosed Chinese male NAFLD subjects (aged 30–65 years, mean 47.75 ± 7.26) from the Wuhan area were included. (1) The inclusion criteria were as follows: Males with newly diagnosed NAFLD; > 18 years; Chinese Han people from the Wuhan area; and BMI, 18.5–30 kg/m^2^. (2) The exclusion criteria were as follows: Diabetes, pre-diabetes, thyroid disease, secondary causes of steatohepatitis, other causes of liver disease, including drug-induced steatosis, hepatitis virus infection, autoimmune hepatitis, and chistosomiasis liver disease; heart failure, kidney failure, malignant tumors, other endocrinological disease; and taking any drugs, smokers, or alcohol drinkers. (3) The diagnosis criteria were as follows: NAFLD was defined according to the ADA criteria published in 2018 [37]. The ultrasound grading standard for NAFLD was as follows [38]: Mild NAFLD was defined as enhanced liver echo, unclear posterior echo attenuation, and normal structure of the intrahepatic duct; moderate NAFLD was defined as obviously enhanced liver echo, attenuated posterior echo, and unclear structure of the intrahepatic duct is not clear; and severe NAFLD was defined as obvious posterior echo, unclear rear liver tissue, and unclear diaphragm [39]. Cigarette smokers were defined as subjects who had smoked at least one cigarette daily for 1 year. Alcohol drinkers were defined as men who had current or in the past 6 months intake of alcohol ≥140 g/week. During the same period, 60 healthy male subjects (aged 30–65 years, mean 48.2 ± 6.65 years) were selected as control subjects. All subjects signed informed consent. The study protocol was in agreement with the guidelines of the ethics committee of our hospital and was approved by the ethics committee of the General Hospital of Central Theater Command (Wuhan, China).

#### Human biochemical measurements

Blood samples were obtained from participants after a 12-h fast. Serum samples were stored at –80°C until required for further analysis. Levels of serum TC, TG, LDL-C, high-density lipoprotein cholesterol (HDL-C), blood glycosylated hemoglobin (HbA1c), AST, ALT, and creatinine were determined by colorimetric assays using commercially available kits (Jiancheng Bioengineering Institute, Nanjing, China). Blood glucose (BG), including fasting blood glucose (FBG) and postprandial 2 h blood glucose (after 75-g glucose loading, 2 h BG), was measured by a glucose oxidase procedure. The serum fasting insulin concentration was measured by electrochemiluminescence immunoassay. Serum FFA was determined using an enzymatic colorimetric assay kit from Roche Diagnostics (Mannheim, Germany) according to the manufacturer’s instructions. HOMA-IR was calculated by fasting serum insulin (mU/L) × FBG (mmol/L) / 22.5. Serum MYDGF was measured enzymatically using a commercially available kit (Sino Best Biological, Shanghai, China). Serum levels of IL-6, IL-1β, and TNF-α were measured using an enzyme-linked immunosorbent assay (ELISA) kits (R&D Systems, Inc., Minneapolis, USA). The coefficients of variation for these assays were 1%–2% (FBG, 2 h BG, creatinine, TC, LDL-C, IL-1β, and HDL-C), 2%–3% (MYDGF, TG, insulin, and FFA), and 2%–4% (ALT, AST, HbA1c, IL-6, and TNF-α).

### Animal experiments

C57BL/6 J and MYDGF-floxed (exon 1–3)(MYDGF^F/F^) mice were obtained from Shanghai Model Organisms Center, Inc (Shanghai, China). LysMCre^+^ mice, in which the LysMcre knock-in/knock-out allele has a nuclear-localized Cre recombinase inserted into the first coding ATG of the lysozyme 2 gene (Lyz2), both abolishing endogenous Lyz2 gene function and placing NLS-Cre expression under the control of the endogenous Lyz2 promoter/enhancer elements, were obtained from Jackson Laboratory (Bar Harbor, ME, USA). MYDGF-floxed mice were bred with LysMCre^+^ mice to generate myeloid cell-specific KO and WT mice (Fig. S2A). All mice were housed in cages at a controlled temperature (22 ± 1 °C) and a relative humidity of 50% with a 12:12-h light-dark cycle [40]. A HFD (D12492, Research Diets, Inc.), consisting of 20% calories from proteins, 60% calories from fats, and 20% calories from carbohydrates, was adopted to induce NAFLD in male mice at 4–6 weeks of age, as previously described [41]. All animal procedures were approved by the Animal Care and Use Committee of the General Hospital of Central Theater Command. The animal experiments comprised six parts, described as follows:

First, to explore MYDGF expression in HFD-induced NAFLD mice, C57BL/6 J male mice aged 4–6 weeks were randomized to NAFLD and control groups. The NAFLD group was fed a HFD for 12 weeks, and the control group was fed a normal chow diet (NCD) for 12 weeks (*n* = 10 mice per group).

Second, to explore the effect of myeloid cell-specific MYDGF deficiency on NAFLD mice, WT and KO male mice aged 4–6 weeks were fed a HFD for 12 weeks. (*n* = 8 mice per group).

Third, to explore the effect of bone marrow cell-derived MYDGF on NAFLD mice, WT and KO mice aged 4–6 weeks were randomized to receive BMCs from KO or WT mice via the tail vein (WT → WT, KO → WT, WT → KO, KO → KO). Four weeks later, all mice were fed a HFD for 12 weeks to induce NAFLD model (*n* = 8 mice each group).

Fourth, to further confirm the effect of bone marrow-derived MYDGF on NAFLD mice. WT and KO mice aged 4–6 weeks received four injections of AAV-MYDGF or AAV-GFP at a dose of 1 × 10^12^ VP through the bone marrow cavity, including the following five groups: Control, WT-GFP, WT-MYDGF, KO-GFP, and KO-MYDGF. The control group mice were fed a NCD for 12 weeks, while the mice of the other groups were fed a HFD for 12 weeks. To assess MYDGF injection efficiency, we measured the bone marrow protein and mRNA levels of MYDGF.

Fifth, WT and KO mice aged 4–6 weeks received a single injection of AAV-siIKKβ or AAV-siCon at a dose of 1 × 10^12^ VP in 0.1 mL of phosphate buffer saline (PBS) through the tail vein. To assess IKKβ silencing efficiency, we measured the liver protein and mRNA levels of IKKβ. All mice were fed a HFD for 12 weeks to induce NAFLD (*n* = 8 mice per group).

Sixth, WT and KO mice aged 4–6 weeks received a single injection of AAV-sip65 or AAV-siCon at a dose of 1 × 10^12^ VP in 0.1 mL of PBS through the tail vein. All mice were fed a HFD for 12 weeks to induce NAFLD (*n* = 8 mice per group).

At the end of the experiments, the mice were fasted overnight and then anesthetized by intraperitoneal injection of pentobarbital sodium (60 mg/kg) and euthanized for blood and tissues samples.

#### Construction of AAV vectors for mice

The AAV-MYDGF vector was synthesized by Hanbio (Shanghai, China). The MYDGF (GenBank accession number NM_080837.2) gene sequence was directly synthesized in the pHBAAV-CMV-MCS-3flag-T2A-ZsGreen vector and then co-transfected into AAV9–293 cells with pAAV-RC and pHelper plasmids [42]. The AAV encoding liver IKKβ-specific siRNA (IKKβ: GenBank accession number NM_080012.4), p65-specific siRNA (p65: GenBank accession number L77155.1), and scrambled control siRNA (siCon) oligonucleotides were synthesized by Hanbio (Shanghai, China). The AAV9 vector system was used to achieve IKKβ and p65 specific silencing in the liver [43]. siIKKβ, sip65, and siCon were inserted into the AAV9 vector plasmid pSNAV1 under the control of the constitutive cytomegalovirus promoter to construct pSNAV1/siRNA. Large-scale recombinant AAV production, purification, and preparation were described previously [44].

#### Glucose and insulin tolerance tests

For the intraperitoneal (i.p.) glucose tolerance test (GTT), mice were fasted for 12 h, before receiving an i.p. injection of 2 g/kg glucose. For the insulin tolerance test (ITT), mice were fasted for 6 h, before receiving an i.p. injection of 0.75 U/kg insulin. At baseline and 30, 60, 90, and 120 min after glucose or insulin administration, blood samples were obtained by tail-bleeding and the glucose level was checked by a portable glucose meter (Bayer company, Germany). The incremental area under the curve (AUC) was calculated using the trapezoidal rule.

#### Mice biochemical measurements

BG was measured using a BG meter (Bayer company, Germany). Serum MYDGF was measured enzymatically using a commercially available kit (Sino Best Biological, Shanghai, China). The serum levels of IL-6, IL-1β, and TNF-α were measured using ELISA kits (R&D Systems, Inc., Minneapolis, USA). The serum fasting insulin concentration was measured by electrochemiluminescence immunoassay. The serum FFA level was determined using an enzymatic colorimetric assay kit from Roche Diagnostics (Mannheim, Germany) according to the manufacturer’s instructions. The serum levels of HDL-C, LDL-C, TG, TC, HbA1c, AST, ALT, and creatinine were determined by colorimetric assays using commercially available kit (Jiancheng Bioengineering Institute, Nanjing, China).

#### IHC, IF, and toluidine blue staining assays

For IHC analysis, mouse liver sections were deparaffinized, rehydrated, and incubated with F4/80 antibody (1:100; CST, #70076) overnight at 4 °C. After incubation with primary antibodies, the sections were incubated with horseradish peroxidase-conjugated anti-immunoglobulin secondary antibodies (Jackson Immuno Research, West Grove, PA, USA) and visualized with 3, 3′-diaminobenzidine. IF staining was performed following standard procedures. In brief, bone marrow sections were incubated with MYDGF antibody (1:200; Proteintech, #11353-1-AP). After incubation with primary antibodies, the sections were washed with PBS and incubated with the appropriate fluorescent secondary antibodies. Sections were mounted using 4′,6-diamidino-2-phenylindole (DAPI) (Molecular Probes) and imaged using a FluoView FV1000 confocal microscope (Olympus, Shinjuko, Japan) [45]. For toluidine blue staining, the femora were harvested from mice after euthanasia, and 4-μm-thick longitudinally oriented bone sections were stained with toluidine blue for histological analysis.

#### Cell culture and treatments

PMHs were isolated from 8-week-old WT mice and incubated in Dulbecco’s Modified Eagle’s Medium (DMEM) supplemented with 10% fetal bovine serum and 1% antibiotics as previously described [41]. For BMC-PMH co-culture experiments, BMCs and PMHs were non-contact co-cultured in DMEM using a Transwell. BMCs were cultured on the floor of the culture plate (lower well) and PMHs were seeded on the Transwell insert (upper well) [46, 47]. After incubation for 24 h, co-culture BMCs-PMHs were treated with 0.4 mM PA for 24 h. Oil red O staining was performed to assess fat deposition. Inflammatory responses and fat metabolism were detected by RT-PCR and WB. Regarding MYDGF intervention experiments, PMHs were isolated from 8-week-old WT mice. PMHs were incubated in DMEM containing various concentrations of rMYDGF (#10232-MY-050, R&D Systems, USA) under PA-stimulated conditions. To explore the underlying anti-steatosis and anti-inflammatory mechanisms of MYDGF in vitro, PMHs were pretreated with the siRNA oligonucleotides against IKKβ (IKKβ-siRNA) for 48 h and an inhibitor against PKCθ (PKC-theta inhibitor, 0.2 μM) for 1 h before the addition of rMYDGF (50 ng/mL). After incubation for 24 h, to mimic the in vivo hepatic steatosis, PMHs were treated with 0.4 mM PA, and the fat deposition and inflammatory profiles were detected as outlined above. To analyze hepatocyte insulin signaling, PMHs were pretreated with IKKβ siRNA for 48 h before the addition of rMYDGF (50 ng/mL). Prior to harvest, PMHs were treated with 0.4 mM PA for 24 h, insulin (100 nM) for 30 min, and subjected to the assays to examine insulin signaling. To analyze SREBP1c expression, PMHs were cultured under conditions of nutrient deprivation or sufficiency.

#### Analysis of macrophage polarization

Primary KCs were isolated from the livers of different groups of mice by perfusion using collagenase type IV (Sigma-Aldrich), as described previously [48]. KCs were assayed for macrophage subsets using a BD Accuri C6 Plus flow cytometer (BD Biosciences, San Jose, CA). Among KCs, mature macrophages (F4/80^+^) were gated for CD80 and CD206 expression (macrophage polarization) under PA stimulation. F4/80^+^CD80^+^ cells were considered pro-inflammatory (M1) macrophages, whereas F4/80^+^CD206^+^ cells were considered alternatively activated (M2) macrophages [49].

#### SiRNA silencing assay in vitro

siRNAs were synthesized by Hanbio (Shanghai, China), siIKKβ: sense sequence: 5′-CAGCUUUAAGUUUAAGAUGUCAUCCAG-3′; antisense sequence: 5′-GGAUGACAUCUUAAACUUAAAGCTG-3′. siRNA was transfected to silence IKKβ in PMHs using Lipofectamine RNAi MAX (Invitrogen, Carlsbad, CA, USA) in Opti-MEM Medium (Invitrogen, Carlsbad, CA, USA), according to the manufacturer’s instructions. Non-specific siRNA was used as a control: 5′-UUCUCCGAACGUGUCACGUTT-3′ [50].

### RT-PCR assay

RT-PCR was performed on the Applied Biosystems Prism 7000 sequence detection system, with specific primers described in Table S7. Relative changes in mRNA levels among groups were determined with the 2ΔΔCt method. Glyceraldehyde 3-phosphate dehydrogenase (GAPDH) was used as the housekeeping gene to normalize the expressions of the target genes. The thermal cycling conditions for RT-PCR were as follows: 95 °C for 30 s, followed by 40 cycles of 95 °C for 5 s, 60 °C for 30 s, and 70 °C for 30 s.

#### WB assay

The following primary antibodies were used for WB: MYDGF (1:1000, Proteintech, #11353-1-AP), P-JNK (Thr183/Tyr185) (P-JNK,1:2000, CST, #9255), JNK (1:1000, CST, #9252), P-IKKβ (Ser176/180) (P-IKKβ, 1:1000, CST, #2697), IKKβ (1:1000, CST, #2684), P-p65 (Ser468) (1:1000, CST, #3039), p65 (1:1000, CST, #8242), P-IkBα (Ser32) (1:1000, CST, #2859), IkBα (1:1000, CST, #9242), P-p38MAPK (Thr180/Tyr182) (1:2000, CST, #9216), p38MAPK (1:1000, CST, #8690), P-ERK (Thr202/Tyr204) (1:1000, CST, #4376), ERK (1:1000, CST, #4695), FAS (1:1000, CST, #3189), ACC1 (1:1000, CST, #4190), SCD1 (1:1000, CST, #2794), pSREBP1c (1:1000, Santa Cruz Biotechnology, sc-13551), nSREBP1c (1:1000, Novus Biologicals, NB100–60545), Lamin A/C (1:3000, CST, #4777), P-PKCθ (Ser643/676) (1:1000, CST, #4376), P-PKCα (Thr638) (1:1000, CST, #9375), P-PKCβ (Ser660) (1:1000, CST, #9371), P-PKCλ (Thr403) (1:1000, CST, #9378), P-IR (Tyr1316) (1:1000, Affinity Biosciences, #AF3099), IR (1:1000, Affinity Biosciences, #AF6099), P-Akt (Ser473) (1:1000, CST, #12694), Akt (1:1000, CST, #9272), and GAPDH (1:3000, CST, #5174). The secondary antibodies were peroxidase affinipure goat anti-rabbit-IgG (1:3000, Jackson ImmunoResearch Laboratories, USA, #111-035-003) and goat anti-mouse-IgG (1:3000, Jackson ImmunoResearch Laboratories, USA, #115-035-146). The WB bands were visualized using an enhanced chemiluminescence kit (PerkinElmer, Inc., Waltham, MA, USA).

#### BMT assay

According to our previous report [51], we transplanted BMCs via the tail vein into lethally irradiated recipient mice (1 × 10^6^ cells per mouse).

#### Injection of AAV into the bone marrow cavity

Mice received 5 μL of either of AAV-MYDGF or AAV-GFP (1 × 10^12^ VP/mL) via periosteal injection into the medullary cavity of the femur every 3 weeks for 12 weeks [52].

#### Measurement of hepatocyte fat deposition

PMHs were treated with or without PA for 24 h. At 1 h prior to harvest, the cells were stained with oil red O to quantify the fat content as previously described [41].

#### Measurement of hepatocyte p65 nuclear translocation

IF staining was conducted according to standard procedures. Using p65 antibody (1:200; Cell Signaling Technology, #8242) incubated sections. After incubating with primary antibodies, the sections were washed with PBS and incubated with fluorescent secondary antibodies. IF images were obtained with a FluoView FV1000 confocal microscope (Olympus, Shinjuko, Japan).

#### Measurements of cytokine levels in supernatants

The levels of TNF-α, IL-1β, and IL-6 in the supernatants of PMHs were measured using ELISA following the protocols provided by the manufacturer (R&D Systems, Inc., Minneapolis, USA).

#### Measurements of liver TG levels

TG levels were measured in frozen livers using an assay kit from Wako (Richmond, VA), and expressed as mg TG per gram liver tissue (wet) [53].

#### Molecular assays

To determine inflammatory signaling and lipogenesis, lysates of frozen livers or cultured PMHs were subjected to WB analysis, as previously described [54, 55]. In addition, cytosolic and nuclear fractions of livers or PMHs were analyzed for SREBP1c abundance using WB analysis. The maximum intensity of each band was quantified using ImageJ software. Ratios of P-PKCθ/PKCθ, P-PKCα/PKCα, P-PKCβ/PKCβ, P-PKCλ/PKCλ, P-IKKβ/IKKβ, P-p65/p65, P-IkBα/IkBα, P-JNK/JNK, P-p38MAPK/p38MAPK, P-ERK/ERK, FAS, ACC1, and SCD1 were normalized to GAPDH, while pSREBP1c and nSREBP1c were normalized to LaminA/C, and adjusted relative to the average of HFD-fed WT + GFP mice or KO + GFP mice, or NCD-fed control, or PBS- or siCon-treated control, which were set as 1 AU. To examine gene expression, the total RNA was isolated from frozen liver tissues and cultured/isolated cells, and subjected to reverse transcription and RT-PCR analysis. The results were normalized to 18 s ribosomal RNA and plotted as relative expression to the average of the NCD-fed control, HFD-fed WT + GFP mice, KO + GFP mice, or control cells with or without PA treatment, which were set as 1.

#### Blood pressures and other parameters

Blood pressures, including SBP and diastolic blood pressure (DBP) were noninvasively measured by the tail-cuff method (Softron BP-98A, Tokyo, Japan). Blood pressure values were averaged from three consecutive measurements under steady-state conditions. Food intake, fecal output, and lipid content in feces were measured as previously described [41].

#### Luciferase assay

The NF-κB luciferase reporter plasmid was constructed by Hanbio (Shanghai, China). Luciferase reporter plasmids or control plasmids were transfected into PMHs with the JET-PEI transfection system (Polyplus). After 24 h, cells were treated with MYDGF 50 ng/mL for 24 h, before adding PA 0.4 mmol/L for an additional 24 h before the end. Luciferase and Renilla were determined by the dual-luciferase reporter assay system (Promega) according to the manufacturer’s instructions.

#### ChIP assay

PMHs were transfected with siIKKβ or siCon. After 12 h, cells were treated with MYDGF 50 ng/mL for 24 h, before adding PA 0.4 mmol/L for an additional 24 h before the end. ChIP was performed using the Simplechip enzymatic chromatin IP kit (Cell Signaling Technology) according to the manufacturer’s instructions. The p65 antibody (ab19870) or IgG (ab2410) were used for p65 immunoprecipitation. The primers for SREBP1c and IκBα are listed in Supplementary Table 7. RT-PCR was used to amplify the target genes.

#### Biosynthesis of fatty acids

PMHs were treated with PA as described previously. The media was removed from the plates, before adding fresh medium with sodium [14 C] acetate for 6 h. After washing the labeled cells, the cells were dissolved by 0.1 N NaOH. Prior to saponification, 500 μL of 75% KOH, 10 μL of [3H] cholesterol nonsaponified TLC recovery carrier solution, and 10 μL [3H] of oleic acid carrier solution were added to each sample. After extraction with petroleumether, the fatty acid fraction was dried under nitrogen gas and dissolved in 30 μL of chloroform for fatty acid. The extract samples were spotted onto plastic-backed silica gel TLC plates before staining with iodine vapor. Excise spots were excised and placed in scintillation vials including 10 mL scintillation solution to count [14 C] and [3H].

#### Analysis of peripheral blood cells

Ten-week-old WT and KO mice were weighed and anesthetized by intraperitoneal injection of pentobarbital sodium. Blood was collected from the internal canthus vein, EDTA anticoagulation. The blood count was used to determine the peripheral blood count and perform classification. Immune cells from peripheral blood were incubated with labeled antibodies. The NK1.1-FITC fluorescent-labeled antibody was purchased from Biolegend; CD11b-APC was purchased from Invitrogen; B220–PerCP, F4/80–PE, CD4-PE-Cy, and CD8a-APC-Cy7 were purchased from BD; B220–PerCP-Cy5.5 and CD3-APC were purchased from Biolegend Division; EDTA was purchased from Sigma; and Erythrocyte lysate was purchased from Invitrogen. We then added the cells to TruCOUNT tubes (BD Biosciences) and counted them on an LSR II flow cytometer (Becton Dickinson), before analyzing with FlowJo software (version 10).

#### Statistical analysis

Data are expressed as the mean ± standard error of mean (SEM). Pearson correlation analysis was used to identify correlations between variables. Data between groups were compared by the unpaired Student’s t-test or one-way ANOVA followed by Tukey’s post-test for multiple comparisons. *P* < 0.05 was considered significant.

## Supplementary information

supplementary material

Original Data File

Figure S14B

## Data Availability

The datasets generated and/or analyzed during the current study are available from the corresponding author on reasonable request.
