# Correction: Myeloid-derived growth factor alleviates non-alcoholic fatty liver disease alleviates in a manner involving IKKβ/NF-κB signaling

**DOI:** 10.1038/s41419-025-07486-3

**Published:** 2025-06-02

**Authors:** Yan Ding, Xiaoli Xu, Biying Meng, Li Wang, Biao Zhu, Bei Guo, Jiajia Zhang, Lin Xiang, Jing Dong, Min Liu, Guangda Xiang

**Affiliations:** 1 Department of Endocrinology, General Hospital of Central Theater Command, Wuluo Road 627, Wuhan, 430070 Hubei Province China; 2 Department of Diagnostics, School of Medicine, Hunan University of Medicine, Huaihua, 418000 Hunan Province China; 3 The First School of Clinical Medicine, Southern Medical University, NO.1023, South Shatai Road, Guangzhou, 510515 Guangdong Province China

**Keywords:** Non-alcoholic fatty liver disease, Mechanisms of disease, Obesity

Correction to: *Cell Death and Disease* 10.1038/s41419-023-05904-y, published online 26 June 2023

After repeatedly checking the original data and images in the article, I found that two images were misplaced. The first one is the oil red O staining image of the sip65-transfected KO (KO+sip65) mice group in Fig. 8B, and the other image is the P-IR protein band from the Full unedited blot for FigureS 14B in the Original Data File. The text corresponding to Fig. 8B and FigureS 14B in the article does not need to be corrected. These corrections do not impact the overall findings and conclusions of the paper.

Original Figure 8
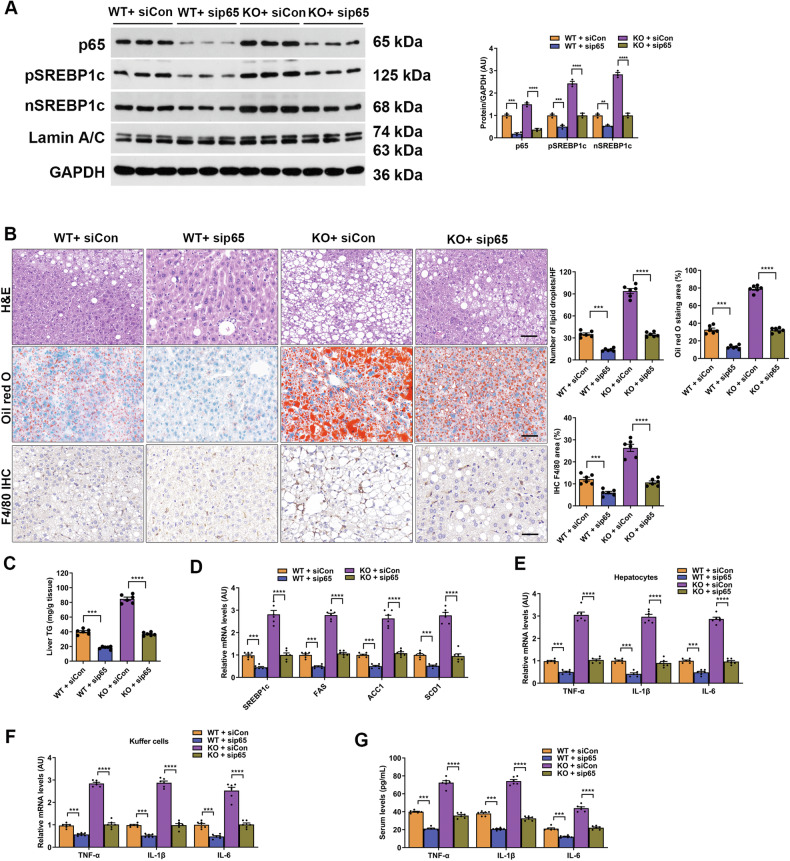


Corrected Figure 8
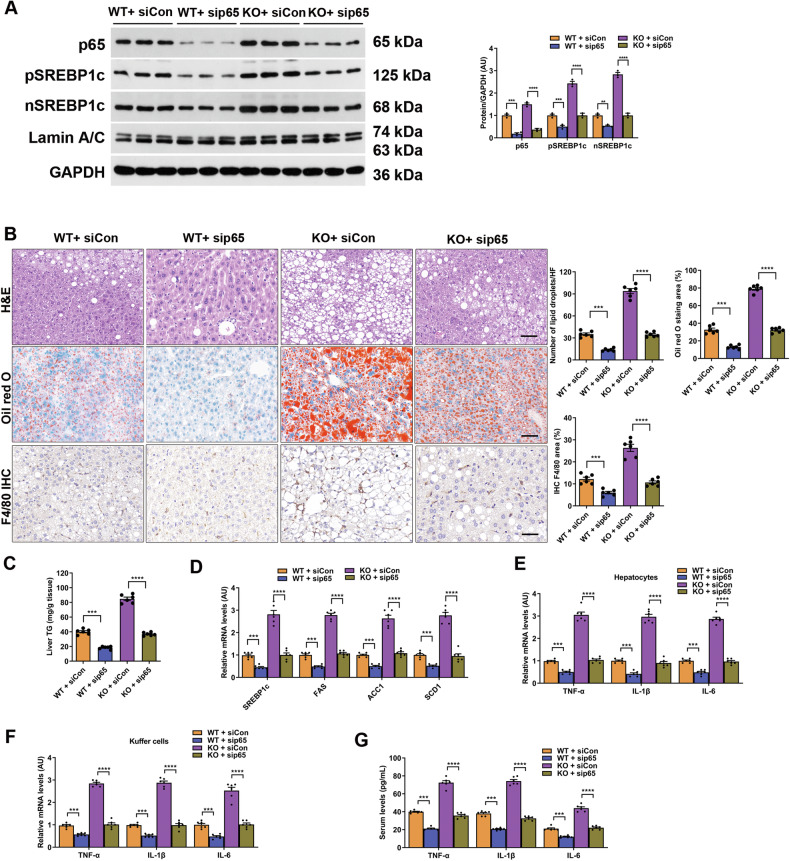


Original FigureS 14B
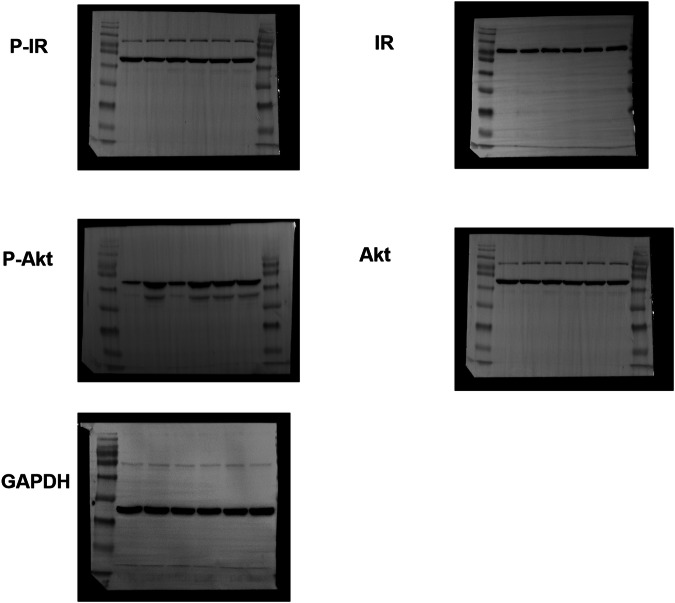


Corrected FigureS 14B
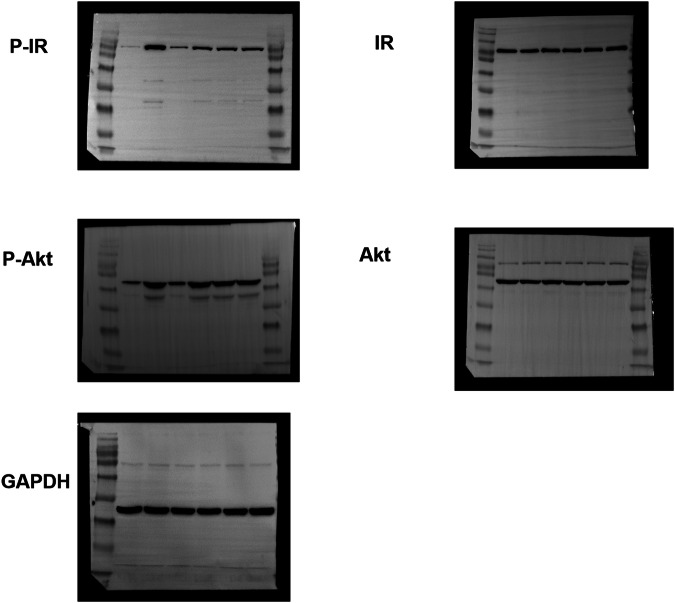


The original article has been corrected.

## Supplementary information

supplementary material

Original Data File

Reproducibility checklist

