# Supplementary material for: Myeloid-derived growth factor alleviates non-alcoholic fatty liver disease alleviates in a manner involving IKKβ/NF-κB signaling
Source: Cell Death Dis. 2023 Jun 26;14(6):376. doi: 10.1038/s41419-023-05904-y (PMC10293205; doi:10.1038/s41419-023-05904-y)
Supplement: Supplementary file 1 — supplementary material [file 41419_2023_5904_MOESM1_ESM.pdf]

**Supplementary Table 1. Clinical and biochemical characteristics in control and NAFLD subjects.**

| Variables | Control | NAFLD | *P* values |
|---|---|---|---|
| N | 60 | 60 | - |
| Age (year) | 47.33 ± 0.83 | 47.18 ± 0.84 | 0.899 |
| BMI (kg/m$^2$) | 21.86 ± 0.21 | 27.93 ± 0.19 | < 0.001 |
| SBP (mmHg) | 113.25 ± 1.17 | 121.43 ± 1.14 | < 0.001 |
| DBP (mmHg) | 78.20 ± 0.66 | 79.68 ± 0.71 | 0.131 |
| FBG (mmol/L) | 5.09 ± 0.05 | 5.23 ± 0.06 | 0.098 |
| 2h BG (mmol/L) | 6.89 ± 0.06 | 7.01 ± 0.05 | 0.111 |
| HbA1c (%) | 5.24 ± 0.06 | 5.41 ± 0.09 | 0.145 |
| Insulin (mU/L) | 9.91 ± 0.19 | 20.67 ± 0.43 | < 0.001 |
| HOMA-IR | 2.26 ± 0.05 | 4.96 ± 0.11 | < 0.001 |
| TC (mmol/L) | 4.05 ± 0.55 | 5.30 ± 0.06 | < 0.001 |
| TG (mmol/L) | 1.11 ± 0.04 | 2.47 ± 0.06 | < 0.001 |
| LDL-C (mmol/L) | 1.98 ± 0.03 | 3.14 ± 0.07 | < 0.001 |
| HDL-C (mmol/L) | 1.17 ± 0.03 | 1.00 ± 0.02 | < 0.001 |
| FFA (mmol/L) | 0.42 ± 0.01 | 0.93 ± 0.02 | < 0.001 |
| ALT (U/L) | 24.77 ± 0.84 | 58.20 ± 2.15 | < 0.001 |
| AST (U/L) | 20.36 ± 0.83 | 42.40 ± 1.58 | < 0.001 |
| TNF-α (pg/mL) | 23.32 ± 0.40 | 32.24 ± 0.37 | < 0.001 |
| IL-1β (pg/mL) | 28.14 ± 0.60 | 41.75 ± 0.64 | < 0.001 |
| IL-6 (pg/mL) | 10.95 ± 0.25 | 17.30 ± 0.20 | < 0.001 |
| Creatinine (mg/dL) | 0.83 ± 0.03 | 0.79 ± 0.03 | 0.315 |
| MYDGF (ng/mL) | 4.90 ± 0.12 | 3.58 ± 0.09 | < 0.001 |

Data are expressed as mean ± SEM. Significant differences were determined by Student's *t*-test.

Abbreviations: BMI, body mass index. SBP, systolic blood pressure. DBP, diastolic blood pressure. FBG, fasting blood glucose. 2h BG, 2 hour blood glucose. HbA1C, glycosylated hemoglobin. HOMA-IR, homeostasis model assessment of insulin resistance. TC, cholesterol. TG, triglycerides. LDL-C, low-density lipoprotein-cholesterol. HDL-C, high-density lipoprotein-cholesterol. FFA, free fatty acid. ALT, alanine aminotransferase. AST, aspartate aminotransferase. TNF-α, tumor necrosis factor-α. IL-1β, interleukin-1β. IL-6, interleukin-6. MYDGF, myeloid-derived growth factor.

**Supplementary Table 2. Biochemical characteristics in control and NAFLD mice at the end of the experiments.**

| Variables | Controls | NAFLD | *P* values |
|---|---|---|---|
| N | 10 | 10 | - |
| Body weight G | 26.42 ± 0.49 | 30.80 ± 0.81 | < 0.001 |
| SBP (mmHg) | 106.00 ± 1.40 | 108.20 ± 1.59 | 0.313 |
| DBP (mmHg) | 74.10 ± 0.91 | 76.30 ± 1.17 | 0.154 |
| FBG (mmol/L) | 5.16 ± 0.11 | 5.29 ± 0.12 | 0.441 |
| HbA1c (%) | 5.23 ± 0.21 | 5.30 ± 0.15 | 0.790 |
| Insulin (mU/L) | 9.40 ± 0.33 | 13.65 ± 0.32 | < 0.001 |
| TC (mmol/L) | 3.11 ± 0.08 | 4.79 ± 0.09 | < 0.001 |
| TG (mmol/L) | 1.04 ± 0.05 | 2.41 ± 0.07 | < 0.001 |
| LDL-C (mmol/L) | 0.95 ± 0.05 | 1.95 ± 0.04 | < 0.001 |
| HDL-C (mmol/L) | 1.52 ± 0.02 | 1.31 ± 0.03 | < 0.001 |
| FFA (mmol/L) | 0.51 ± 0.02 | 0.81 ± 0.02 | < 0.001 |
| ALT (U/L) | 44.70 ± 2.72 | 60.40 ± 2.36 | < 0.001 |
| AST (U/L) | 41.95 ± 1.53 | 56.29 ± 2.24 | < 0.001 |
| TNF-α (pg/mL) | 20.80 ± 1.04 | 35.30 ± 1.08 | < 0.001 |
| IL-1β (pg/mL) | 27.70 ± 1.18 | 32.50 ± 0.86 | < 0.001 |
| IL-6 (pg/mL) | 13.10 ± 1.37 | 21.90 ± 3.87 | < 0.001 |
| Creatinine (mg/dL) | 0.42 ± 0.01 | 0.44 ± 0.02 | 0.707 |
| MYDGF (pg/mL) | 188.20 ± 3.74 | 122.20 ± 6.46 | < 0.001 |

Data are expressed as mean ± SEM. Significant differences were determined by Student's *t*-test.

Abbreviations: SBP, systolic blood pressure. DBP, diastolic blood pressure. FBG, fasting blood glucose. HbA1C, glycosylated hemoglobin. TC, cholesterol. TG, triglycerides. LDL-C, low-density lipoprotein-cholesterol. HDL-C, high-density lipoprotein-cholesterol. FFA, free fatty acid. ALT, alanine aminotransferase. AST, aspartate aminotransferase. TNF-α, tumor necrosis factor-α. IL-1β, interleukin-1β. IL-6, interleukin-6. MYDGF, myeloid-derived growth factor.

**Supplementary Table 3. Cell characteristics of peripheral blood in WT and KO mice.**

| Variables | WT | KO | *P* values |
|---|---|---|---|
| N | 10 | 10 | - |
| WBC ($10^9$/L) | 4.04 ± 0.25 | 4.17 ± 0.29 | 0.738 |
| RBC ($10^{12}$/L) | 7.65 ± 0.28 | 7.80 ± 0.29 | 0.715 |
| PLT ($10^9$/L) | 749.50 ± 24.10 | 774.60 ± 26.43 | 0.492 |
| Lymph ($10^9$/L) | 3.34 ± 0.20 | 3.54 ± 0.27 | 0.561 |
| Gran ($10^9$/L) | 0.48 ± 0.03 | 0.54 ± 0.04 | 0.318 |
| Mon ($10^9$/L) | 0.14 ± 0.02 | 0.17 ± 0.01 | 0.140 |
| Lymph (%) | 65.30 ± 1.64 | 67.32 ± 1.91 | 0.433 |
| Gran (%) | 17.68 ± 1.21 | 19.55 ± 0.62 | 0.185 |
| Mon (%) | 3.64 ± 0.19 | 4.17 ± 0.25 | 0.114 |
| CD4 (%) | 12.22 ± 0.39 | 12.25 ± 0.31 | 0.921 |
| CD8a (%) | 10.99 ± 0.44 | 10.85 ± 0.40 | 0.817 |
| B220 (%) | 61.85 ± 0.47 | 61.52 ± 0.56 | 0.663 |
| NK1.1 (%) | 8.43 ± 0.25 | 8.37 ± 0.20 | 0.853 |
| CD4/CD8 | 1.13 ± 0.05 | 1.15 ± 0.06 | 0.799 |

Data are expressed as mean ± SEM. Significant differences were determined by Student's *t*-test.

Abbreviations: WBC, white blood cell. RBC, red blood cell. PLT, platelets. Lymph, lymphocyte. Gran, granulocyte. Mon, monocyte.

**Supplementary Table 4. Effects of bone marrow specific MYDGF deficiency on biochemical characteristics in WT and KO mice at the end of the study.**

| Variables | WT-NCD | KO-NCD | WT-HFD | KO-HFD |
|---|---|---|---|---|
| N | 6 | 6 | 6 | 6 |
| Body weight G | 27.00 ± 0.25 | 31.57 ± 0.54$^{\#}$ | 31.65 ± 0.42 | 36.96 ± 0.30$^{*}$ |
| Food intake (g/week) | 19.83 ± 0.87 | 20.80 ± 0.61 | 20.68 ± 0.48 | 21.00 ± 0.86 |
| Fecal output (g/week) | 2.22 ± 0.11 | 2.40 ± 0.10 | 2.30 ± 0.09 | 2.35 ± 0.07 |
| Lipid content in fecal (mg/g) | 21.67 ± 0.99 | 39.93 ± 0.75$^{\#}$ | 41.33 ± 1.15 | 43.67 ± 1.52 |
| SBP (mmHg) | 105.83 ± 4.07 | 105.00 ± 2.40 | 106.00 ± 3.90 | 107.01 ± 1.70 |
| DBP (mmHg) | 74.00 ± 1.46 | 71.83 ± 1.94 | 74.67 ± 1.60 | 75.50 ± 1.31 |
| FBG (mmol/L) | 5.12 ± 0.09 | 5.01 ± 0.11 | 5.13 ± 0.13 | 5.25 ± 0.12 |
| HbA1c (%) | 5.25 ± 0.11 | 5.22 ± 0.10 | 5.38 ± 0.15 | 5.50 ± 0.14 |
| Insulin (mU/L) | 9.37 ± 0.47 | 14.20 ± 0.63$^{\#}$ | 13.68 ± 0.60 | 19.78 ± 0.49$^{*}$ |
| TC (mmol/L) | 3.08 ± 0.09 | 4.27 ± 0.16$^{\#}$ | 4.66 ± 0.16 | 6.35 ± 0.17$^{*}$ |
| TG (mmol/L) | 1.01 ± 0.06 | 2.22 ± 0.0.8$^{\#}$ | 2.63 ± 0.07 | 3.68 ± 0.07$^{*}$ |
| FFA (mmol/L) | 0.42 ± 0.03 | 0.58 ± 0.03$^{\#}$ | 0.75 ± 0.04 | 1.47 ± 0.05$^{*}$ |
| LDL-C (mmol/L) | 1.02 ± 0.07 | 1.57 ± 0.03$^{\#}$ | 1.85 ± 0.03 | 2.02 ± 0.06$^{*}$ |
| HDL-C (mmol/L) | 1.43 ± 0.03 | 1.30 ± 0.03$^{\#}$ | 1.33 ± 0.02 | 1.17 ± 0.03$^{*}$ |
| Creatinine (mg/dL) | 0.45 ± 0.03 | 0.44 ± 0.03 | 0.43 ± 0.02 | 0.47 ± 0.03 |
| MYDGF (pg/mL) | 181.00 ± 4.34 | 7.70 ± 0.55$^{\#}$ | 110.10 ± 5.61 | 6.70 ± 0.45$^{*}$ |

Data are expressed as mean ± SEM. All mice were fed a NCD or HFD for 12 weeks. $^{\#}P < 0.01$ vs. WT NCD. $^{*}P < 0.01$ vs. WT HFD. One-way ANOVA followed by Tukey's post-test for multiple comparisons was used for groups of three or more. Abbreviations: SBP, systolic blood pressure. DBP, diastolic blood pressure. FBG, fasting blood glucose. HbA1C, glycosylated hemoglobin. TC, cholesterol. TG, triglycerides. FFA, free fatty acid. LDL-C, low-density lipoprotein-cholesterol. HDL-C, high-density lipoprotein-cholesterol. MYDGF, myeloid-derived growth factor.

**Supplementary Table 5. Effects of MYDGF restoration by BMT on biochemical characteristics in WT and KO mice at the end of the study.**

| Variables | Control | WT→WT | KO→WT | WT→KO | KO→KO |
|---|---|---|---|---|---|
| N | 6 | 6 | 6 | 6 | 6 |
| Body weight G | 27.60 ± 0.22 | 31.32 ± 0.51 | 36.70 ± 0.20$^{\#}$ | 31.98 ± 0.31 | 36.62 ± 0.32$^{*}$ |
| Food intake (g/week) | 19.50 ± 1.13 | 21.81 ± 1.05 | 21.12 ± 0.63 | 21.05 ± 0.87 | 21.67 ± 0.94 |
| Fecal output (g/week) | 2.16 ± 0.08 | 2.27 ± 0.09 | 2.17 ± 0.08 | 2.25 ± 0.10 | 2.20 ± 0.08 |
| Lipid content in fecal (mg/g) | 23.83 ± 0.70 | 43.83 ± 1.54 | 44.67 ± 1.69 | 44.33 ± 1.67 | 45.67 ± 1.17 |
| SBP (mmHg) | 105.17 ± 1.83 | 106.17 ± 1.35 | 105.33 ± 1.28 | 107.17 ± 1.25 | 105.50 ± 1.61 |
| DBP (mmHg) | 74.67 ± 1.93 | 76.67 ± 1.02 | 74.50 ± 1.98 | 75.50 ± 1.50 | 75.00 ± 1.37 |
| FBG (mmol/L) | 5.15 ± 0.14 | 5.22 ± 0.12 | 5.18 ± 0.08 | 5.62 ± 0.34 | 5.32 ± 0.22 |
| HbA1c (%) | 5.20 ± 0.20 | 5.35 ± 0.11 | 5.28 ± 0.16 | 5.37 ± 0.17 | 5.32 ± 0.12 |
| Insulin (mU/L) | 8.70 ± 0.21 | 13.63 ± 0.67 | 19.45 ± 0.48$^{\#}$ | 13.47 ± 0.63 | 19.63 ± 0.62$^{*}$ |
| TC (mmol/L) | 3.23 ± 0.07 | 4.68 ± 0.15 | 6.38 ± 0.16$^{\#}$ | 4.72 ± 0.11 | 6.37 ± 0.17$^{*}$ |
| TG (mmol/L) | 0.98 ± 0.05 | 2.67 ± 0.08 | 3.61 ± 0.11$^{\#}$ | 2.73 ± 0.11 | 3.67 ± 0.08$^{*}$ |
| FFA (mmol/L) | 0.39 ± 0.01 | 0.73 ± 0.03 | 1.42 ± 0.06$^{\#}$ | 0.75 ± 0.03 | 1.46 ± 0.04$^{*}$ |
| LDL-C (mmol/L) | 1.04 ± 0.05 | 1.83 ± 0.03 | 2.06 ± 0.05$^{\#}$ | 1.85 ± 0.07 | 2.05 ± 0.05$^{*}$ |
| HDL-C (mmol/L) | 1.43 ± 0.02 | 1.31 ± 0.02 | 1.12 ± 0.04$^{\#}$ | 1.28 ± 0.03 | 1.14 ± 0.03$^{*}$ |
| Creatinine (mg/dL) | 0.43 ± 0.01 | 0.42 ± 0.02 | 0.44 ± 0.02 | 0.45 ± 0.02 | 0.45 ± 0.03 |
| MYDGF (pg/mL) | 181.50± 4.78 | 115.10± 5.47 | 21.04 ± 0.85$^{\#}$ | 112.90 ± 5.63 | 6.33 ± 0.47$^{*}$ |

Data are expressed as mean ± SEM. NAFLD group was fed a HFD for 12 weeks, and control group was fed a NCD for 12 weeks. $^{\#}P < 0.01$ vs. WT→WT. $^{*}P < 0.01$ vs. WT→KO. One-way ANOVA followed by Tukey's post-test for multiple comparisons was used for groups of three or more.

Abbreviations: SBP, systolic blood pressure. DBP, diastolic blood pressure. FBG, fasting blood glucose. HbA1C, glycosylated hemoglobin. TC, cholesterol. TG, triglycerides. FFA, free fatty acid. LDL-C, low-density lipoprotein-cholesterol. HDL-C, high-density lipoprotein-cholesterol. MYDGF, myeloid-derived growth factor.

**Supplementary Table 6. Effects of MYDGF restoration by intramarrow injection on biochemical characteristics in WT and KO mice at the end of the study.**

| Variables | Control | WT + GFP | WT+ MYDGF | KO + GFP | KO + MYDGF |
|---|---|---|---|---|---|
| N | 6 | 6 | 6 | 6 | 6 |
| Body weight G | 28.13 ± 0.23 | 32.55 ± 0.58 | 30.25 ± 0.39$^{\#\#}$ | 36.37 ± 0.36 | 32.55 ± 0.43$^{**}$ |
| Food intake (g/week) | 19.82 ± 0.69 | 21.30 ± 0.49 | 21.42 ± 0.62 | 21.50 ± 0.60 | 21.08 ± 0.53 |
| Fecal output (g/week) | 2.12 ± 0.09 | 2.28 ± 0.05 | 2.17 ± 0.06 | 2.18 ± 0.08 | 2.15 ± 0.07 |
| Lipid content in fecal (mg/g) | 22.58 ± 0.84 | 43.70 ± 1.40 | 45.25 ± 1.50 | 44.70 ± 1.53 | 44.07 ± 1.30 |
| SBP (mmHg) | 105.00 ± 2.30 | 110.00 ± 2.90 | 106.00 ± 2.38 | 112.00 ± 2.82 | 109.50 ± 1.65 |
| DBP (mmHg) | 74.83 ± 2.15 | 79.17 ± 2.50 | 74.33 ± 2.58 | 80.67 ± 3.20 | 78.67 ± 1.52 |
| FBG (mmol/L) | 5.21 ± 0.27 | 5.42 ± 0.26 | 5.22 ± 0.26 | 5.62 ± 0.34 | 5.32 ± 0.22 |
| HbA1c (%) | 5.13 ± 0.26 | 5.38 ± 0.22 | 5.34 ± 0.22 | 5.78 ± 0.19 | 5.48 ± 0.13 |
| Insulin (mU/L) | 8.72 ± 0.23 | 12.45 ± 0.35 | 9.28 ± 0.23$^{\#\#}$ | 20.82 ± 0.61 | 12.58 ± 0.55$^{**}$ |
| TC (mmol/L) | 3.24 ± 0.10 | 4.70 ± 0.11 | 3.51 ± 0.08$^{\#\#}$ | 6.45 ± 0.12 | 4.69 ± 0.18$^{**}$ |
| TG (mmol/L) | 1.03 ± 0.06 | 2.63 ± 0.06 | 1.23 ± 0.05$^{\#\#}$ | 3.58 ± 0.10 | 2.68 ± 0.12$^{**}$ |
| FFA (mmol/L) | 0.46 ± 0.03 | 0.77 ± 0.03 | 0.45 ± 0.02$^{\#\#}$ | 1.31 ± 0.04 | 0.79 ± 0.02$^{**}$ |
| LDL-C (mmol/L) | 0.98 ± 0.04 | 1.90 ± 0.04 | 1.27 ± 0.05$^{\#\#}$ | 2.13 ± 0.08 | 1.39 ± 0.04$^{**}$ |
| HDL-C (mmol/L) | 1.47 ± 0.03 | 1.29 ± 0.03 | 1.40 ± 0.03$^{\#}$ | 1.19 ± 0.04 | 1.34 ± 0.02$^{*}$ |
| Creatinine (mg/dL) | 0.43 ± 0.02 | 0.42 ± 0.01 | 0.46 ± 0.03 | 0.45 ± 0.02 | 0.43 ± 0.02 |
| MYDGF (pg/mL) | 181.50± 4.29 | 115.90± 5.05 | 164.70± 4.12$^{\#\#}$ | 6.29 ± 0.33 | 152.50±3.26$^{**}$ |

Data are expressed as mean ± SEM. NAFLD group was fed a HFD for 12 weeks, and control group was fed a NCD for 12 weeks. $^{\#}P < 0.05$, $^{\#\#}P < 0.01$ vs. WT + GFP. $^{*}P < 0.05$, $^{**}P < 0.01$ vs. KO + GFP. One-way ANOVA followed by Tukey's post-test for multiple comparisons was used for groups of three or more.

Abbreviations: SBP, systolic blood pressure. DBP, diastolic blood pressure. FBG, fasting blood glucose. HbA1C, glycosylated hemoglobin. TC, cholesterol. TG, triglycerides. FFA, free fatty acid. LDL-C, low-density lipoprotein-cholesterol. HDL-C, high-density lipoprotein-cholesterol. MYDGF, myeloid-derived growth factor.

**Supplementary Table 7. A list of RT-PCR primers used in this study.**

| Primer name | Forward primer (5’ to 3’) | Reverse primer (5’ to 3’) |
|---|---|---|
| MYDGF | CTGAAGTGAGTCCGGGAGC | AGTGGGAAAGGGACGGGTG |
| SREBP1c | CTGGGGGTGAGACAGGGGAC | GATGGTGGAGGGGACAAGGG |
| FAS | CTGCGGAAACTTCAGGAAATG | GGTTCGGAATGCTATCCAGG |
| ACC1 | GGCCAGTGCTATGCTGAGAT | AGGGTCAAGTGCTGCTCCA |
| SCD1 | GAGGCCTGTACGGGATCATA | TGAGAGAAGAAGAAGCCACGG |
| CPT1a | TGGGCTACTCAGAGGATGG | AAGGTGTCAAATGGGAAGG |
| ACOX1 | GCCAAGGCGACCTGAGTGAGC | ACCGCAAGCCATCCGACATTC |
| PPARα | TCTGTGGGCTCACTGTTCT | AGGGCTCATCCTGTCTTTG |
| TNF-α | AGCCCCCAGTCTGTATCCTT | CTCCCTTTGCAGAACTCAGG |
| IL-1β | CCGTGGACCTTCCAGGATGA | GGGAACGTCACACACCAGCA |
| IL-6 | ACTCCAGAAGACCAGAGGAAAT | CCAGAGATACAAAGAAATGATGG |
| IKKβ | AGCTCTGGAACCTCCTGAAGA | AGCCTCGATCTAGGGTCGTGA |
| GAPDH | TCAACAGCAACTCCCACTCTTCCA | TTGTCATTGAGAGCAATG CCAGCC |
| SREBP1c (ChIP) | CAAACCCCTTCACCTGGGTT | CCAACTCTCCAGGAGCCAAG |
| IKBα (ChIP) | AAGCTGAGGCAGAGGTCCTA | GGGTGGTTTGAGACAGGGTT |

Abbreviations: MYDGF, myeloid-derived growth factor. SREBP1c, sterol regulatory element-binding protein 1c. FAS, fatty acid synthase. ACC1, acetyl-CoA carboxylase 1. SCD1, stearoyl-CoA desaturase 1. CPT1a, carnitine palmitoyltransferase 1a. PPARα, peroxisome proliferators-activated receptors alpha. TNF-α, tumor necrosis

factor-α. IL-1β, interleukin-1β. IL-6, interleukin-6. IKKβ, inhibitor kappa B kinase beta. GAPDH, glyceraldehyde 3-phosphate dehydrogenase. IKBα, I-kappa-B-alpha.

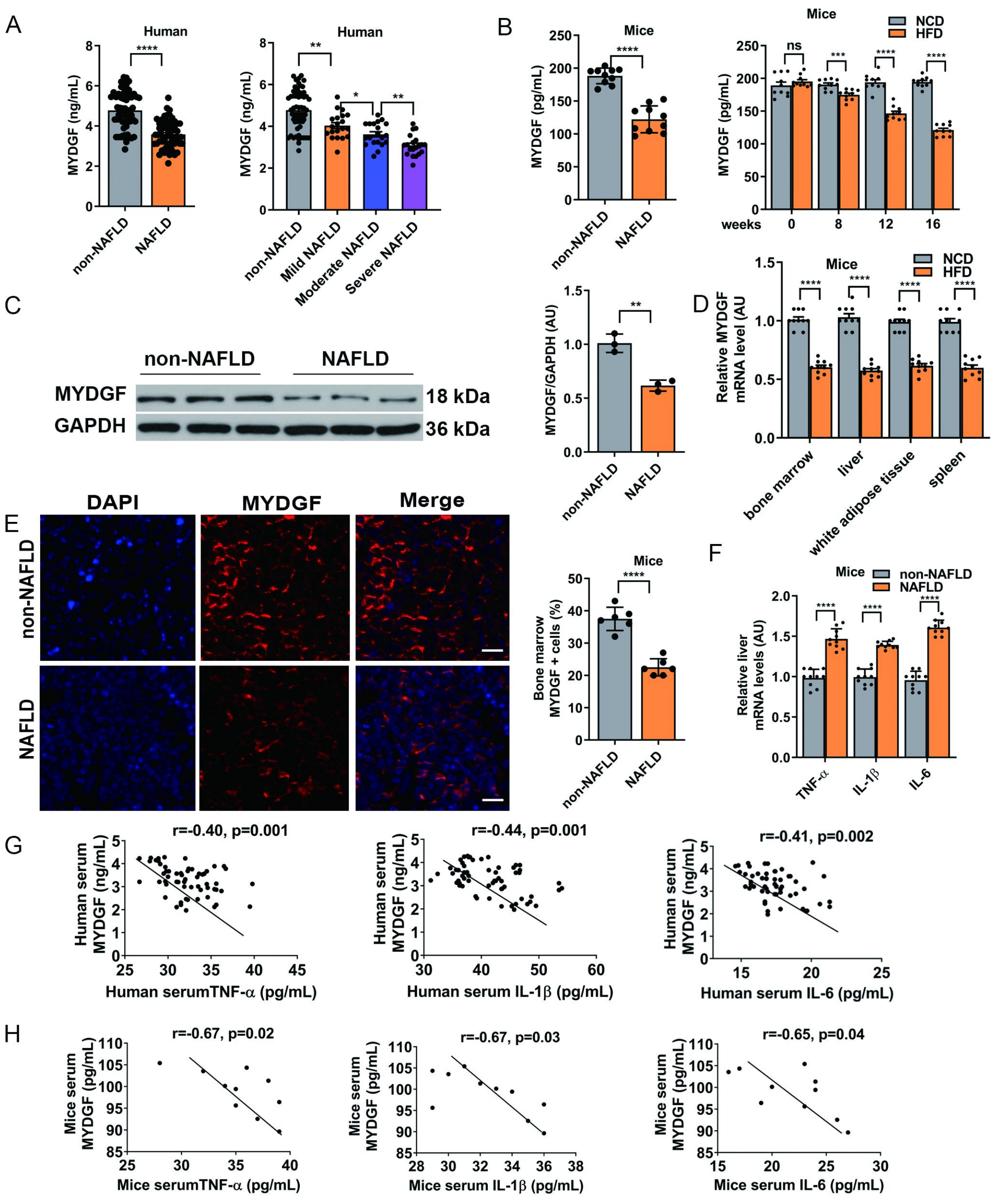


**Supplementary Fig. 1 Reduced MYDGF levels and enhanced inflammation in NAFLD patients and mice. A** The serum MYDGF levels in patients with NAFLD and healthy subjects (n = 60), and in patients with mild, moderate, severe NAFLD and healthy subjects. **B** The serum MYDGF levels in NAFLD mice and non-NAFLD mice and in different time point in NAFLD mice (n = 10). **C** Mice bone marrow MYDGF protein levels (n = 3). **D** Mice bone marrow MYDGF mRNA levels (n = 10).

**E** Expression of MYDGF (red) in mice bone marrow (n = 6). Nuclei are shown in blue. White bar = 20 μm. **F** TNF-α, IL-1β and IL-6 mRNA levels in the liver ( n= 10). **G** Correlation between serum MYDGF levels and serum TNF-α, IL-1β and IL-6 levels in NAFLD patients (n = 60). **H** Correlation between serum MYDGF levels and serum TNF-α, IL-1β and IL-6 levels in NAFLD mice (n = 10). Data are presented as the mean ± SEM except where noted. $^{*}P < 0.05$, $^{**}P < 0.01$, $^{***}P < 0.001$, $^{****}P < 0.0001$. Significant differences were determined by Student's *t*-test or One-way ANOVA followed by Tukey's post-test.

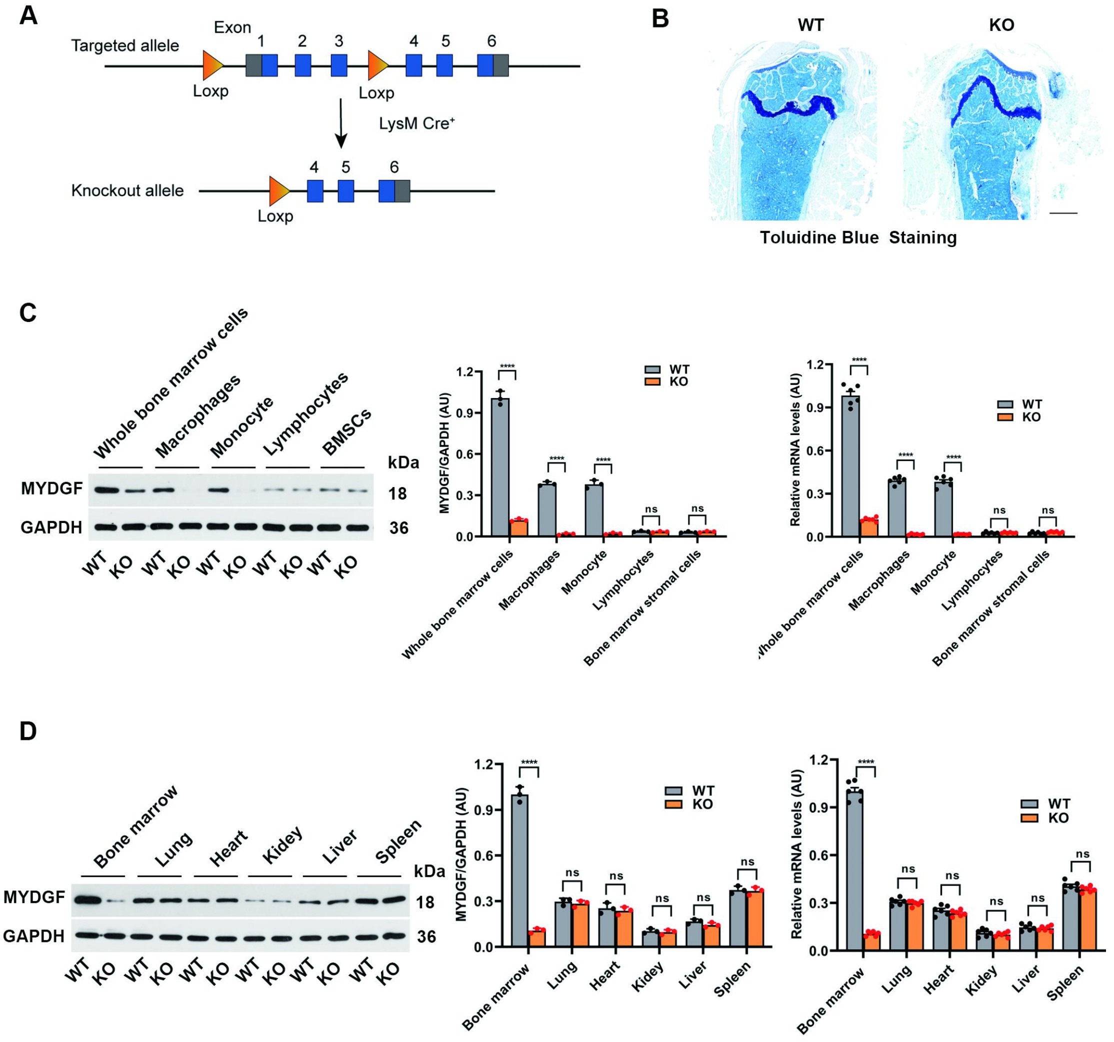


**Supplementary Fig. 2 Bone marrow integrity and the expression of MYDGF in WT and KO mice. A** The strategy of myeloid cell specific MYDGF knockout mice. **B** Representative images showing toluidine blue staining of distal femur sections in WT and KO mice aged 12 weeks. Black bar = 500 μm. **C** Mice MYDGF protein levels (n = 3) and mRNA levels (n = 6) in whole bone marrow cells, hematopoietic cell subtypes and bone marrow stromal cells of WT and KO mice. **D** Mice MYDGF protein levels (n = 3) and mRNA levels (n = 6) in other tissues of WT and KO mice. $^{*}P < 0.05$, $^{**}P < 0.01$, $^{***}P < 0.001$, $^{****}P < 0.0001$. Significant differences were determined by Student's *t*-test or One-way ANOVA followed by Tukey's post-test.

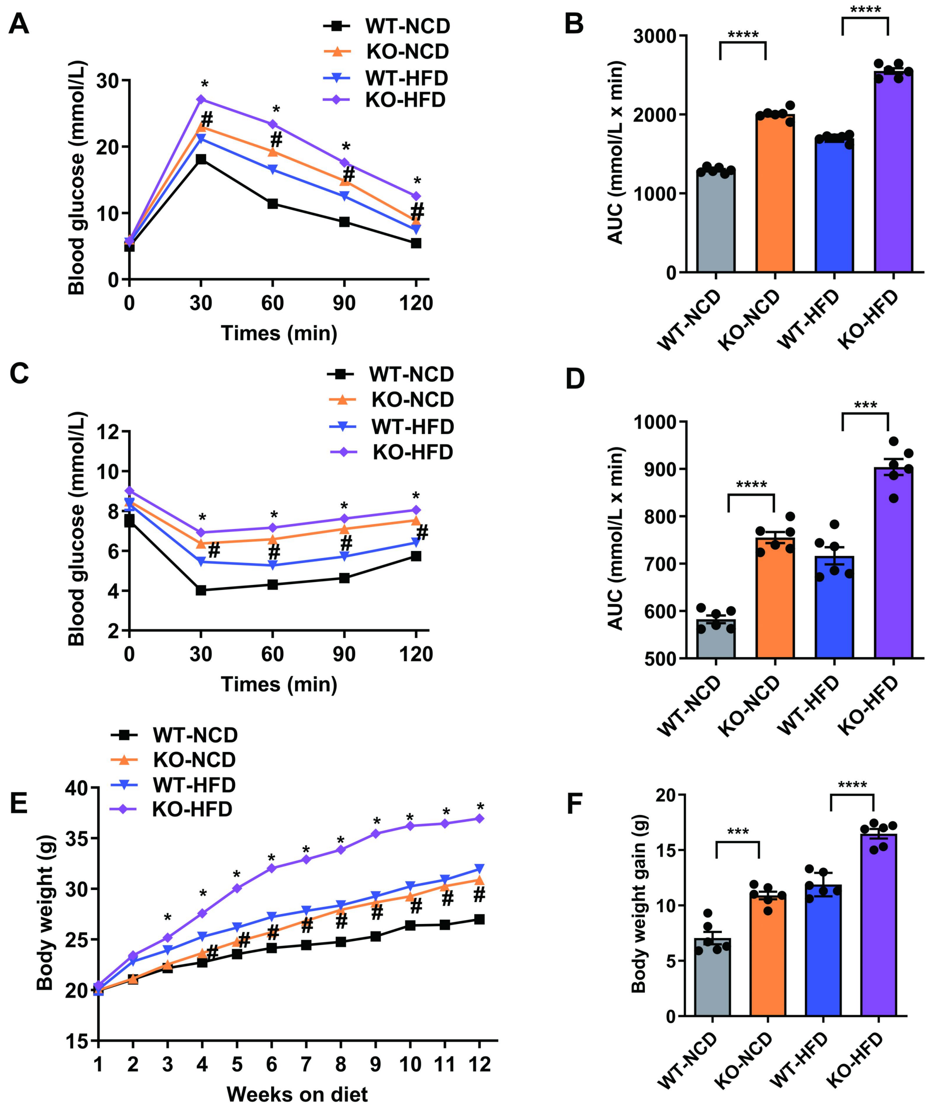


**Supplementary Fig. 3 Myeloid cell specific MYDGF deficiency deteriorated the metabolic profiles of NAFLD mice.** KO and WT mice aged 4-6 weeks were fed with NCD or HFD for 12 weeks (6 mice in each group). **A** The results of the GTT at end of the experiment. $^{\#}P < 0.001$ vs. WT-NCD group. $^{*}P < 0.001$ vs. WT-HFD group. **B** The area under the curve (AUC) from the results of the GTT (n = 6). $^{***}P < 0.001$, $^{****}P < 0.0001$. **C** The results of ITT at the end of the experiment. $^{\#}P < 0.001$ vs. WT-NCD group. $^{*}P < 0.001$ vs. WT-HFD group. **D** The AUC from the results of the ITT (n = 6). $^{***}P < 0.001$, $^{****}P < 0.0001$. **E** Body weight. $^{\#}P < 0.001$ vs. WT-NCD group. $^{*}P <$

0.001 vs. WT-HFD group. **F** Body weight gain. $^{***}P < 0.001$, $^{****}P < 0.0001$. Data are presented as the mean ± SEM. Significant differences were determined by Student's *t*-test or One-way ANOVA followed by Tukey's post-test.

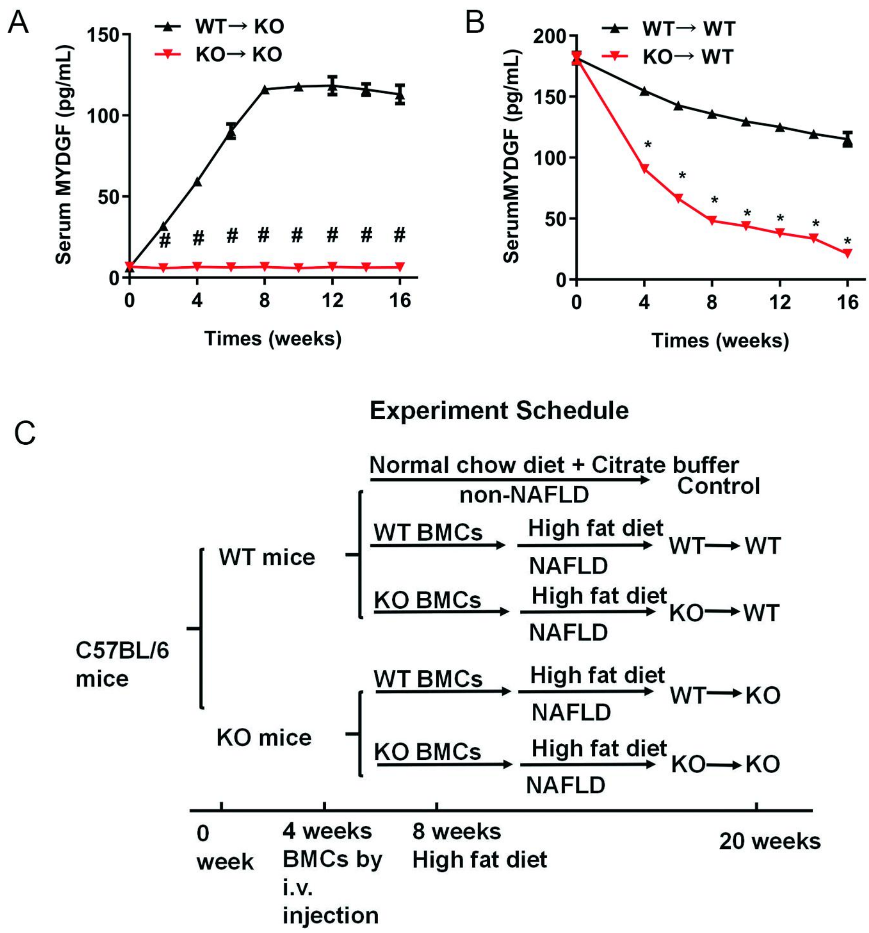


**Supplementary Fig. 4 The expressions of MYDGF after BMT in mice.** BMT was performed in WT and KO mice aged 4-6 weeks and then fed a HFD for 12 weeks. Control group indicates WT mice fed with NCD. Other groups were fed with HFD. **A** Serum levels of MYDGF in KO mice at different time point after BMT (n = 6). **B** Serum levels of MYDGF in WT mice at different time point after BMT (n = 6). **C** The experimental schedule used to assess the effects of BMT on NAFLD. Data are presented as mean ± SEM. $^{\#}P < 0.001$ vs. WT→ KO group. $^{*}P < 0.001$ vs.WT→WT group. Each experiment was repeated 6 times. Data are presented as mean ± SEM. $^{****}P < 0.0001$. Significant differences were determined by Student's *t*-test or One-way ANOVA followed by Tukey's post-test.

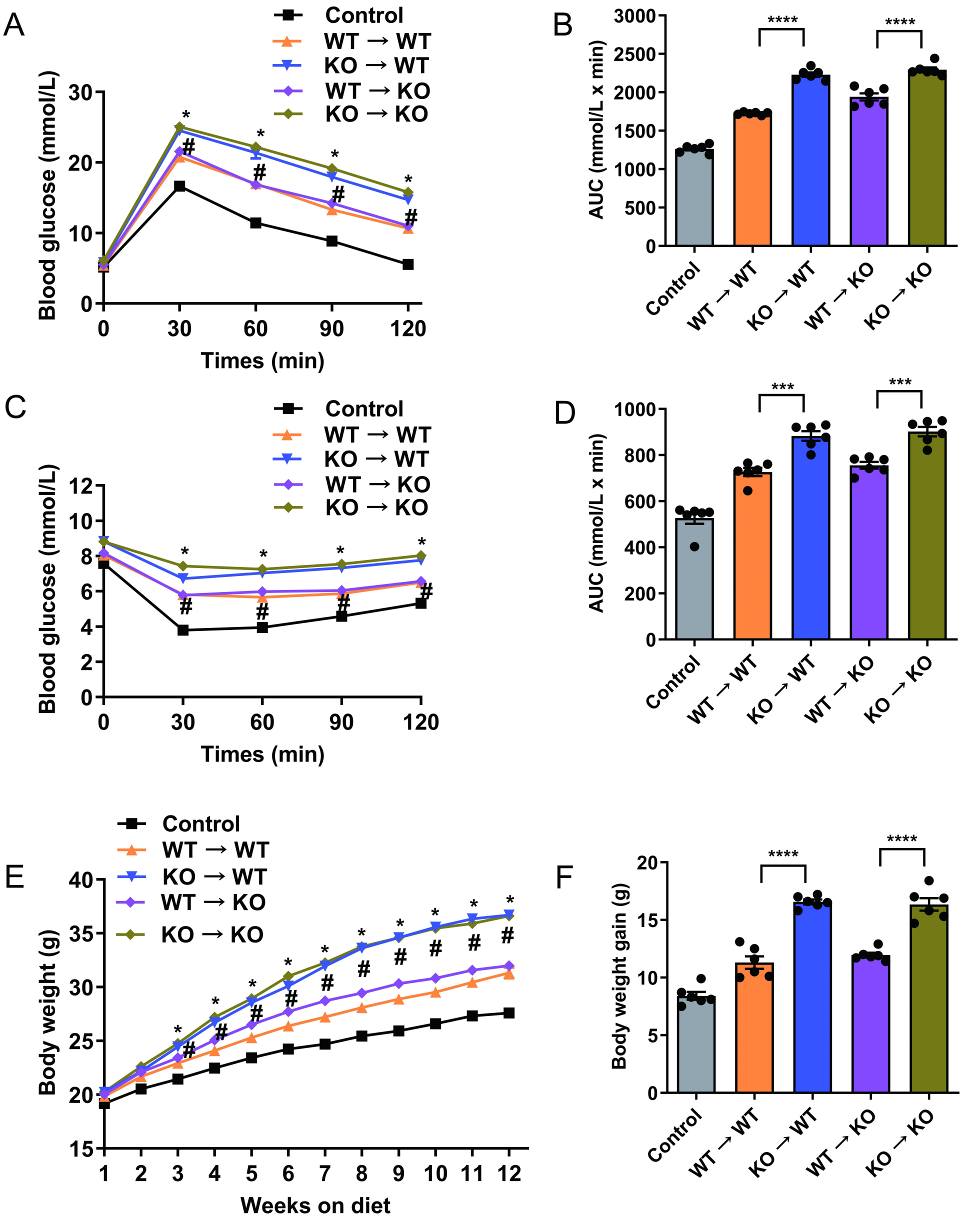


**Supplementary Fig. 5 BMT improved metabolic profiles in NAFLD mice.** BMT was performed in KO and WT mice aged 4-6 weeks and then fed a HFD for 12 weeks as indicated in Supplementary Fig. 4c. **A** The results of the GTT at end of the experiment. $^{\#}P < 0.001$ vs. WT → WT group. $^{*}P < 0.001$ vs. WT → KO group. **B** The AUC from the results of the GTT (n = 6). $^{***}P < 0.001$, $^{****}P < 0.0001$. **C** The results of ITT at the end of the experiment. $^{\#}P < 0.001$ vs. WT → WT group. $^{*}P < 0.001$ vs. WT → KO group. **D** The AUC from the results of the ITT (n = 6). $^{***}P < 0.001$, $^{****}P$

< 0.0001. **E** Body weight. $^{\#}P < 0.001$ vs. WT → WT group. $^{*}P < 0.001$ vs. WT → KO group. **F** Body weight gain (n = 6). $^{***}P < 0.001$, $^{****}P < 0.0001$. Data are presented as the mean ± SEM. Significant differences were determined by Student's *t*-test or One-way ANOVA followed by Tukey's post-test.

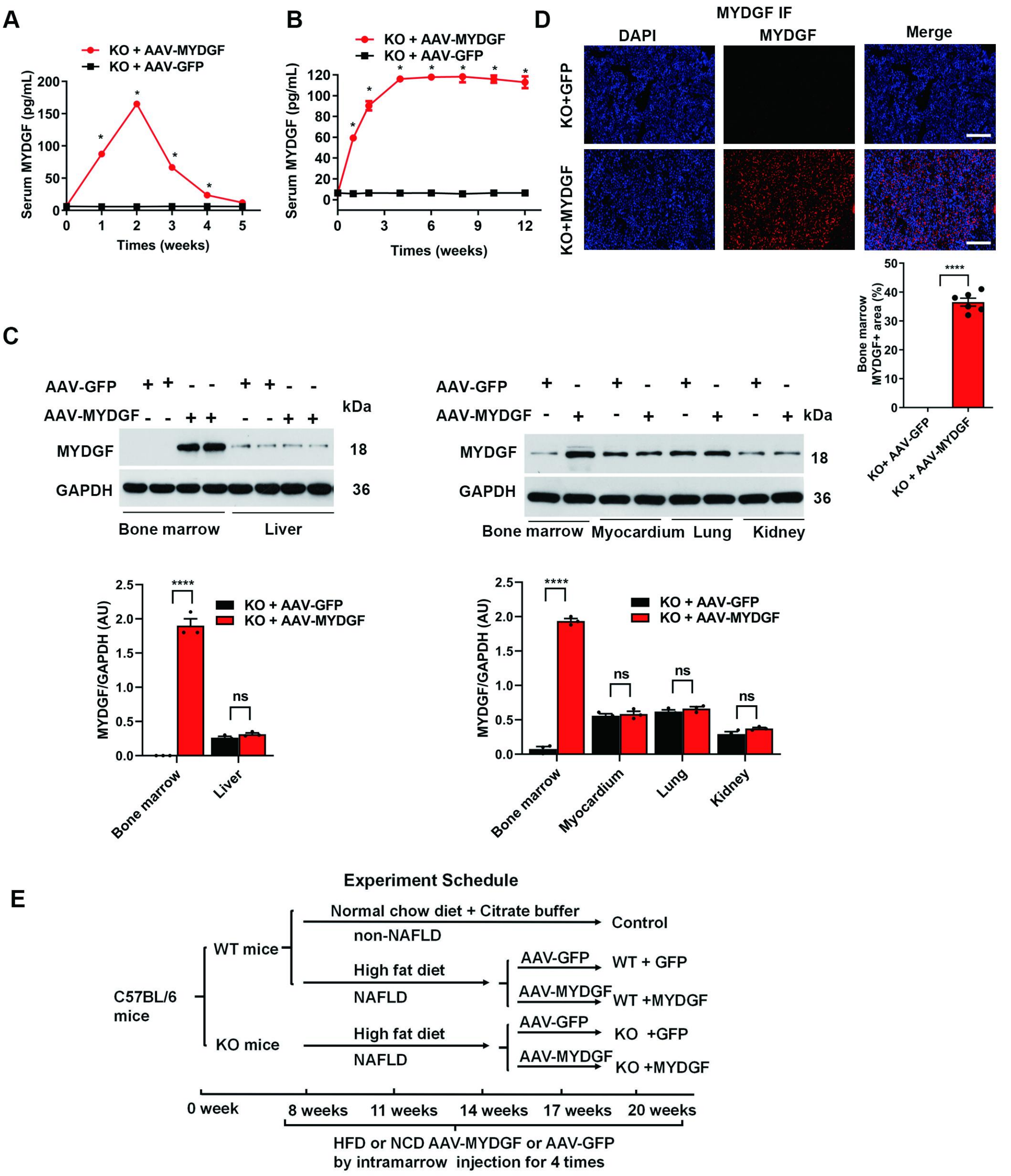


**Supplementary Fig. 6 The expressions of MYDGF after intramarrow injection of AAV-MYDGF in mice. (A-D)** KO and WT mice aged 4-6 weeks were selected and intramarrow AAV-MYDGF injection was done. All mice were fed with NCD. **A** Serum levels of MYDGF at different time point after a single injection of AAV (n = 6). $^{*}P < 0.001$ vs. KO +AAV-GFP group. **B** Serum levels of MYDGF at different time point after injection of AAV every 3 weeks (8 weeks, 11 weeks, 14 weeks, 17 weeks)

(n = 6). $^{*}P < 0.001$ vs. KO +AAV-GFP group. **C** MYDGF protein levels in bone marrow, liver, myocardium, lung and kidney in mice at 12 week (n = 3). $^{****}P < 0.001$. **D** The expression of MYDGF (red) in bone marrow at 12 week (n = 3). White scale bar = 20 μm. $^{****}P < 0.0001$. **E** The experimental schedule used to assess the effects of MYDGF expression in bone marrow on NAFLD after intramarrow AAV injection (n = 6). Control group was fed with NCD, and others were fed with HFD for 12 weeks. Data are presented as the mean ± SEM. Significant differences were determined by Student's *t*-test or One-way ANOVA followed by Tukey's post-test.

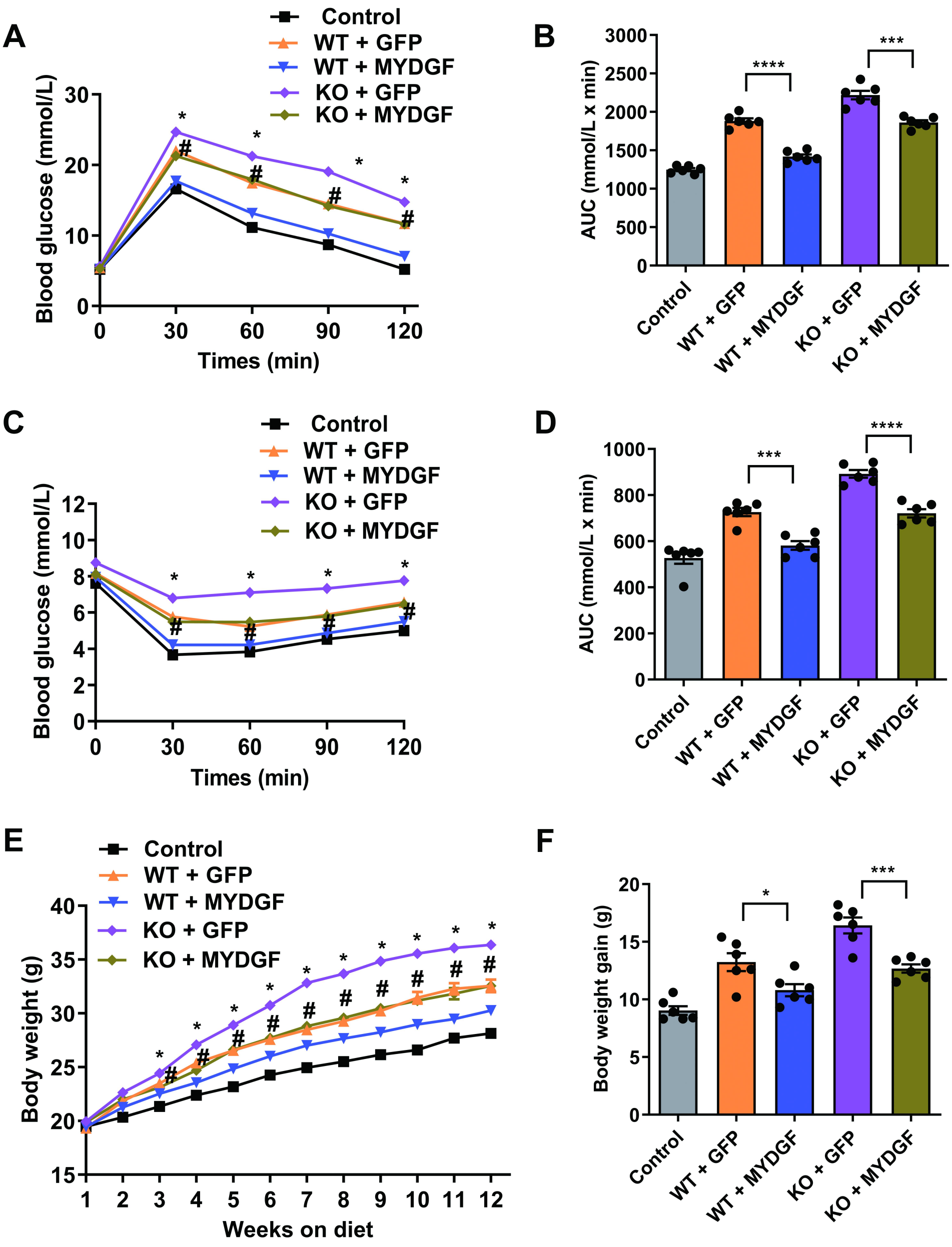


**Supplementary Fig. 7 Bone marrow-specific overexpressions of MYDGF improved metabolic profiles in NAFLD mice.** KO and WT mice aged 4-6 weeks were selected and intramarrow AAV-MYDGF injection was done as indicated in Supplementary Fig. 6e. Control group was fed a NCD, and others were fed a HFD for 12 weeks. **A** The results of the GTT at end of the experiment. $^{\#}P < 0.001$ vs. WT-GFP group. $^{*}P < 0.001$ vs. KO-GFP group. **B** The AUC from the results of the GTT (n = 6). $^{***}P < 0.001$, $^{****}P < 0.0001$. **C** The results of ITT at the end of the experiment. $^{\#}P <$

0.001 vs. WT-GFP group. $^{*}P < 0.001$ vs. KO-GFP group. **D** The AUC from the results of the ITT (n = 6). $^{***}P < 0.001$, $^{****}P < 0.0001$. **E** Body weight. $^{\#}P < 0.001$ vs. WT-GFP group. $^{*}P < 0.001$ vs. KO-GFP group. **F** Body weight gain (n = 6). $^{*}P < 0.05$, $^{***}P < 0.001$. Data are presented as the mean ± SEM. Significant differences were determined by Student's *t*-test or One-way ANOVA followed by Tukey's post-test.

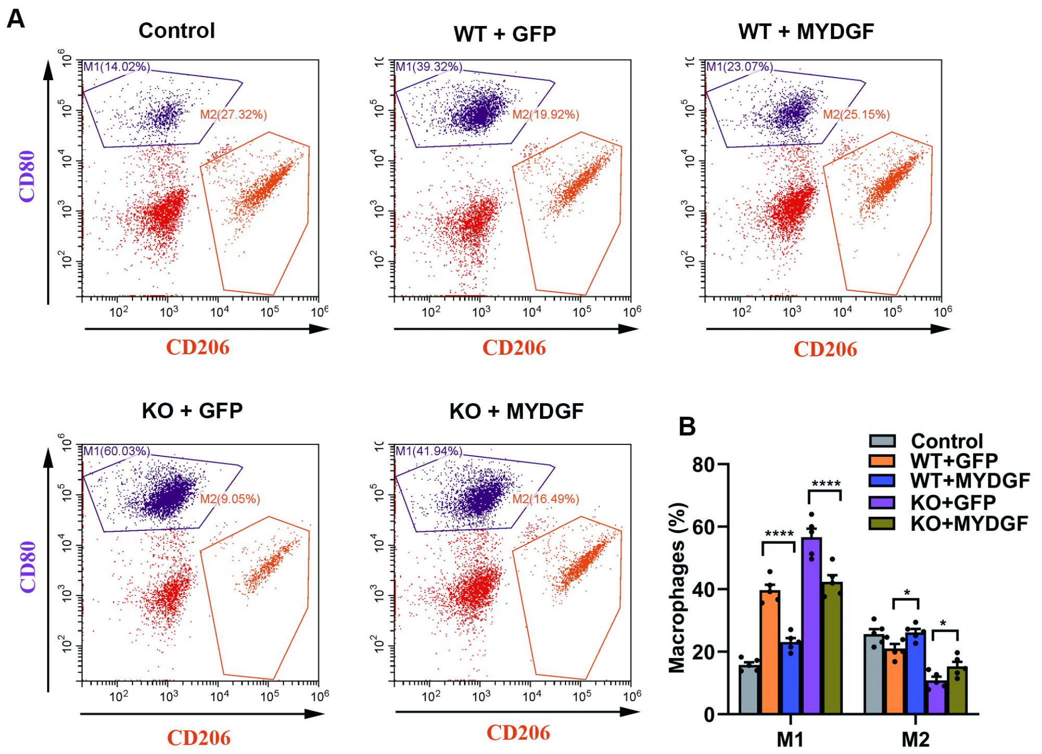


**Supplementary Fig. 8 MYDGF inhibited the polarization of macrophages M1 and promoted the polarization of macrophages M2.** Mice were fed as described in Fig. S6E. KCs were isolated from liver of different groups of mice and analyzed for F4/80 expression (maturate macrophages). **A** Mature macrophages were further analyzed for CD80 and CD206 expression by flow cytometry. Each experiment was repeated 5 times. **B** Quantification of the results of **A**. Data are presented as mean ± SEM. $^{*}P < 0.05$, $^{**}P < 0.01$, $^{***}P < 0.001$, $^{****}P < 0.0001$. Significant differences were determined by Student's *t*-test or One-way ANOVA followed by Tukey's post-test.

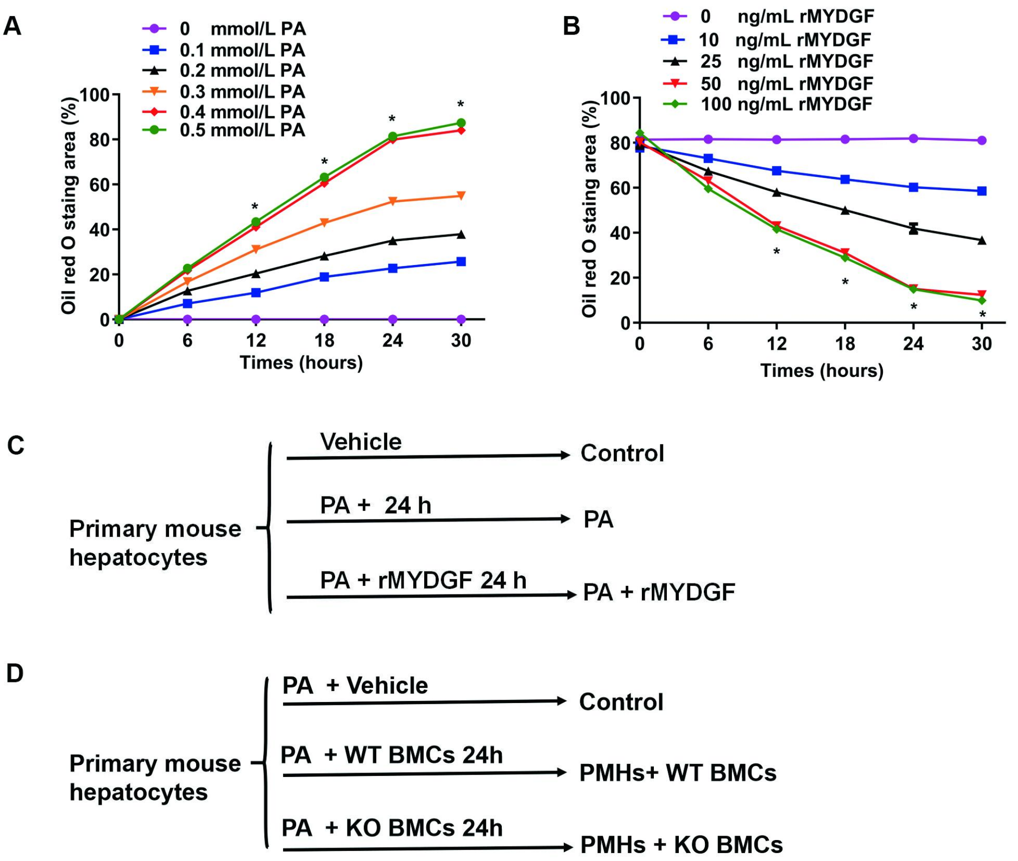


**Supplementary Fig. 9 Identification of the optimum conditions for the incubation of PMHs with PA and rMYDGF. A** Time- and dose-dependent PA-induced changes in fat deposition determined by oil red O staining. **B** Time- and dose-dependent PA-induced changes in fat deposition following rMYDGF incubation determined by oil red O staining. **(C-D)** The experimental schedule used to assess the effects of MYDGF on PA-induced PMHs treated with rMYDGF (**C**) or cocultured with BMCs (**D**). Each experiment was repeated six times. Data are presented as the mean ± SEM. $^{*}P$ <0.001 vs. control group. Significant differences were determined by Student's *t*-test or One-way ANOVA followed by Tukey's post-test.

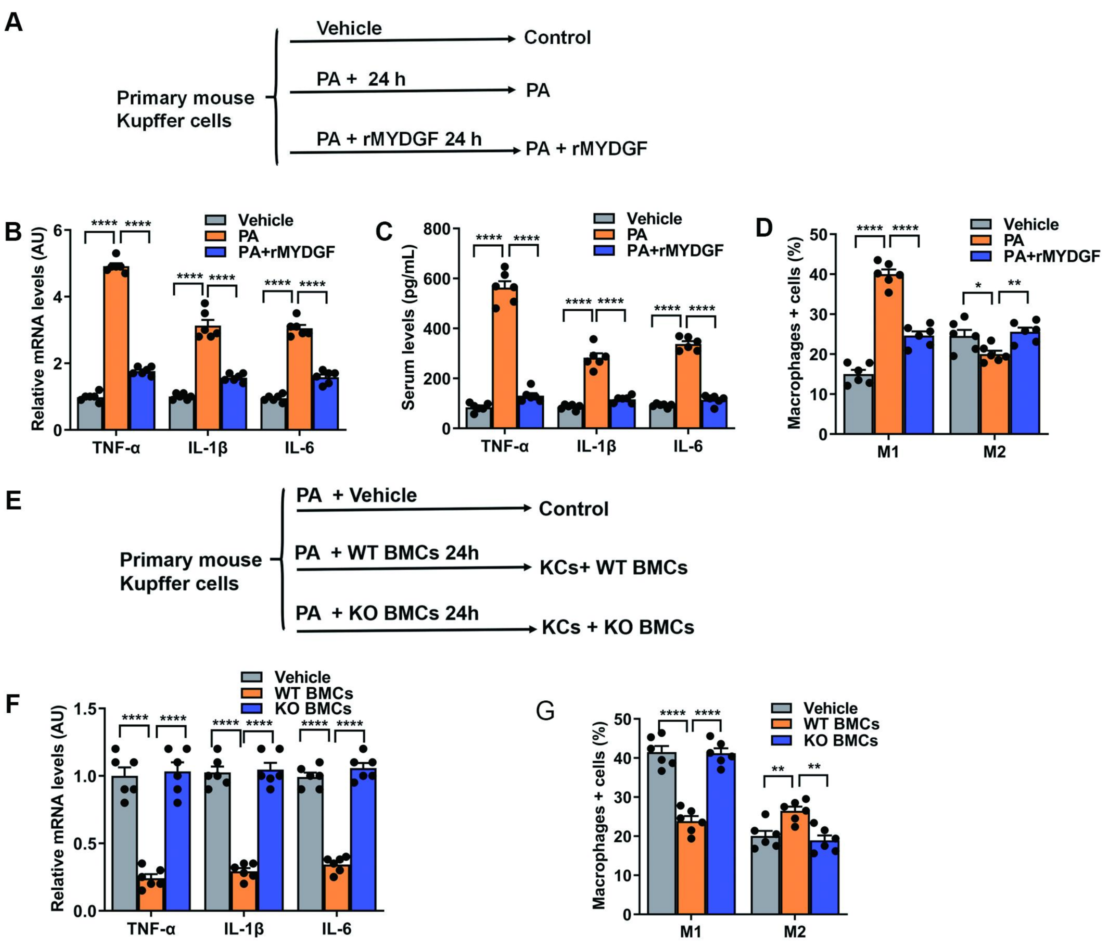


**Supplementary Fig. 10 MYDGF improved inflammation, and promoted the polarization of macrophages M2 in KCs. (A-D)** The effects of MYDGF on inflammation and polarization of macrophages in KCs. **A** The experimental schedule used to assess the effects of MYDGF on PA-induced KCs treated with rMYDGF. **B** The levels of inflammatory factors in KCs after rMYDGF treatments. **C** The levels of inflammatory factors in supernatants after rMYDGF treatments. **D** The results of polarization of macrophages after rMYDGF treatment. **(E-G)** The effects of MYDGF on inflammation and polarization of macrophages in coculture experiments. **E** The experimental schedule used to assess the effects of MYDGF on PA-induced KCs cocultured with BMCs. **F** The mRNA levels of TNFα, IL-1β and IL-6 in PMHs cocultured with BMCs. **G** The results of polarization in macrophages cocultured with

BMCs. Each experiment was repeated six times. Data are presented as the mean ± SEM. $^{*}P < 0.05$, $^{**}P < 0.01$, $^{***}P < 0.001$, $^{****}P < 0.0001$. Significant differences were determined by Student's *t*-test or One-way ANOVA followed by Tukey's post-test.

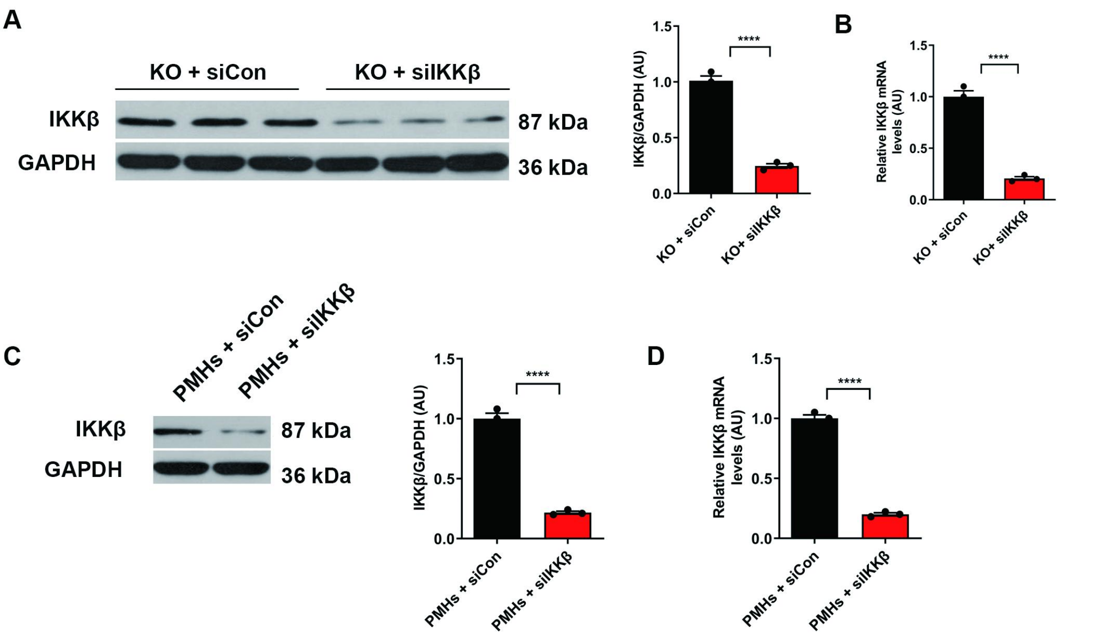


**Supplementary Fig. 11 The efficiency of IKKβ silencing *in vivo* and *in vitro*. (a,b)** KO mice aged 4-6 weeks were treated with siIKKβ and siCon and fed a HFD for 12 weeks. **A** Liver IKKβ protein levels in mice after a single injection of AAV siRNA (n = 3). **B** Liver IKKβ mRNA levels in mice after a single injection of AAV siRNA (n = 3). **(C-D**) PMHs were transfected with siIKKβ and siCon. After 12 hours, another treatment with PA 0.4 mmol/L or rMYDGF 50 ng/mL for 24 hours. **C** IKKβ protein levels in PMHs after transfected siRNA (n = 3). **D** IKKβ mRNA levels in PMHs after transfected siRNA (n = 3). Data are presented as the mean ± SEM. $^{*}P < 0.05$, $^{**}P < 0.01$, $^{***}P < 0.001$, $^{****}P < 0.0001$. Significant differences were determined by Student's *t*-test.

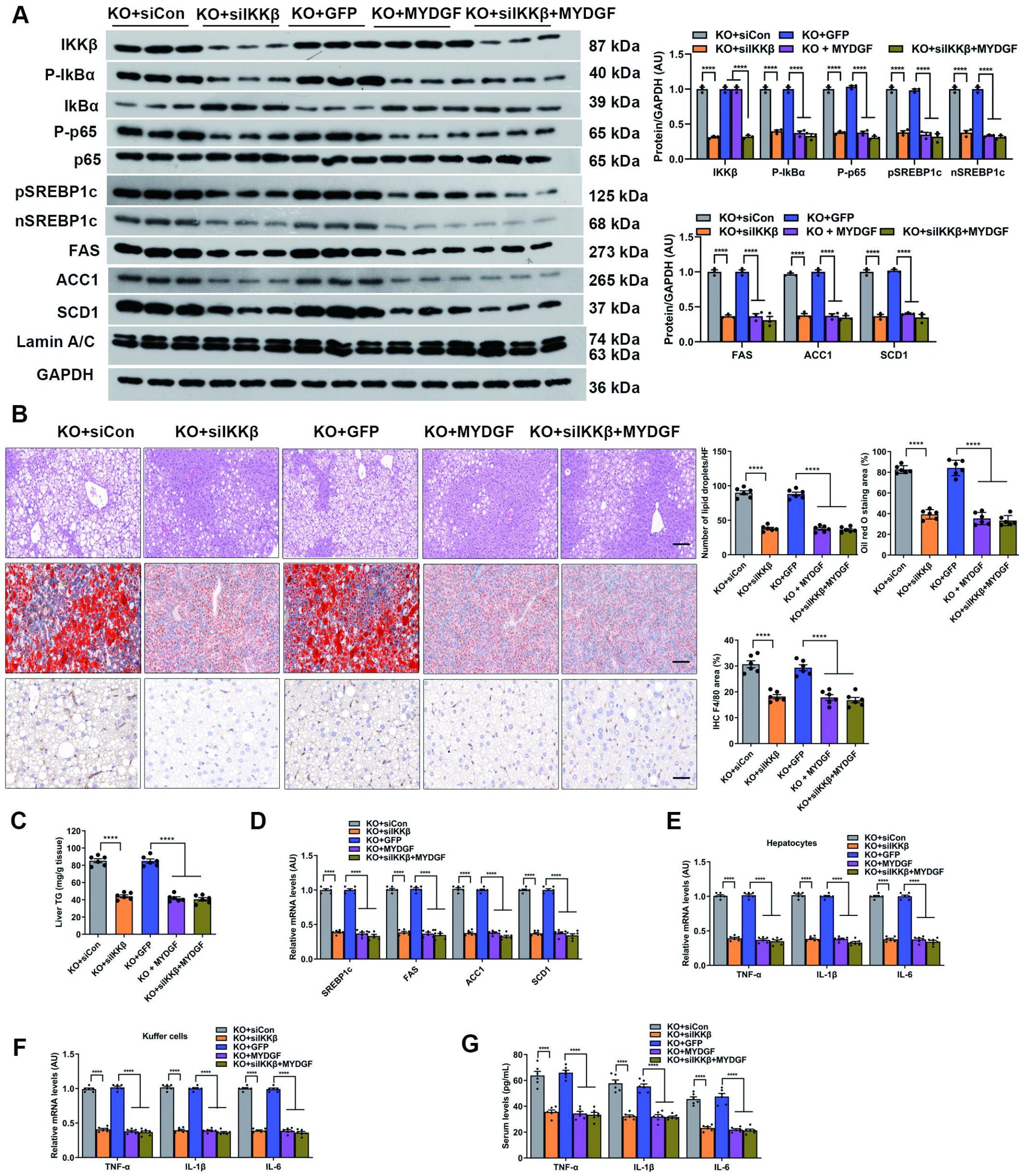


**Supplementary Fig. 12 IKKβ silencing and bone marrow-specific overexpression of MYDGF inhibited NF-κB signaling and lipogenesis and alleviated inflammation and fat deposition *in vivo*.** KO mice aged 4-6 weeks were treated with siIKKβ and AAV-MYDGF and fed a HFD for 12 weeks. **A** The levels of NF-κB signaling and lipogenesis proteins in siIKKβ-transfected and AAV-MYDGF-injected KO livers of mice (n = 3). **B** Representative images showing H&E staining, oil red O

staining and F4/80 staining of liver sections (n = 6). Black scale bar = 50 μm. **C** The hepatic TG content (n = 6). **D** The mRNA levels of genes related to fat metabolism in isolated hepatocytes (n = 6). **E** The levels of inflammatory factors in isolated hepatocytes (n = 6). **F** The levels of inflammatory factors in KCs (n = 6). **G** The levels of inflammatory factors in serum (n = 6). Data are presented as the mean ± SEM. $^{*}P < 0.05$, $^{**}P < 0.01$, $^{***}P < 0.001$, $^{****}P < 0.0001$. Significant differences were determined by Student's *t*-test or One-way ANOVA followed by Tukey's post-test.

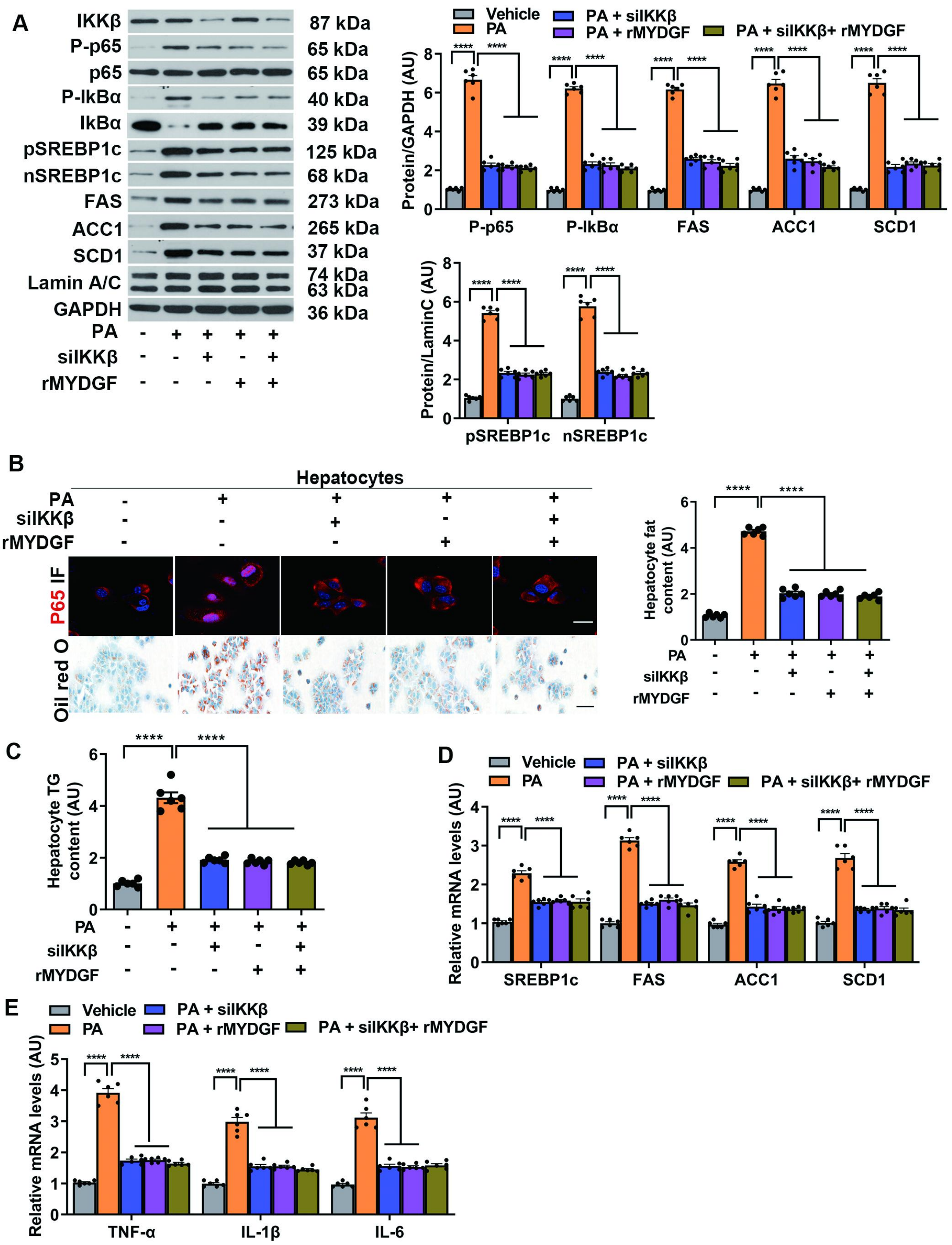


**Supplementary Fig. 13 IKKβ silencing inhibited NF-κB signaling and lipogenesis and alleviated inflammation and fat deposition *in vitro*.** PMHs were transfected with siIKKβ and siCon. After 12 hours, another treatment with PA 0.4 mmol/L or rMYDGF 50 ng/mL for 24 hours. **A** The levels of NF-κB signaling and lipogenesis proteins in siIKKβ-transfected PMHs. **B** p65 nuclear translocation and hepatocyte fat

deposition. White scale bar = 10 μm. Black scale bar = 20 μm. **C** Quantification of TG levels in PMHs. **D** The mRNA levels of genes related to fat metabolism in PMHs. **E** The mRNA levels of TNF-α, IL-1β and IL-6 in PMHs. Each experiment was repeated six times. Data are presented as the mean ± SEM. $^{*}P < 0.05$, $^{**}P < 0.01$, $^{***}P < 0.001$, $^{****}P < 0.0001$. Significant differences were determined by Student's *t*-test or One-way ANOVA followed by Tukey's post-test.

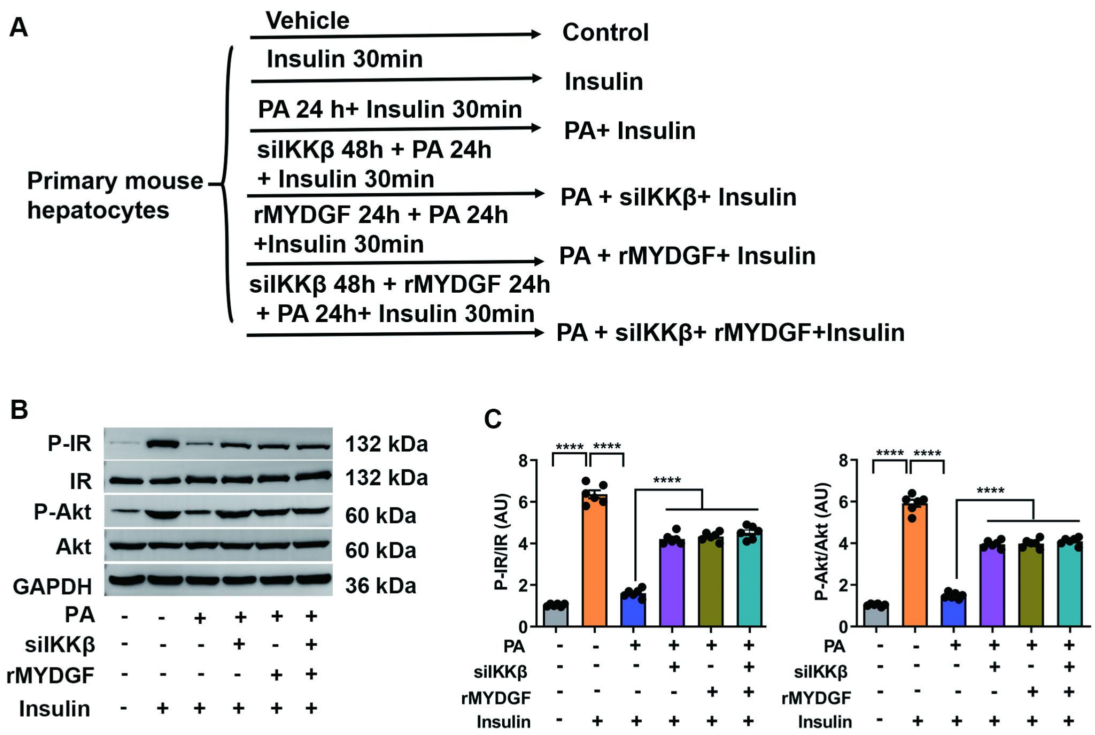


**Supplementary Fig. 14 IKKβ silencing improved insulin signaling in PA-induced PMHs. A** Experimental schedule used to assess the effects of MYDGF on PA-induced PMHs treated with insulin. **B** Levels of insulin signaling proteins in PMHs of different treatment groups. **C** Quantitative analysis of **B**. Each experiment was repeated six times. Data are presented as the mean ± SEM. $^{*}P < 0.05$, $^{**}P < 0.01$, $^{***}P < 0.001$, $^{****}P < 0.0001$. Significant differences were determined by Student's t-test or one-way ANOVA followed by Tukey's post-test.
