# Supplementary material for: Myeloid-derived growth factor alleviates non-alcoholic fatty liver disease alleviates in a manner involving IKKβ/NF-κB signaling
Source: Cell Death Dis. 2023 Jun 26;14(6):376. doi: 10.1038/s41419-023-05904-y (PMC10293205; doi:10.1038/s41419-023-05904-y)

Full unedited blot for Figure 4I

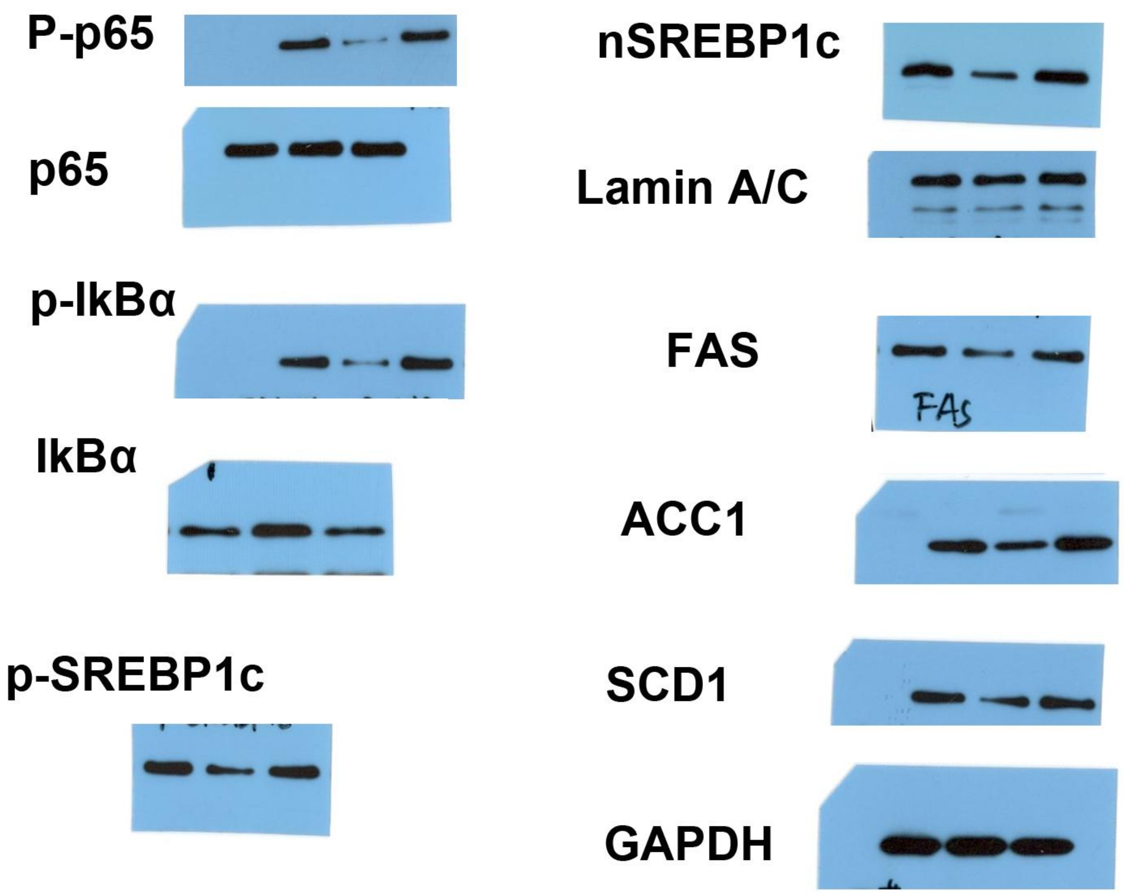

Full unedited blot for Figure 5A

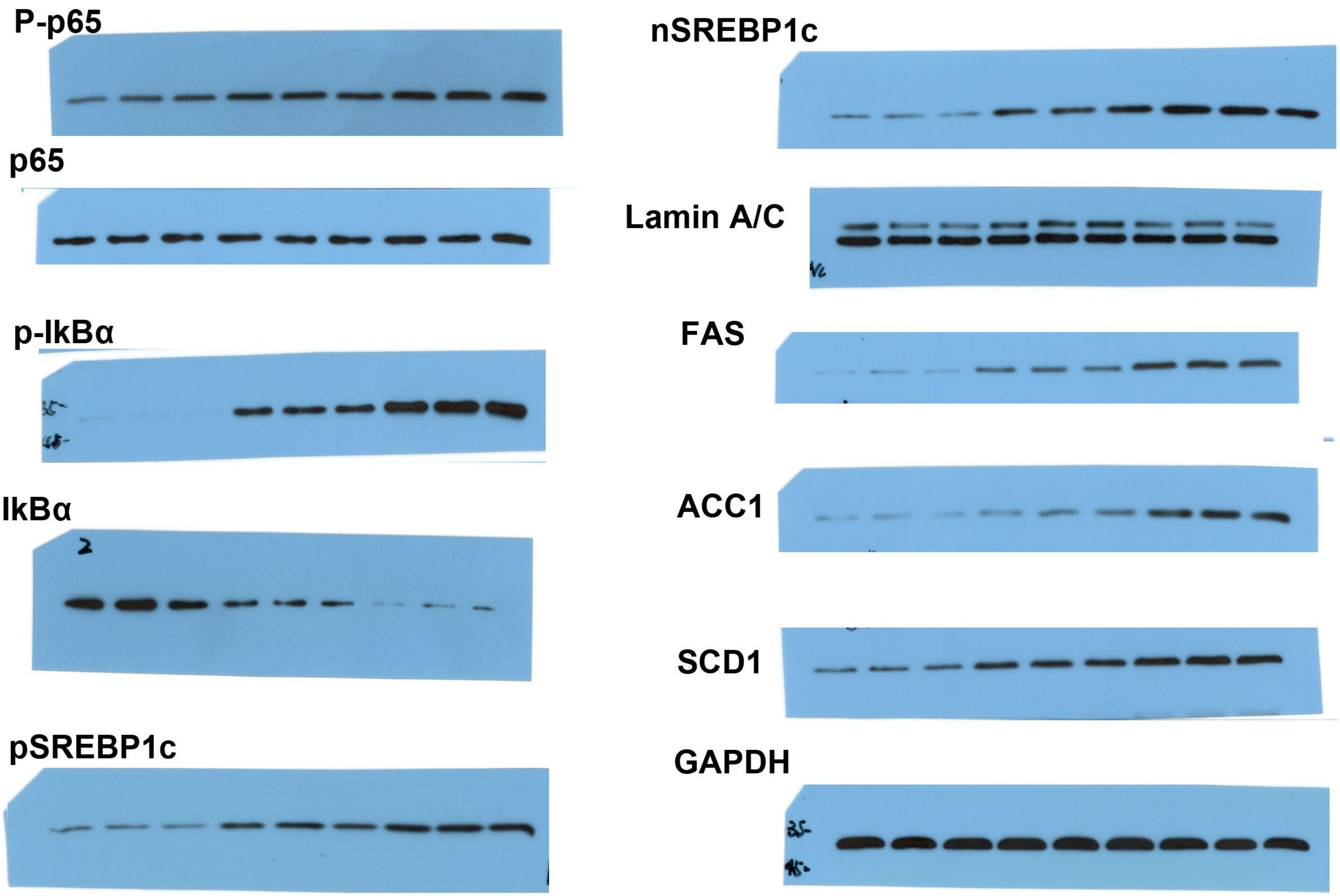

Full unedited blot for Figure 5B

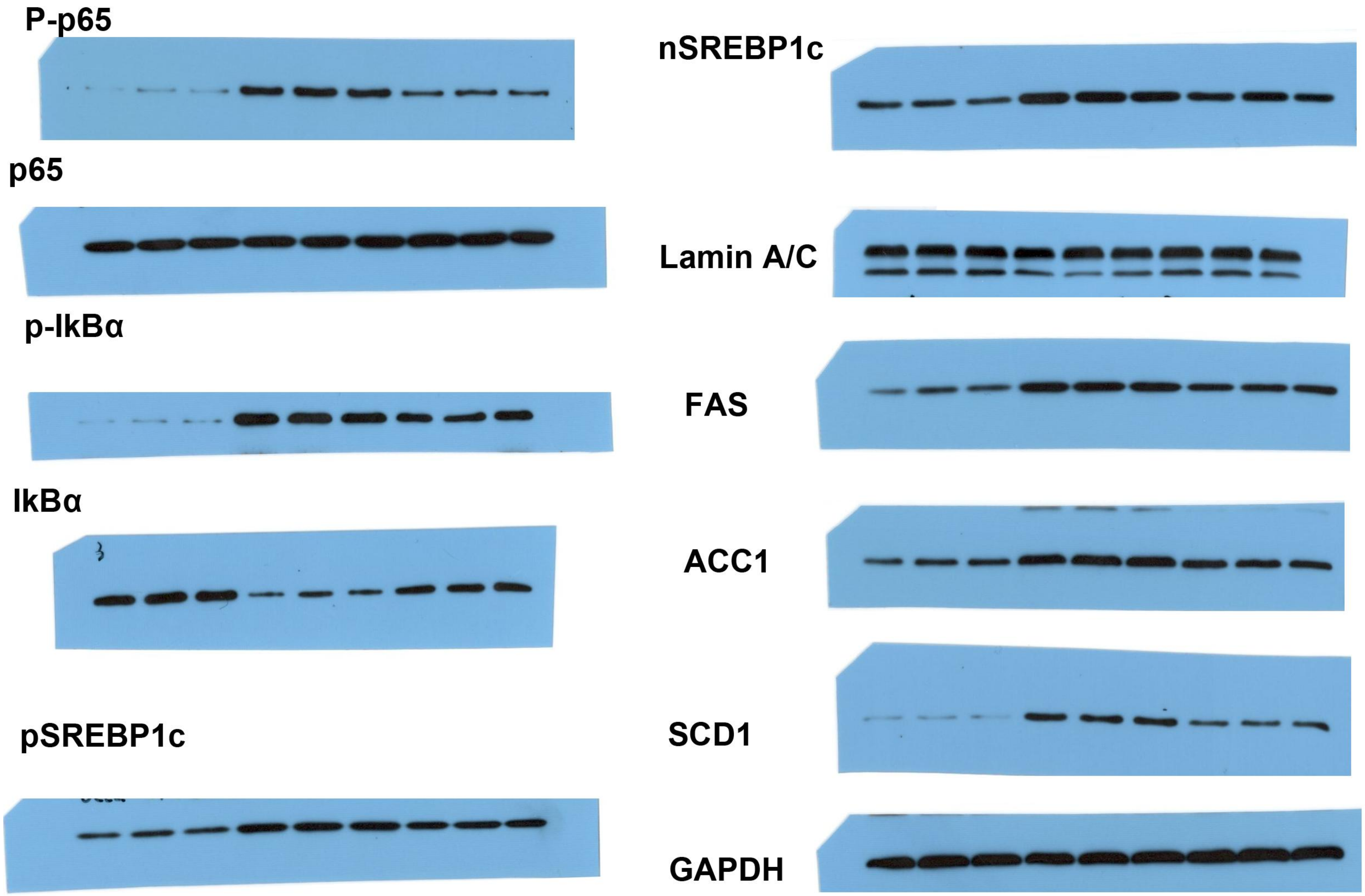

Full unedited blot for Figure 5C

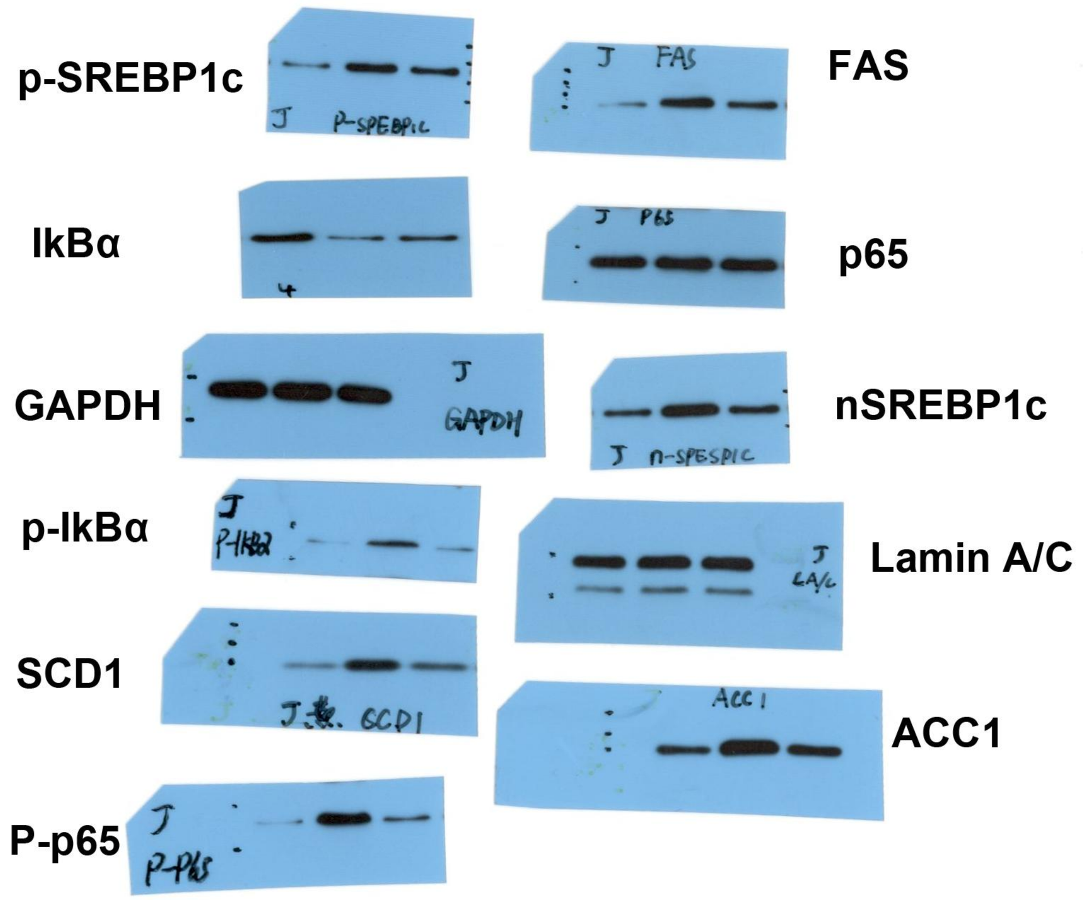

Full unedited blot for Figure 5D

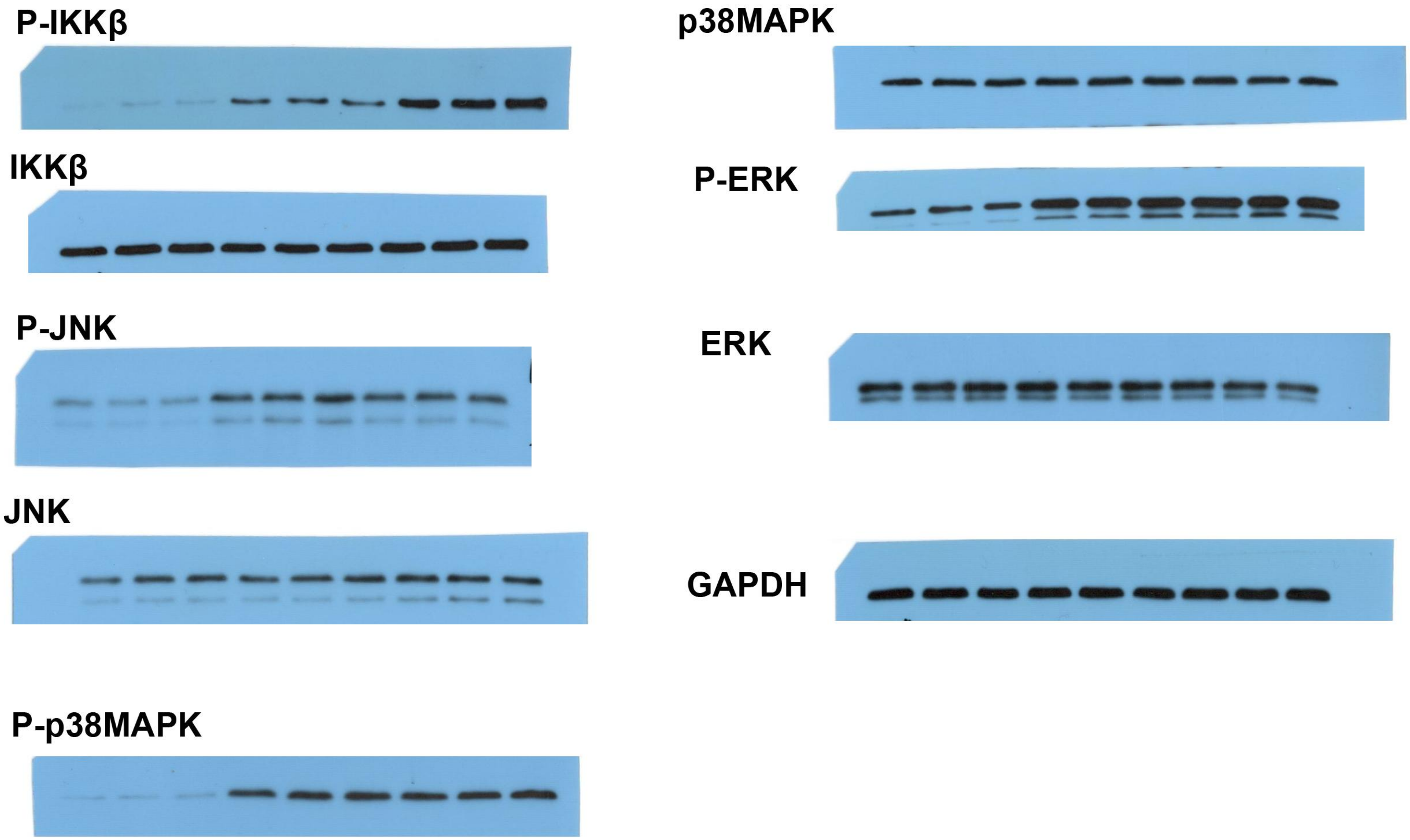

Full unedited blot for Figure 5E

P-IKKβ

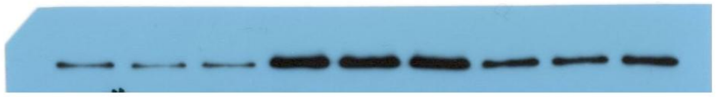

IKKβ

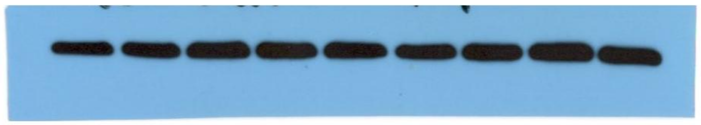

P-JNK

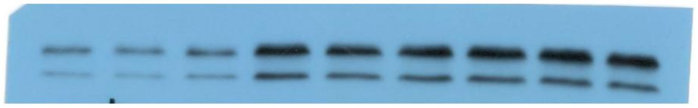

JNK

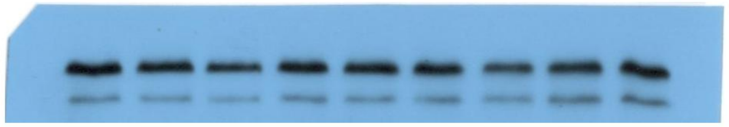

P-p38MAPK

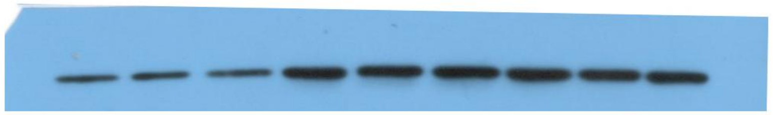

p38MAPK

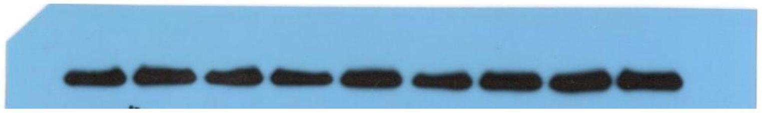

P-ERK

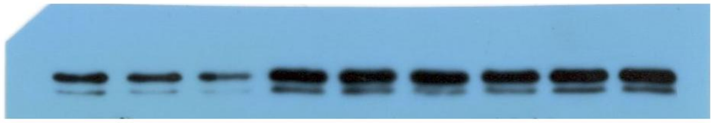

ERK

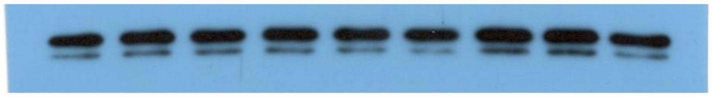

GAPDH

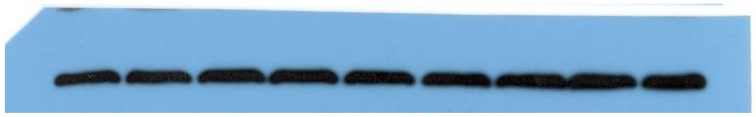

Full unedited blot for Figure 5F

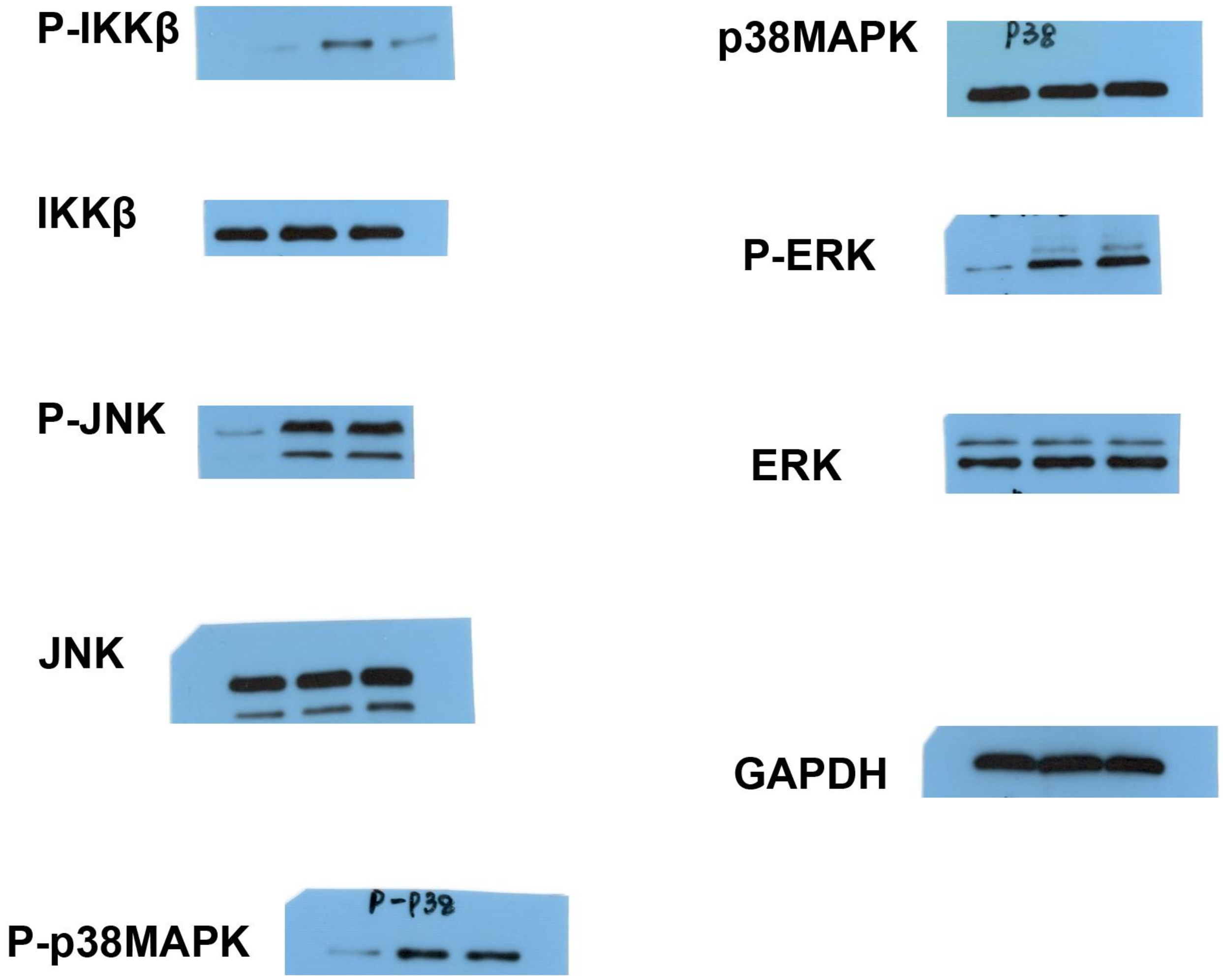

Full unedited blot for Figure 6A

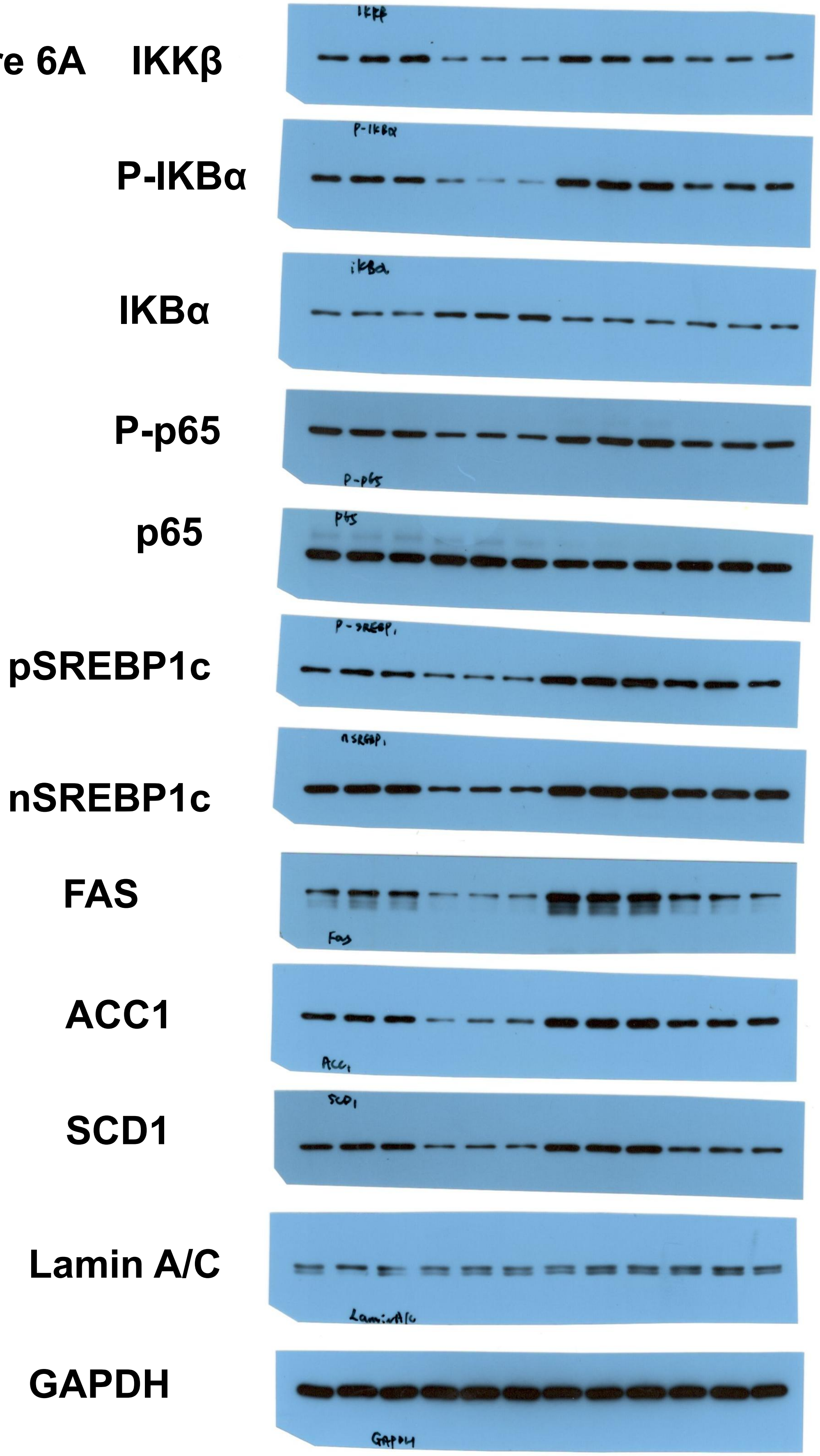

Full unedited blot for Figure 7C

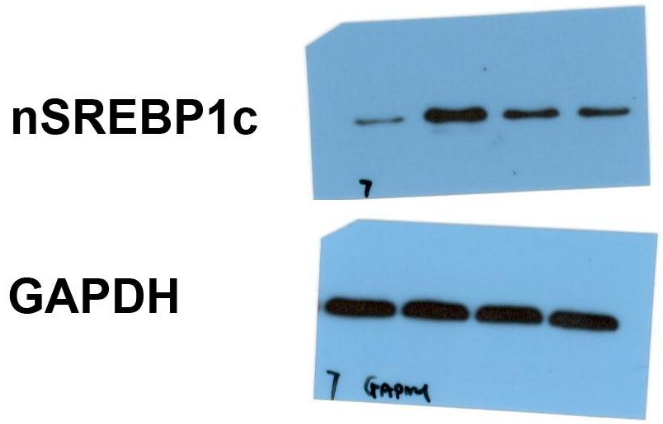

Full unedited blot for Figure 8A

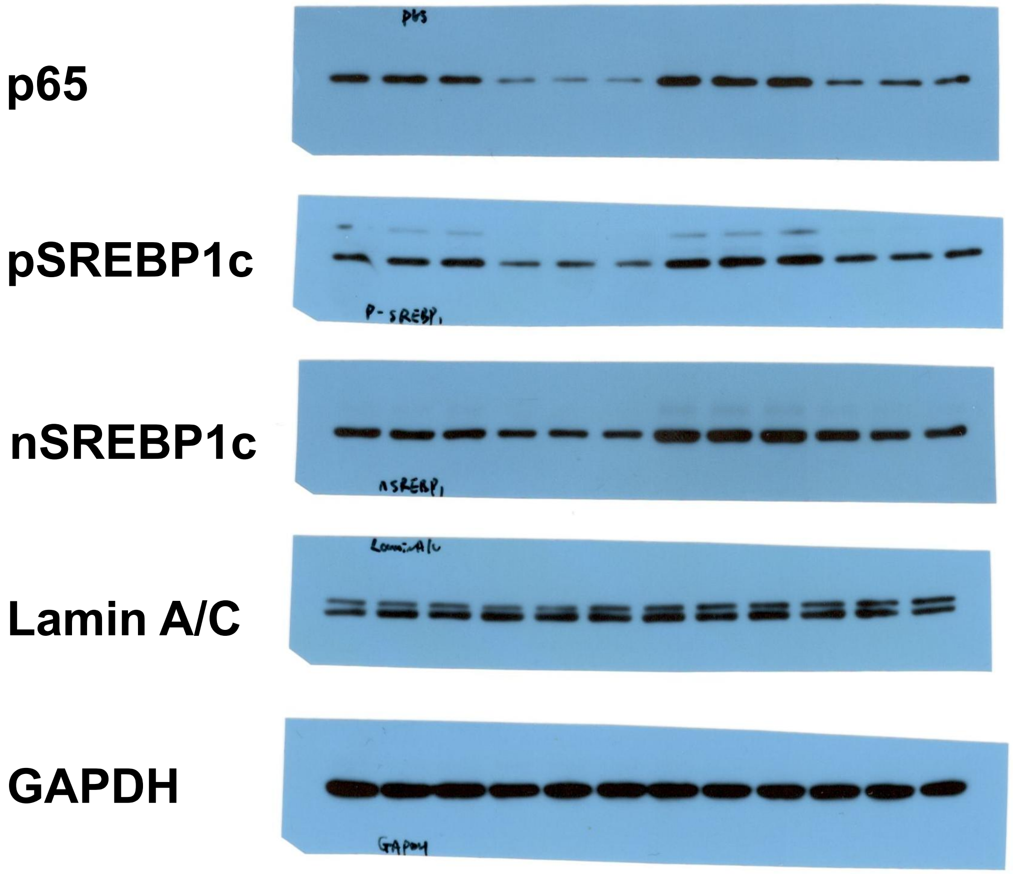

Full unedited blot for Figure 9A

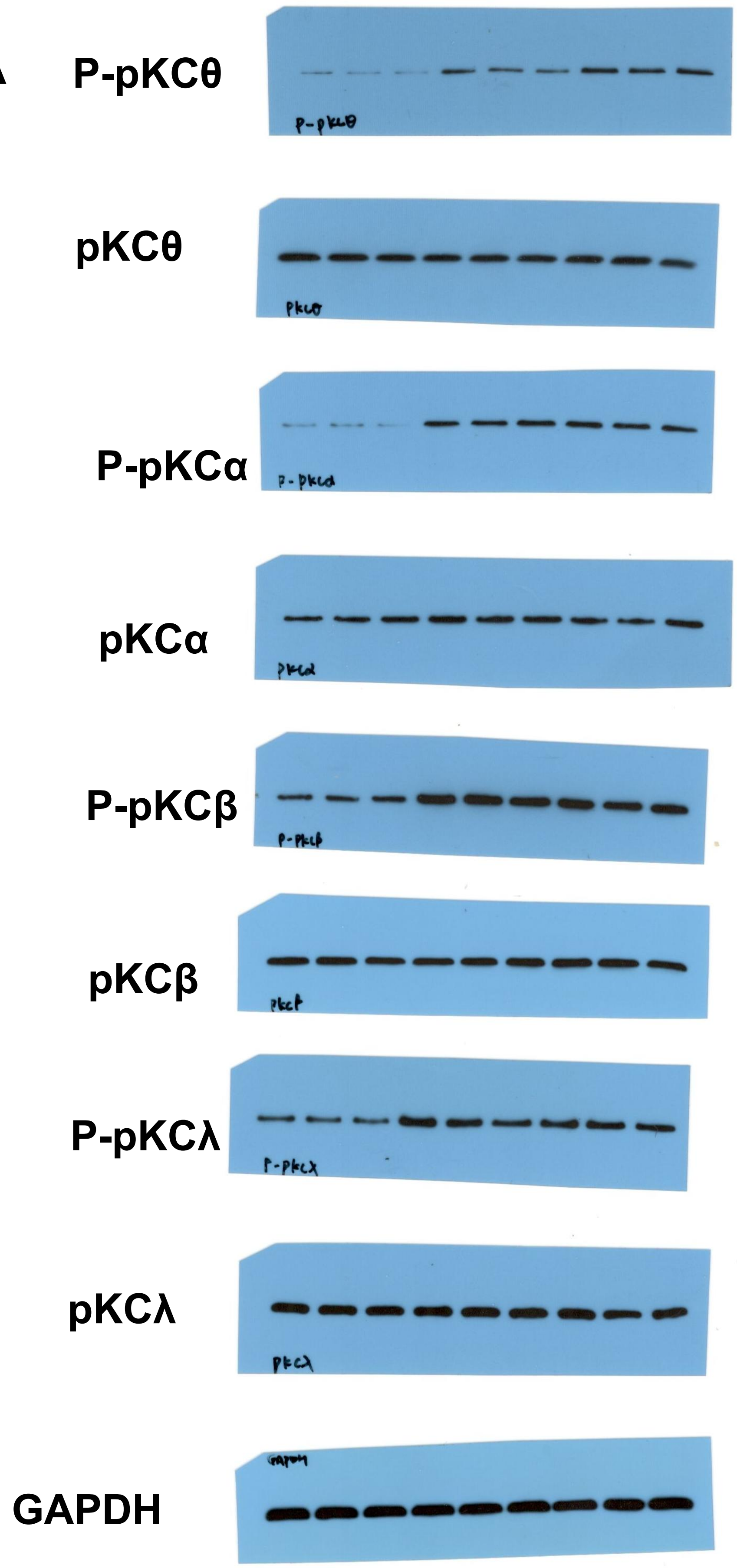

Full unedited blot for Figure 9B

pKCθ

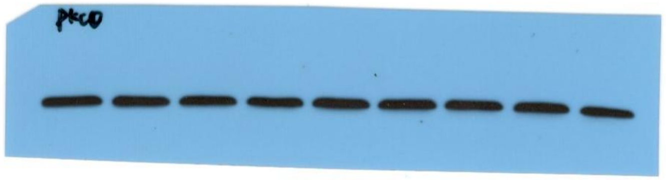

P-pKCθ

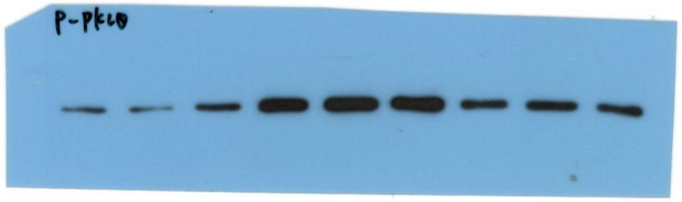

GAPDH

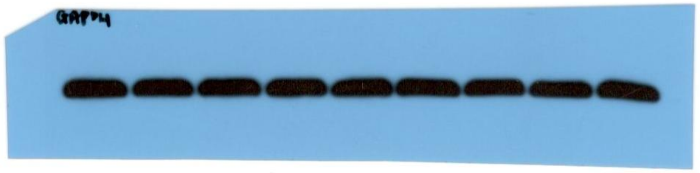

Full unedited blot for Figure 9C

P-IKKβ

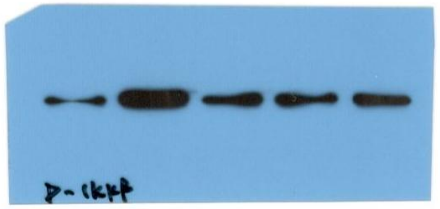

IKKβ

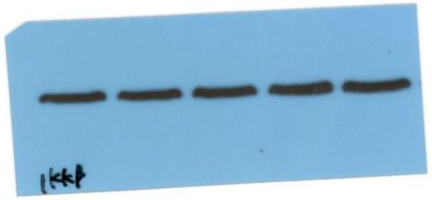

P-IKBα

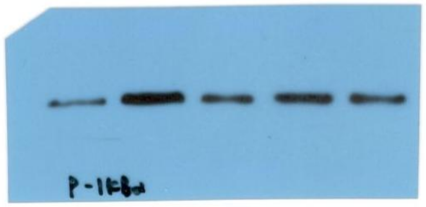

IKBα

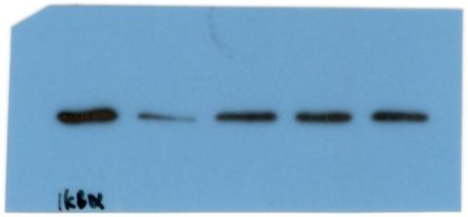

GAPDH

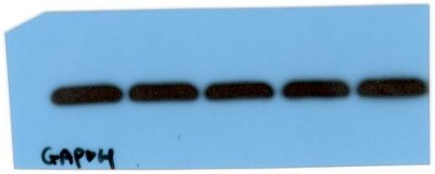

Full unedited blot for FigureS 1C

MYDGF

GAPDH

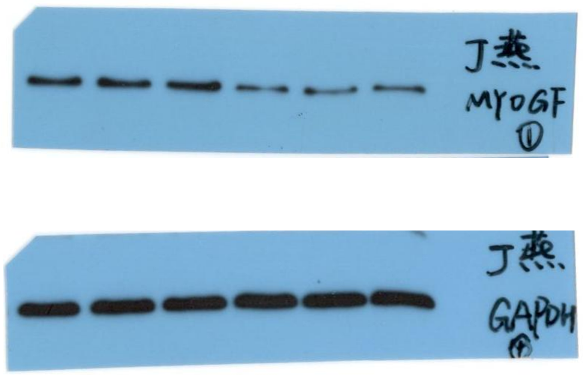

Full unedited blot for FigureS 2C

MYDGF

GAPDH

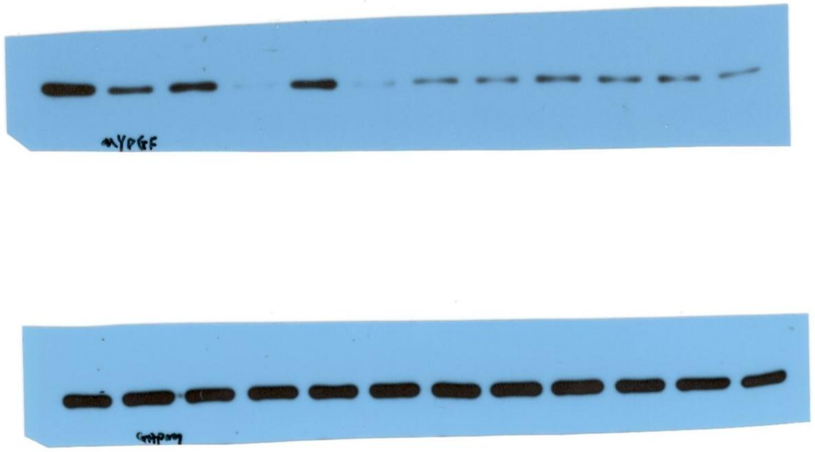

Full unedited blot for FigureS 2D

MYDGF

GAPDH

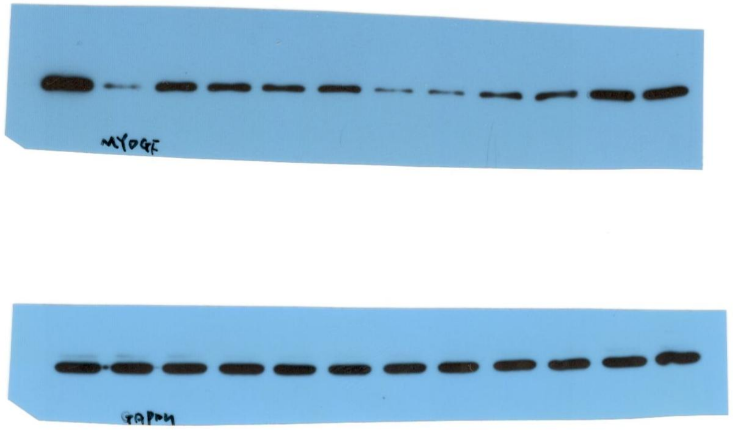

Full unedited blot for FigureS 6C

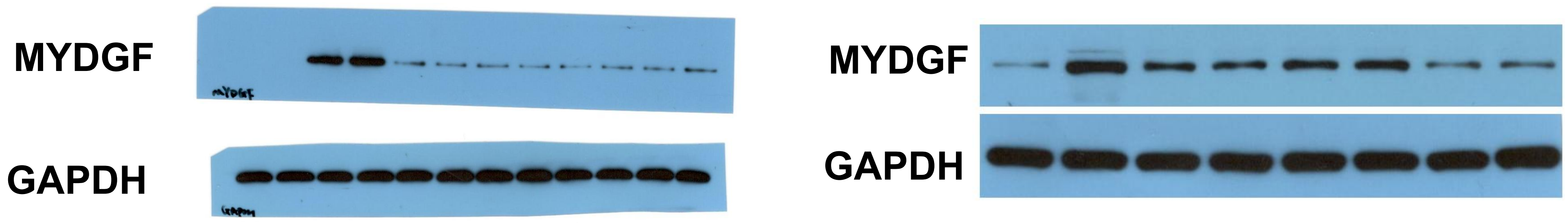

Full unedited blot for FigureS 11A

IKKβ

GAPDH

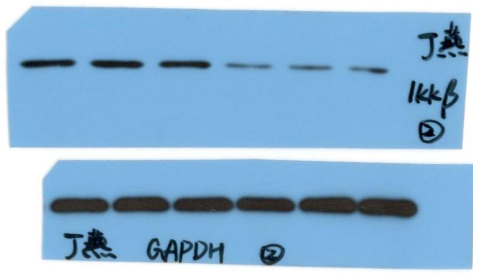

Full unedited blot for FigureS 11C

IKKβ

GAPDH

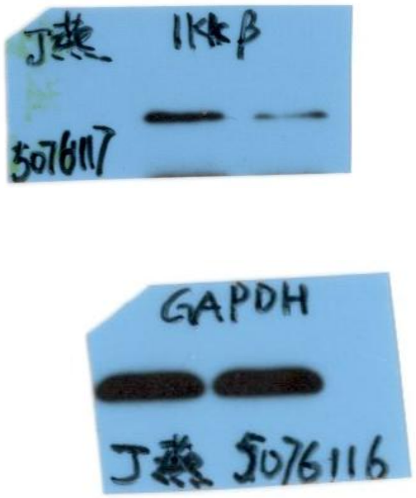

Full unedited blot for FigureS 12A

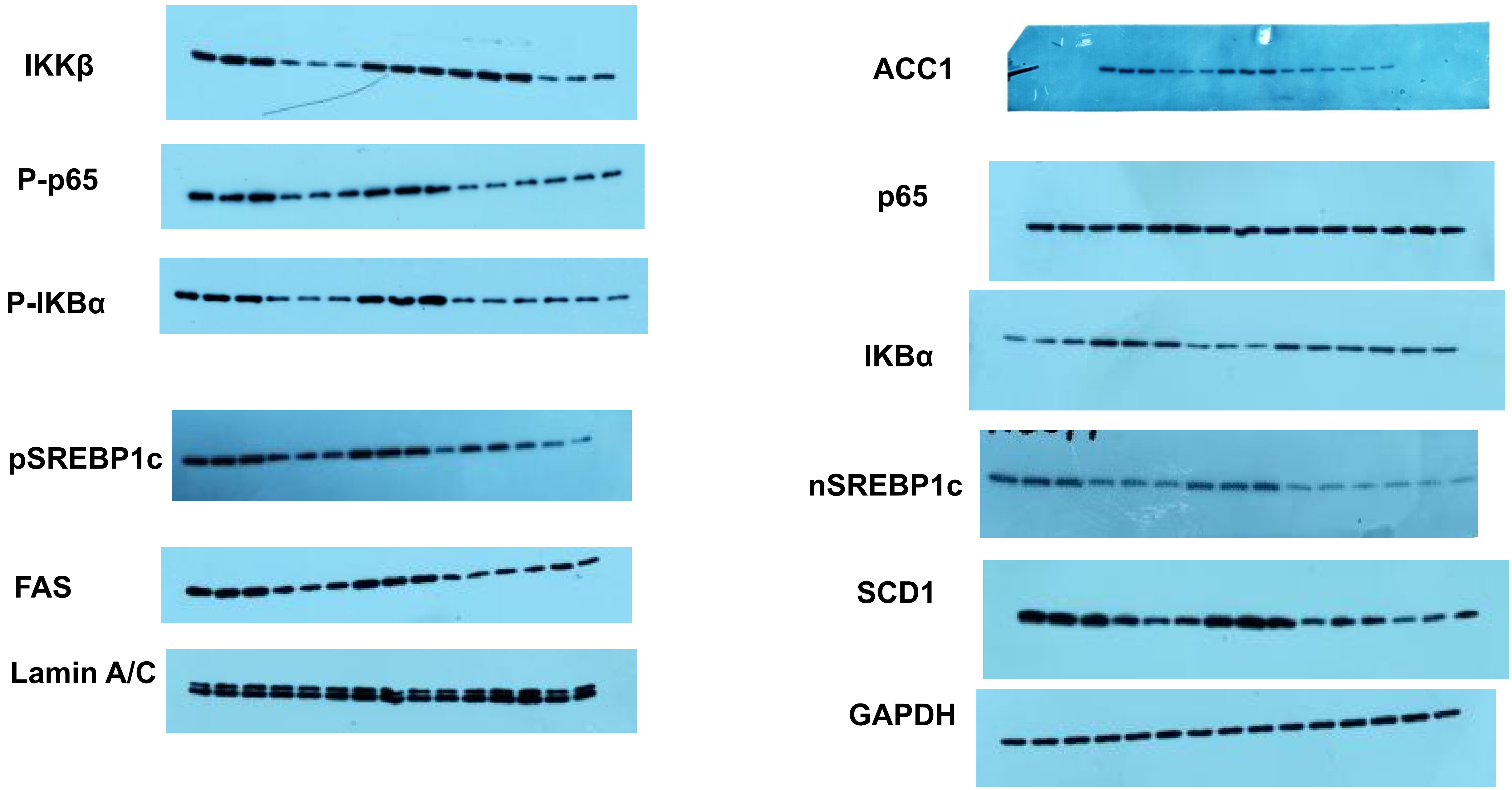

Full unedited blot for FigureS 13A

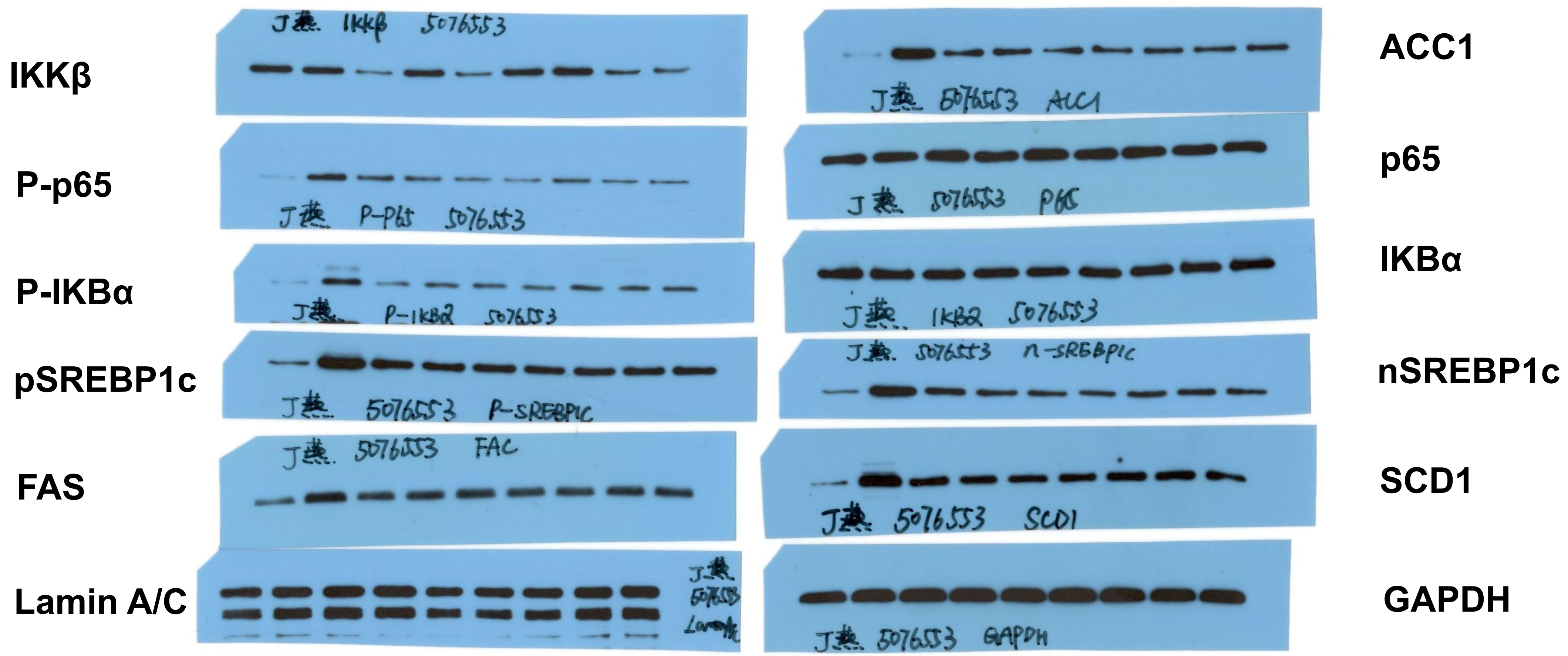

Full unedited blot for FigureS 14A

P-IR

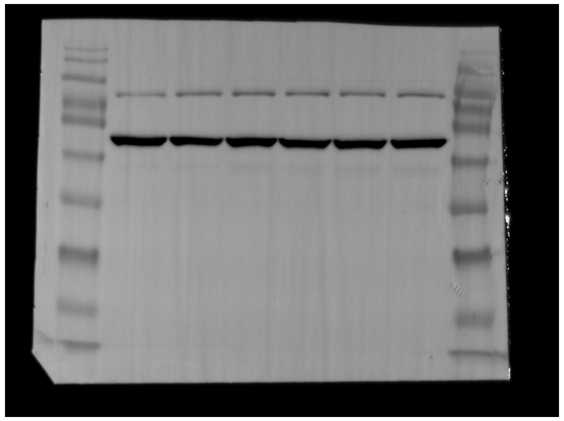

IR

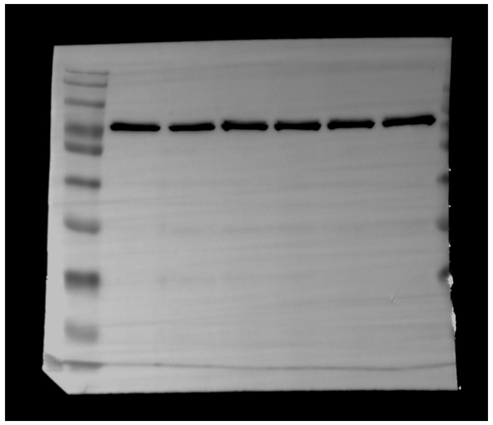

P-Akt

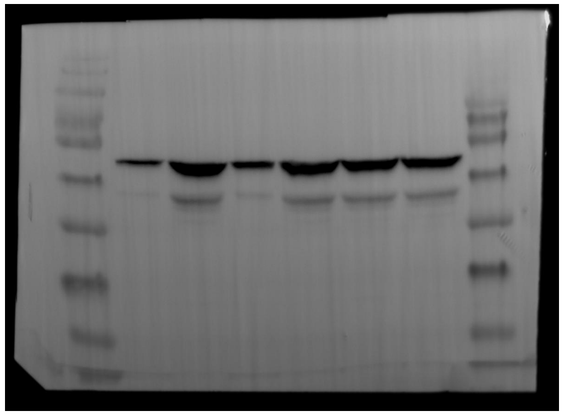

Akt

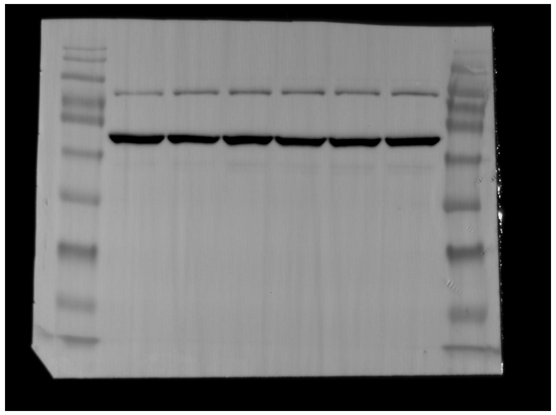

GAPDH

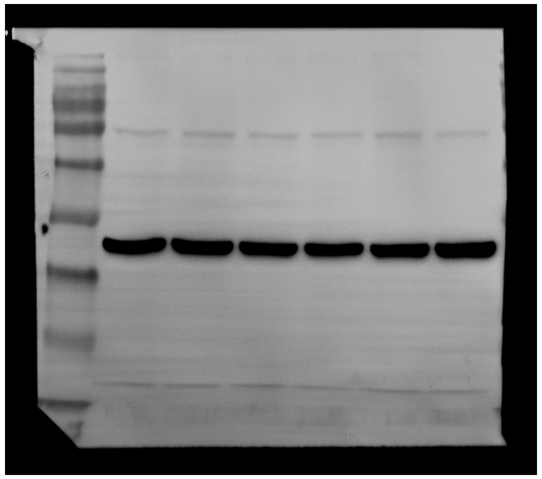

Supplement: Supplementary file 2 — Original Data File [file 41419_2023_5904_MOESM2_ESM.pdf]
