# Supplementary figures and images for: Myeloid-derived growth factor alleviates non-alcoholic fatty liver disease alleviates in a manner involving IKKβ/NF-κB signaling
Source: Cell Death Dis. 2023 Jun 26;14(6):376. doi: 10.1038/s41419-023-05904-y (PMC10293205; doi:10.1038/s41419-023-05904-y)

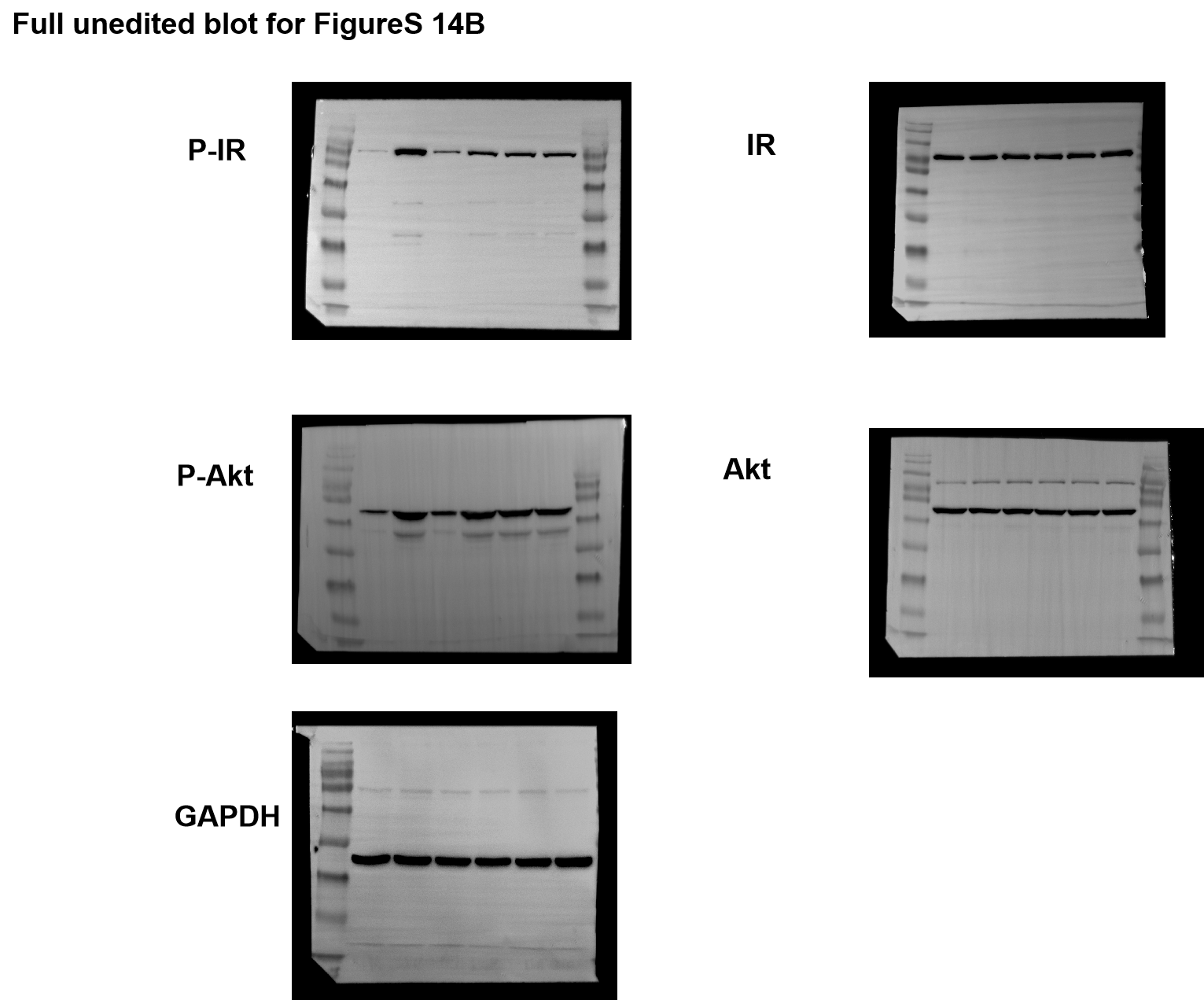

Supplement: Supplementary file 3 — Figure S14B [file 41419_2023_5904_MOESM3_ESM.tif]
